# Amyloid PET and clinical management in a diverse, cognitively impaired population: The New IDEAS Study

**DOI:** 10.1002/alz.70504

**Published:** 2025-07-29

**Authors:** Charles C. Windon, Constantine Gatsonis, Maria C. Carrillo, Justin Romanoff, Lucy Hanna, Emily Glavin, Ilana Gareen, Roee Gutman, Bruce E. Hillner, Andrew March, Sid O'Bryant, Robert A. Rissman, Barry A. Siegel, Karen Smith, Rachel A. Whitmer, Christopher J. Weber, Consuelo H. Wilkins, Peggye Dilworth‐Anderson, Gil D. Rabinovici

**Affiliations:** ^1^ Department of Neurology Memory and Aging Center Weill Institute for Neurosciences University of California, San Francisco San Francisco California USA; ^2^ Center for Biostatistics and Health Data Science Brown University School of Public Health Providence Rhode Island USA; ^3^ Department of Biostatistics Brown University School of Public Health Providence Rhode Island USA; ^4^ Alzheimer's Association Chicago Illinois USA; ^5^ Center for Research and Innovation American College of Radiology Reston Virginia USA; ^6^ Department of Epidemiology Brown University School of Public Health Providence Rhode Island USA; ^7^ Department of Medicine Virginia Commonwealth University Richmond Virginia USA; ^8^ Institute for Translational Research University of North Texas Health Science Center Fort Worth Texas USA; ^9^ Department of Pharmacology and Neuroscience University of North Texas Health Science Center Fort Worth Texas USA; ^10^ Department of Physiology and Neuroscience Alzheimer's Therapeutic Research Institute Keck School of Medicine of the University of Southern California San Diego California USA; ^11^ Edward Mallinckrodt Institute of Radiology Washington University School of Medicine St. Louis Missouri USA; ^12^ Division of Research Kaiser Permanente Oakland California USA; ^13^ Department of Public Health Sciences University of California Davis Davis California USA; ^14^ Department of Medicine Division of Geriatric Medicine Vanderbilt University Medical Center Nashville Tennessee USA; ^15^ Health Policy and Management Gillings School of Global Public Health University of North Carolina at Chapel Hill Chapel Hill North Carolina USA; ^16^ Department of Radiology and Biomedical Imaging University of California, San Francisco San Francisco California USA

**Keywords:** Alzheimer's disease, amyloid positron emission tomography, dementia, ethnicity, Medicare, mild cognitive impairment

## Abstract

**INTRODUCTION:**

The New Imaging Dementia–Evidence for Amyloid Scanning (IDEAS) study (NCT04426539) evaluated the association between amyloid positron emission tomography (PET) and changes in clinical management among ethnoracially diverse, clinically heterogeneous patients.

**METHODS:**

We assessed diagnosis and management plan before and 90 ± 30 days after amyloid PET among Medicare beneficiaries who met 2018 National Institute on Aging–Alzheimer's Association criteria for mild cognitive impairment (MCI) or dementia. We aimed to identify ≥ 30% change in a composite patient management endpoint (CPME; i.e., changes in Alzheimer's disease [AD]/non‐AD medications, changes in counseling).

**RESULTS:**

Among 5757 participants (median age 75 years; 21.7% Black, 20.3% Latinx, 58.1% all other races/ethnicities [AORE]), a change in CPME occurred in 59.0% (95% confidence interval 57.6%–60.5%) of individuals post PET. Change varied by ethnoracial identity and type of clinical presentation: Black (MCI: 55.3%, dementia: 55.8%), Latinx (MCI: 53.7%, dementia: 61.9%), AORE (MCI: 62.0%, dementia: 58.3%), typical (MCI: 64.8%, dementia: 60.9%), atypical (MCI 45.5%, dementia: 53.6%).

**DISCUSSION:**

Amyloid PET is associated with clinical management among diverse, clinically heterogeneous populations.

**Highlights:**

- Changes in management plan occurred in 59% of patients 90 days after amyloid positron emission tomography.
- Rates of change in management exceeded the pre‐specified goal of > 30% across ethnoracial groups.
- Rates of change in management also exceeded > 30% among amnestic and non‐amnestic Alzheimer's disease presentations.

## 1 BACKGROUND

Diagnosing Alzheimer's disease (AD) has shifted to an approach that incorporates detailed assessments of biological markers of disease including measures of amyloid and tau. A diagnosis made without biomarkers has limited accuracy and low specificity when considering clinicopathological data.^1^, ^2^, ^3^, ^4^, ^5^ Cerebrospinal fluid (CSF) and plasma biomarkers along with positron emission tomography (PET) using radiotracers designed to bind amyloid plaques and tau neurofibrillary tangles have contributed toward greater diagnostic accuracy and better understanding of disease progression.^6^, ^7^, ^8^, ^9^, ^10^ Currently, three amyloid radiotracers (^18^F‐florbetapir, ^18^F‐flutemetamol, and ^18^F‐florbetaben) are approved by the US Food and Drug Administration (FDA) and other regulatory agencies worldwide for clinical use, and amyloid PET has high sensitivity (florbetapir > 93%, flutemetamol > 90%, florbetaben > 97%) and specificity (florbetapir 100%, flutemetamol > 90%, florbetaben > 88%) based on PET‐to‐autopsy studies.^11^, ^12^, ^13^, ^14^

By 2060, ≈ 13.8 million people in the United States will be living with AD. Annual incidence numbers will grow to ≈ 1 million/year with estimated increases of 423% among Latinx individuals, 192% among Black individuals, and 63% among White individuals.^15^, ^16^ Inequities exist: Black older adults are twice as likely, and Latinx older adults 1.5 times as likely, to have clinical AD and related dementias (ADRD) compared to White older adults.^15^, ^17^, ^18^ However, AD biomarker research is notable for limited inclusion of ethnoracially diverse individuals.^17^, ^19^, ^20^, ^21^, ^22^ A combination of individual, researcher, study‐level, and systemic barriers drive this lack of inclusivity that hinders the generalizability of research findings.^17^, ^18^ There is also a lack of research exploring whether use of AD biomarkers, like amyloid PET, among ethnoracially diverse populations results in meaningful changes in clinical management due to greater diagnostic clarity.

Across many clinical care settings inclusive of diverse patients, absence of AD biomarker testing impacts diagnostic accuracy. Clinical presentations of ADRD alone are not reliable predictors of underlying neuropathology given the heterogeneity of AD and non‐AD syndromes. High rates of negative amyloid PET have been found even among individuals with clinically “typical” presentations of AD, where memory symptoms predominate.^23^, ^24^ Previous work by Rabinovici et al. in the IDEAS (Imaging Dementia–Evidence for Amyloid Scanning) Study, a national, multisite longitudinal study, demonstrated that 63.8% of individuals diagnosed with clinical AD prior to PET have positive amyloid PET and up to 52.2% of individuals with a pre‐PET diagnosis of a non‐AD dementia have positive amyloid PET.^25^

IDEAS demonstrated that amyloid PET was associated with > 60% change in clinical management (between pre‐PET and 90 ± 30 days post‐PET) among Medicare beneficiaries meeting criteria for mild cognitive impairment (MCI) or dementia.^25^, ^26^ The study included > 15,000 individuals but had limited ethnoracial diversity as < 10% of the sample consisted of non‐White individuals.^25^ Individuals had to meet appropriate use criteria for amyloid PET,^27^ which excluded many individuals who had clinically “typical” presentations of AD. Given these gaps, New IDEAS was developed to assess the utility of amyloid PET in an ethnoracially diverse and clinically heterogeneous group of Medicare beneficiaries meeting 2018 National Institute on Aging–Alzheimer's Association (NIA‐AA)^28^ criteria for MCI or dementia. The study aimed to: (1) examine the association between amyloid PET and changes in management among individuals identifying as Black/African American/African (hereafter “Black”), Hispanic/Latino/Spanish (hereafter “Latinx”), and all other races/ethnicities (hereafter “AORE”) and (2) examine the association between amyloid PET and changes in management among individuals with “typical” versus “atypical” clinical presentations of MCI and AD dementia. Pre‐specified objectives for endpoints were to estimate and compare the frequency of 90‐day post‐PET changes in overall management within (1) each ethnoracial subgroup and (2) among “typical” and “atypical” clinical presentation subgroups. We hypothesized frequency of changes in overall management would exceed 30% across all groups.

## 2 METHODS

### 2.1 Study design and management

New IDEAS (NCT04426539) is an observational, prospective, multisite longitudinal cohort study to evaluate the utility of amyloid PET in a broad and diverse population of Medicare beneficiaries meeting 2018 NIA‐AA criteria for MCI or dementia.^28^ The study was conducted in partnership with the Centers for Medicare and Medicaid Services (CMS) under the “Coverage with Evidence Development” (CED) program, which allows coverage of novel technologies for Medicare beneficiaries as part of clinical studies assessing their clinical utility. In the study, participants were enrolled by dementia specialists (defined as a physician trained and board certified in neurology, psychiatry, or geriatric medicine, who devotes ≥ 25% of patient contact time to the evaluation and care of adults with acquired cognitive impairment or dementia) at clinical practices across the United States. Dedicated marketing was performed through professional societies, the Alzheimer's Association, and media outreach to engage dementia specialists to participate. Dementia specialist sites then applied and were invited to participate if they met the site inclusion criteria as detailed in the study protocol (Material S1 in supporting information). Imaging specialists who participated in the visual interpretation of amyloid PET scans performed as part of the study were required to be board certified in diagnostic radiology or nuclear medicine and to have successfully completed vendor‐provided training for interpreting amyloid PET scans. Imaging specialists also had to be enrolled in the Medicare Provider Enrollment, Chain, and Ownership System (PECOS) to provide services to Medicare patients.

The American College of Radiology (ACR) Center for Research and Innovation (CRI) had complete oversight and management of the study and operated alongside a steering committee. New IDEAS was managed by a central institutional review board (Advarra) to ensure compliance with the ethical standards outlined in the 1964 Declaration of Helsinki and its amendments. Authorized site staff obtained written informed consent directly from the patient or, in cases in which the patient lacked capacity to consent, from a legally authorized representative with patient assent during initial eligibility/registration. Sociodemographic information was also collected during initial eligibility/registration. The full protocol and template consent forms are available in Material S1 and S2 in supporting information.

**RESEARCH‐IN‐CONTEXT**

1. **Systematic review**: We reviewed the literature using traditional search engines (PubMed, Google Scholar) and found amyloid positron emission tomography (PET) use impacts clinical management, but prior research was not inclusive of all ethnoracial groups and individuals with clinically heterogeneous presentations of disease.
2. **Interpretation**: Amyloid PET was associated with a change in the management plan for 59% of individuals, most often impacting the use of Alzheimer's disease medications, even in ethnoracially diverse, clinically heterogeneous populations.
3. **Future directions**: Research investigating long‐term outcomes in patients undergoing amyloid PET, with a particular focus on groups that have historically been underrepresented in clinical studies, is needed and may lead to better outcomes for these patients.

### 2.2 Temporary enrollment pause and early termination

The New IDEAS Study protocol pre‐specified that the study would enroll at least 2000 Black, at least 2000 Latinx, and up to 3000 individuals from all other racial and ethnic (AORE) groups. During the course of the study, rapid enrollment of individuals into the AORE cohort resulted in a temporary enrollment pause of this cohort enacted on March 1, 2022 (223 Black, 175 Latinx individuals enrolled by February 1, 2022, compared to 1892 AORE individuals). On August 19, 2022, the New IDEAS Study team extended the enrollment pause for individuals into the AORE cohort (588 Black, 552 Latinx individuals enrolled by August 18, 2022, compared to 2463 AORE individuals) before ultimately reopening enrollment into this cohort on September 1, 2023 only to dementia specialist sites that had registered at least 10 individuals since March 1, 2022 (the beginning of the enrollment pause).

On March 1, 2024, the New IDEAS Study team leadership ended study accrual for all participants. This decision was driven by multiple factors, notably CMS retirement of National Coverage Determination (NCD) on amyloid PET and termination of CED as a criterion for coverage of amyloid PET scans on October 13, 2023. Increasing prior authorization denials of study participants’ PET scans, notably among Medicare Advantage beneficiaries, also influenced this decision.

### 2.3 Study population

Dementia specialists enrolled patients who were receiving routine clinical care within their local clinical practices. To ensure a diverse and balanced cohort, the maximum enrollment number of participants by a single referring specialist was limited to 250 with rare single‐site exceptions. To be eligible, participants had to be (1) Medicare beneficiaries with Medicare as primary insurance; (2) meet 2018 NIA‐AA criteria^28^ for MCI or dementia, established by the dementia specialist's diagnosis within 24 months prior to enrollment; (3) able to tolerate amyloid PET according to study protocol; (4) have completed head computed tomography or magnetic resonance imaging (MRI) within the 24 months prior to enrollment; and (5) have completed clinical laboratory testing within the 12 months prior to enrollment. Individuals were classified as having clinical syndromes considered “typical” (i.e., progressive amnestic) or “atypical” (i.e., non‐amnestic) of cognitive impairment caused by AD (see full protocol in Material S1 for additional details). Individuals were excluded if they: (1) had normal cognition; (2) would experience significant psychological harm if they were to become knowledgeable of their amyloid status, based on the opinion of the referring dementia specialist; (3) had previously been informed of their amyloid or tau status through prior workup; or (4) met additional exclusion criteria detailed in the study protocol (Material S1), which notably included current or previous treatment with an anti‐amyloid agent and/or current or previous enrollment in an anti‐amyloid therapeutic trial.

To evaluate the diversity of the study cohort compared to the general Medicare beneficiary population, the dementia specialists or their designees (such as the study coordinator or practice administrator) collected the participants’ self‐identified race and ethnicity. Individuals selected all that applied from the following options: American Indian or Alaskan Native, Asian, Black or African American or African, Hispanic or Latino or Spanish, Native Hawaiian or Pacific Islander, White, Other (no categories fully describe my identity), or Prefer not to answer. Any individual who selected “Black or African American or African” was grouped within the Black/African American/African cohort, regardless of other selections. Any individual who selected “Hispanic or Latino or Spanish” was grouped within the Hispanic/Latino/Spanish cohort, regardless of other selections. Individuals who selected both “Black or African American or African” and “Hispanic or Latino or Spanish” were randomly assigned to one of these two cohorts. If an individual did not select “Black or African American or African” or “Hispanic or Latino or Spanish,” they were grouped within the all other races/ethnicities (AORE) cohort, regardless of their selections. Given the aims of the study, the three cohorts considered in analyses were: Black, Latinx, and AORE. This cohort categorization was selected based on the existence of inequities in ADRD that disproportionately impact Black and Latinx individuals compared to other groups and the limited inclusion of Black and Latinx individuals into ADRD biomarker studies.

### 2.4 Strategies for recruitment of diverse populations

Individuals of all racial and ethnic groups and sexes were eligible for the study, which pre‐specified recruitment caps based on self‐identified race and ethnicity (Black 2000, Latinx 2000, AORE 3000). This study used strategies and tools developed by C. Wilkins and P. Dilworth‐Anderson and previously implemented in the successful recruitment of minority participants into clinical research to enrich for Black and Latinx study participation.^29^, ^30^, ^31^, ^32^, ^33^, ^34^ Recruitment was spearheaded by C. Wilkins and P. Dilworth‐Anderson leveraging resources from the Alzheimer's Association, the ACR, the Vanderbilt Recruitment Innovation Center,^35^, ^36^, ^37^, ^38^ and the University of North Carolina Center for Health Equity Research.^39^, ^40^ Additional details about recruitment strategy will be detailed in a separate forthcoming publication. In brief, the overall approach to optimizing recruitment of Black and Latinx individuals followed a multipronged strategy including: (1) identification of 10 regions across the United States with dementia specialists caring for larger numbers of Black and/or Latinx patients with capacity to engage them; (2) local community engagement efforts in these regions; (3) creation of a centralized source of tools and resources such as culturally adapted recruitment materials, education, and tips on recruitment and engendering trust, and transcreation of recruitment materials in Spanish; (4) a national awareness campaign including social media; and (5) on‐demand support to local sites to engage and retain participants.

### 2.5 Pre‐PET clinical assessments

Dementia specialists captured clinical data prior to amyloid PET according to a pre‐PET clinical assessment form (Material S2) that recorded (1) certification of eligibility; (2) medical history and COVID‐19 exposure; (3) pre‐PET clinical diagnosis (MCI vs. dementia, typical vs. atypical clinical presentation, suspected etiology of impairment, AD drug therapy use); and (4) management plan based on current clinical and diagnostic information, assuming no future access to amyloid PET. The management plan included in the composite management endpoint: (1) use of medications approved for symptomatic treatment of AD (cholinesterase inhibitors, memantine, aducanumab—beginning June 2021, and lecanemab—beginning January 2023 after its FDA approval), (2) use of other medications for management of central nervous system conditions or risk factors (e.g., selective serotonin reuptake inhibitors, etc.), and (3) counseling about safety (e.g., home safety, medication monitoring, driving) and future planning (e.g., medical and financial decision making, advance directives). We additionally collected data about plans for referrals to patient and caregiver support resources, referrals to specialists, and further diagnostic testing.

### 2.6 Acquisition, interpretation, and disclosure of PET results

Trained imaging specialists performed amyloid PET at accredited imaging centers within 60 days after completion of the pre‐PET clinical assessment form by the enrolling dementia specialists. Imaging was performed with one of the three US FDA‐approved radiopharmaceuticals in accordance with established practice guidelines.^41^ Interpretation of the images followed the approved criteria for each tracer. Each radiologist or nuclear medicine physician visually interpreting amyloid PET images as part of the New IDEAS Study was required to have completed vendor‐provided in‐person or online training courses specific to the amyloid imaging agent (or agents) used at their participating PET facility. According to FDA guidelines, a negative or positive categorization was used, with a negative interpretation indicating sparse to no neuritic plaques whereas a positive interpretation indicated moderate to frequent neuritic plaques. Dementia specialists disclosed the results of visual amyloid PET interpretation to patients as part of routine clinical care.

### 2.7 Post‐PET assessments

A clinical office or telemedicine visit with the referring dementia specialist 60 to 120 days after completion of the amyloid PET scan was mandatory. At this visit, the patient management plan was documented, including data on implementation and adherence to management actions recommended at the amyloid PET disclosure visit (or subsequent clinical visits, prior to this mandatory visit) along with additional recommended changes in management (e.g., pharmacological treatments, referrals, counseling, etc.). These data were captured in post‐PET clinical assessment forms (Material S2).

### 2.8 Objectives

As described in the New IDEAS protocol (Material S1), the study involved primary data collection on a diverse cohort of Medicare beneficiaries meeting criteria for MCI or dementia enrolled at clinical practices across the United States and analysis of Medicare claims data available for this cohort. The three main objectives of the overall study were: (1) to compare 12 month claims‐derived health outcomes in amyloid PET positive versus amyloid PET negative individuals presenting with MCI, (2) to examine the association of amyloid PET findings with changes in patient management and 12 month claims‐derived health outcomes among ethnoracial subgroups (Black, Latinx, AORE), presenting with MCI and dementia, and (3) to examine the association of amyloid PET findings with changes in management and 12 month claims‐derived health outcomes in individuals presenting with typical (i.e., progressive amnestic) versus atypical clinical presentations of MCI and AD dementia.

The present article addresses a subset of the three overall protocol objectives. Of note, 12 month Medicare claims‐derived health outcomes data for objective 1 are not yet available. Specifically, this article is based on data collected directly from the participant, their medical care providers, and the imaging center that performed and interpreted the amyloid PET scan. Using these data, the study examines (1) the association between amyloid PET and changes in management among Black, Latinx, and AORE individuals, and (2) the association between amyloid PET and changes in management among individuals with typical versus atypical clinical presentations of MCI and dementia due to AD. For the first set of analyses (Goal 1), the primary objective was to evaluate the rate of change in a composite management endpoint from pre‐PET to 90 days post‐PET within each ethnoracial group, separately for participants with MCI and dementia. We specifically assessed changes in the use of AD and non‐AD medications, and changes in counseling on safety and future planning. As an exploratory objective we assessed frequency of amyloid PET positivity, the proportion of MCI versus dementia, and the use of AD medications at study entry, between our three specified ethnoracial cohorts.

For the second set of analyses (Goal 2), the primary objective was to evaluate the rate of change in a composite management endpoint from pre‐PET to 90 days post‐PET within each AD clinical presentation group (typical vs. atypical), separately for participants with MCI and dementia. We specifically assessed changes in the use of AD and non‐AD medications, and changes in counseling on safety and future planning. As an exploratory objective we assessed the association between changes in the composite management endpoint and several potential predictors, including age, cognitive impairment level, clinical presentation, race, ethnicity, and pre‐existing medical co‐morbidities.

### 2.9 Statistical considerations

**FIGURE 1:**
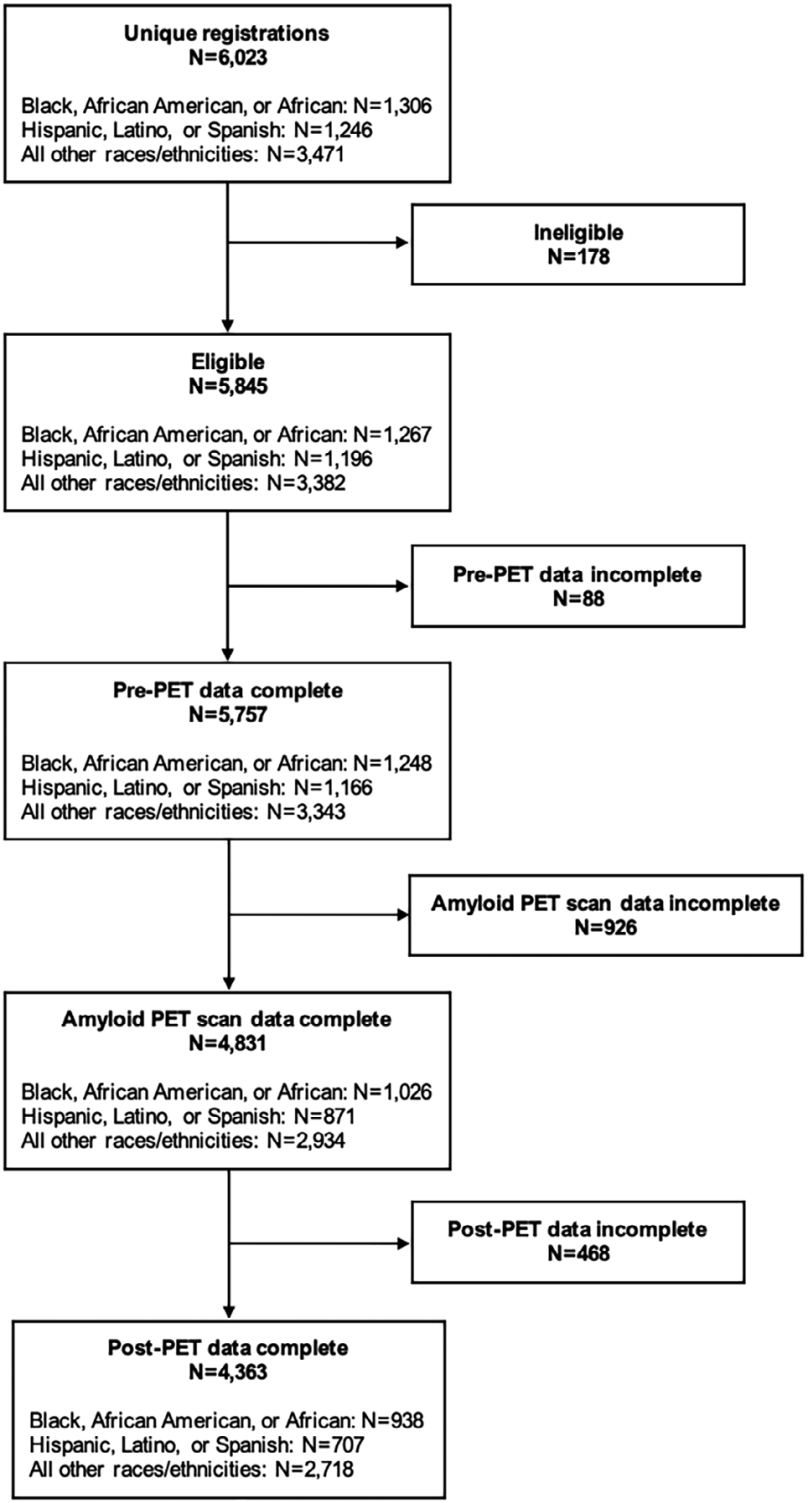
Participant flowchart. PET, positron emission tomography.

The primary outcome is *overall change* in management between the pre‐PET care plan and the 90 day post‐PET care plan, defined as change in one or more of the following items: AD‐specific medications, non‐AD medications related to central nervous system conditions or risk factors, and counseling about safety and future planning. The main analysis presented here uses information from participants with complete pre‐PET data. Multiple imputation was used to account for missing data (Material S3 in supporting information). Briefly, the amyloid PET results were imputed using a logistic regression model with the 76 covariates listed in Material S3. Change in each component of the primary outcome was imputed using separate logistic regression models and the results were combined to derive imputed values of the overall change in management. One hundred imputed data sets were developed using multivariate imputation by chained equations (MICE). The extent of missingness is indicated in Figure 1. Analyses with only the complete data are shown in Tables S1–S2 in supporting information.

For the first goal, the proportion of change in overall management was estimated in each of six cells defined by the three specified ethnoracial categories (Black, Latinx, AORE) and the two levels of impairment (MCI vs. dementia) and was compared to a minimum threshold of 30%. Confidence intervals and *p* values were based on a *t* distribution after multiple imputation. For the second goal, the proportion of change in overall management was estimated in each of the four cells defined by the type of presentation (typical vs. atypical) and level of impairment (MCI vs. dementia). Estimation of proportions was done similarly to the first goal and testing the equality of proportions between typical and atypical participants (separately for participants with MCI and dementia) was done using a *t* test after multiple imputation. To control for multiplicity in the combined testing for the two goals, a Bonferroni approach was used and tests were conducted at level 0.0063 (= 0.05∖8; two sided) to account for the eight simultaneous comparisons.

Logistic regression was used to assess the exploratory aim. The binary response variable was an indicator of change in overall management. The model included main effects as indicated and selected two‐way interactions of interest. A separate Bonferroni control was used to declare significance.

We report three post hoc analyses in the article. First, we performed pairwise comparisons of participant characteristics among the three ethnoracial groups. There was no multiplicity control in this analysis. Second, we examined changes in etiologic diagnosis and provided a descriptive report. Third, we examined the available data on lecanemab use to provide a descriptive report of such use by amyloid PET result of participants.

## 3 RESULTS

### 3.1 Participants

A total of 270 dementia specialists from 151 clinical practices across the United States participated in the study. Imaging was performed at 107 PET facilities and 199 imaging specialists interpreted the PET studies. Among 6023 Medicare beneficiaries registered for this study between December 17, 2020 and March 1, 2024, 5845 met eligibility criteria and 5757 (median age 75 years, range 35–98) had completed pre‐PET data and were included in the final data analysis. Of these, 1248 (807 females, 441 males; median age 74 years, range 35–97) participants were Black, 1166 (724 females, 442 males; median age 75 years, range 41–98) participants were Latinx, and 3343 (1688 females, 1652 males, median age 75 years, range 41–95) participants identified as AORE. There were five individuals who identified as both “Black or African American or African” and “Hispanic or Latino or Spanish,” of whom two were randomly assigned to the Black cohort and three were assigned to the Latinx cohort. Additional details of ethnoracial identities of individuals from the AORE cohort are available in Table S3 in supporting information. There were also two AORE individuals who identified as transgender males. Reasons for participant exclusion were incomplete pre‐PET data (*n* = 88), and other ineligibilities (*n* = 178; Figure 1).

The overall characteristics of participants included in the study sample by ethnoracial group are presented in Table 1, by level of cognitive impairment in Table S4 in supporting information, and by type of clinical presentation in Table S5 in supporting information. The proportion of females was higher in Black (64.7%) and Latinx (62.1%) subgroups, respectively, compared to the AORE subgroup (50.5%). Median ages were similar across groups. Latinx individuals had less educational attainment (54.9% reporting a high school level of education or less) compared to Black (36.3%) and AORE individuals (21.8%). At baseline, 62.6% of participants were diagnosed with MCI (Black 52.2%; Latinx 55.5%; AORE 69.0%) and 30.3% had an atypical presentation of AD (Black 37.5%; Latinx 32.7%; AORE 26.9%). Pre‐PET, 46.0% of individuals were taking AD medications (Black 44.6%; Latinx 44.9%; AORE 46.8%). After multiple imputation, the estimated amyloid PET positivity rate for all individuals was 65.0% (95% confidence interval [CI], 64.4%–65.6%) but lower among Black (60.5%, 58.8%–61.9%) and Latinx (61.1%, 58.8%–63.3%) individuals (AORE individuals (68.1%, 67.5%–68.8%).

**TABLE 1:** Participant characteristics by ethnoracial group.

	Ethnoracial subgroup						
Variable	Black (*N* = 1248)	Latinx (*N* = 1166)	AORE (*N* = 3343)	Total (*N* = 5757)	*p* value Black vs. Latinx	*p* value Black vs. AORE	*p* value Latinx vs. AORE
Median age (IQR, range), years	74 (68–79, 35–97)	75 (70–80, 41–98)	75 (71–80, 41–95)	75 (70–80, 35–98)	<0.001	<0.001	0.26
Sex, *N* (%)					0.20	<0.001	<0.001
Female	807 (64.7)	724 (62.1)	1688 (50.5)	3219 (55.9)			
Male	441 (35.3)	442 (37.9)	1652 (49.4)	2535 (44.0)			
Transgender male	0 (0.0)	0 (0.0)	2 (0.1)	2 (0.0)			
Prefer not to answer	0 (0.0)	0 (0.0)	1 (0.0)	1 (0.0)			
Highest level of education completed, *N* (%)					<0.001	<0.001	<0.001
High school graduate/equivalence or below	453 (36.3)	640 (54.9)	728 (21.8)	1,821 (31.6)			
Some college or associate degree	382 (30.6)	239 (20.5)	785 (23.5)	1,406 (24.4)			
Bachelor's degree	208 (16.7)	175 (15.0)	913 (27.3)	1,296 (22.5)			
Postgraduate degree	205 (16.4)	112 (9.6)	917 (27.4)	1,234 (21.4)			
Median MMSE score (IQR)	21 (19‐25)	22 (19‐26)	25 (22‐28)	24 (20‐27)	0.23	<0.001	<0.001
Median MoCA score (IQR)	18 (13‐22)	18 (12‐21)	21 (18‐24)	20 (15‐23)	0.12	<0.001	<0.001
Level of cognitive impairment, *N* (%)					0.11	<0.001	<0.001
MCI	652 (52.2)	647 (55.5)	2,307 (69.0)	3,606 (62.6)			
Dementia	596 (47.8)	519 (44.5)	1,036 (31.0)	2,151 (37.4)			
Presentation of cognitive impairment, *N* (%)					0.01	<0.001	<0.001
Atypical	468 (37.5)	381 (32.7)	898 (26.9)	1,747 (30.3)			
Typical	780 (62.5)	785 (67.3)	2,445 (73.1)	4,010 (69.7)			
Pre‐PET primary differential diagnosis for cause of cognitive impairment, *N* (%)					<0.001	0.71	<0.001
AD	1,060 (84.9)	1,061 (91.0)	2,855 (85.4)	4,976 (86.4)			
Non‐AD	188 (15.1)	105 (9.0)	488 (14.6)	781 (13.6)			
Pre‐PET taking AD drugs^*^, *N* (%)					0.90	0.19	0.28
Yes	557 (44.6)	524 (44.9)	1,565 (46.8)	2,646 (46.0)			
No	691 (55.4)	642 (55.1)	1,778 (53.2)	3,111 (54.0)			
Amyloid PET scan result, *N* (%)					<0.001	<0.001	<0.001
Positive	625 (50.1)	532 (45.6)	2,006 (60.0)	3,163 (54.9)			
Negative	406 (32.5)	345 (29.6)	933 (27.9)	1,684 (29.3)			
Missing^**^	217 (17.4)	289 (24.8)	404 (12.1)	910 (15.8)			

Abbreviations: AD, Alzheimer's disease; AORE, all other races/ethnicities; IQR, interquartile range; MCI, mild cognitive impairment; MMSE, Mini‐Mental State Examination; MoCA, Montreal Cognitive Assessment; PET, positron emission tomography.

* The AD drugs that participants could have been taking at the time of the pre‐PET visit include cholinesterase inhibitors and memantine. No participants were taking anti‐amyloid therapeutics at the time of the pre‐PET visit (though such treatment might have been recommended to some subjects).

** The analysis includes the results of amyloid PET scans on 16 participants which were completed past the defined time window.

### 3.2 Changes in management

An overall change in management (composite of change in AD medications, change in non‐AD medications, change in counseling) from pre‐ to post‐PET, was observed in 59.0% (95% CI, 57.6%–60.5%) of participants. Most often, these changes were related to AD medications (42.9%, 41.4%–44.4%), followed by changes in counseling (22.4%, 21.1%–23.6%) and changes in non‐AD medications (19.1%, 18.0%–20.3%; Table 2).

**TABLE 2:** Change in management by ethnoracial subgroup, level of cognitive impairment, and presentation of cognitive impairment after multiple imputation.

	Overall change in management		Change in AD drugs	Change in non‐AD drugs	Change in counseling
	% (95% CI)	*p* value^a^	% (95% CI)	% (95% CI)	% (95% CI)
Ethnoracial subgroup					
Black (*N* = 1248)					
MCI (*N* = 652)	55.3 (51.1, 59.5)	<0.001	38.7 (34.5, 42.8)	18.2 (15.0, 21.4)	25.2 (21.5, 29.0)
Dementia (*N* = 596)	55.8 (51.3, 60.3)	<0.001	37.1 (32.7, 41.5)	20.9 (17.2, 24.5)	16.9 (13.4, 20.4)
Latinx (*N* = 1166)					
MCI (*N* = 647)	53.7 (49.2, 58.2)	<0.001	37.6 (33.1, 42.0)	16.6 (13.1, 20.1)	24.4 (20.4, 28.4)
Dementia (*N* = 519)	61.9 (56.7, 67.0)	<0.001	41.1 (35.9, 46.4)	23.5 (19.0, 28.1)	22.5 (18.1, 27.0)
AORE (*N* = 3343)					
MCI (*N* = 2307)	62.0 (59.9, 64.2)	<0.001	47.4 (45.1, 49.6)	18.9 (17.2, 20.6)	24.3 (22.4, 26.2)
Dementia (*N* = 1036)	58.3 (55.0, 61.6)	<0.001	43.2 (39.8, 46.5)	18.5 (15.9, 21.1)	18.0 (15.4, 20.6)
Level and presentation of cognitive impairment					
Atypical, MCI (*N* = 1028)	45.5 (42.1, 48.9)	<0.001	30.3 (27.2, 33.5)	15.1 (12.7, 17.5)	19.3 (16.6, 22.0)
Typical, MCI (*N* = 2578)	64.8 (62.8, 66.8)	<0.001	49.5 (47.3, 51.6)	19.7 (18.0, 21.4)	26.6 (24.7, 28.5)
Atypical, dementia (*N* = 719)	53.6 (49.5, 57.6)	<0.001	34.0 (30.2, 37.9)	21.4 (18.0, 24.7)	18.8 (15.5, 22.1)
Typical, dementia (*N* = 1432)	60.9 (58.1, 63.8)	<0.001	44.5 (41.4, 47.5)	19.8 (17.5, 22.2)	18.8 (16.3, 21.2)
Total (*N* = 5757)	59.0 (57.6, 60.5)		42.9 (41.4, 44.4)	19.1 (18.0, 20.3)	22.4 (21.1, 23.6)

Abbreviations: AD, Alzheimer's disease; AORE, all other races/ethnicities; CI, confidence interval; MCI, mild cognitive impairment.

^1^ After applying the Rubin rules to 100 multiply imputed datasets, *p* values were calculated using a one‐sample *t* test to test the null hypothesis that the true overall change in management is 30% (for each combination of ethnoracial group and level of cognitive impairment, and for each combination of presentation of cognitive impairment and level of cognitive impairment).

Examining ethnoracial groups, among individuals with MCI the overall change in management was greatest among AORE individuals (62.0%, 59.9%–64.2%), followed by Black individuals (55.3%, 51.1%–59.5%) and Latinx individuals (53.7%, 49.2%–58.2%), though all exceeded the null hypothesis of a 30% change (all *p* < 0.001). Among individuals with dementia level impairment, overall change in management again exceeded 30% across groups (*p* < 0.001); however, Latinx individuals had greatest change (61.9%, 56.7%–67.0%), followed by AORE individuals (58.3%, 55.0%–61.6%) and Black individuals (55.8%, 51.3%–60.3%; Table 2).

Examining clinical presentation of AD, overall change in management among individuals with MCI occurred significantly less among atypical (45.5%, 42.1%–48.9%) compared to typical (64.8%, 62.8%–66.8%) presentations (*p* < 0.001). Likewise, among individuals with dementia, overall change in management occurred significantly less among atypical (53.6%, 49.5%–57.6%) compared to typical (60.9%, 58.1%–63.8%) presentations (*p* < 0.001; Table 2).

Additional details of changes in management by ethnoracial group, level of impairment, and by presentation of cognitive impairment are detailed in Tables S6‐S8 in supporting information.

### 3.3 Changes in etiologic diagnosis

The diagnosis changed from AD to non‐AD in 25.5% (95% CI 22.8%–28.2%) of Black participants, 25.6% (22.6%–28.7%) of Latinx participants, and 22.2% (20.7%–23.7%) of AORE participants. The diagnosis changed from non‐AD to AD in 6.8% (5.3%–8.2%) of Black participants, 4.6% (3.3%–5.8%) of Latinx participants, and 7.3% (6.4%–8.2%) of AORE participants (Table S9 in supporting information).

### 3.4 Multivariate analysis of factors associated with changes in management

PET positivity was significantly associated with an overall change in management (Table 3). The strength of the association depended on the level of impairment with odds ratio (OR) 1.43 (95% CI: 1.12–1.83) for participants with dementia and OR 3.02 (2.53–3.59) for participants with MCI (reference negative amyloid PET). Female sex was also significantly associated with an increase in the probability of overall change in management: (OR 1.26, 1.10–1.44, *p* < 0.001). There were no significant associations between age, level of education, pre‐PET use of AD medications, pre‐PET differential diagnosis, atypical presentation, and ethnoracial identity with the overall change in management (Table 3).

**TABLE 3:** Logistic regression model assessing factors associated with overall change in management after multiple imputation.

Variable	OR (95% CI)	*p* value
Intercept	0.78 (0.35, 1.76)	0.55
Age (10 years)	1.02 (0.92, 1.13)	0.73
Female sex (reference: male)	1.26 (1.10, 1.44)	<0.001
Bachelor's degree or higher (reference: did not receive bachelor's degree)	1.12 (0.98, 1.29)	0.10
Pre‐PET taking AD drugs (reference: no AD drugs)	0.94 (0.82, 1.09)	0.44
Pre‐PET primary differential diagnosis of AD (reference: non‐AD)	0.85 (0.69, 1.05)	0.14
Patient has dementia (reference: MCI)	1.56 (1.20, 2.02)	<0.001
Atypical presentation (reference: typical)	0.85 (0.72, 1.02)	0.08
Ethnoracial cohort (reference: AORE)		
Black	0.96 (0.80, 1.16)	0.67
Latinx	1.06 (0.86, 1.31)	0.56
Positive amyloid PET scan result (reference: negative)	3.02 (2.53, 3.59)	<0.001
Interaction: level of impairment x positive amyloid PET scan result (reference: negative)		<0.001
Dementia	1.43 (1.12, 1.83)	
MCI	3.02 (2.53, 3.59)	

Abbreviations: AD, Alzheimer's disease; AORE, all other races/ethnicities; CI, confidence interval; MCI, mild cognitive impairment; OR, odds ratio; PET, positron emission tomography.

*Note*: Analysis set (*N* = 5754) excluded two participants who identified as transgender male and one participant who did not provide their sex.

### 3.5 Lecanemab use based on amyloid PET scan result

Lecanemab, a disease‐modifying therapy for treating early‐stage AD, received accelerated approval in the United States by the FDA on January 6, 2023 (traditional approval occurred July 6, 2023).^42^ New IDEAS captured changes in lecanemab use before and after amyloid PET among participants enrolled post approval. Prior to amyloid PET completion, no individuals were actively taking lecanemab. Notably, 9.7% of Black individuals experienced a change in the use of anti‐amyloid therapeutics, compared to 10.5% of Latinx individuals, and 16.3% of AORE individuals (Table S6). Overall, after positive amyloid PET, the treatment plan for 14 patients changed from recommended use to active use of lecanemab, while 233 individuals who had not previously been advised to take lecanemab were now either taking lecanemab or recommended to take lecanemab. Change from “recommended use” to “active use” was considered a significant clinical change as PET result would have had a significant role in this decision. The management plan regarding lecanemab use for 532 patients did not change after positive amyloid PET (Table S10 in supporting information).

## 4 DISCUSSION

In this study of an ethnoracially diverse population of Medicare beneficiaries who met 2018 NIA‐AA criteria for MCI or dementia from across the United States, > 50% of patients had a change in their management plans after amyloid PET, exceeding the pre‐specified objective endpoint of 30% change. The most frequent changes, across all ethnoracial and clinical presentation subgroups, were related to AD medication use, followed by modifications to non‐AD medications, and finally changes to counseling. Changes in overall diagnosis (AD to non‐AD or non‐AD to AD) occurred for 32.4% of Black participants, 29.4% of Latinx participants, and 29.5% of AORE participants. Overall, we replicated findings from the original IDEAS study^25^ within a more inclusive cohort, extending the generalizability of the results and further validating the benefits of amyloid PET in a real‐world care setting. Our study adds to a growing body of evidence supporting a clinical role for amyloid PET in the diagnosis and management of patients with cognitive impairment. Building on data from the IDEAS study, other clinical utility studies including AMYPAD‐DPMS (Amyloid Imaging to Prevent Alzheimer's Disease—Diagnostic and Patient Management Study),^43^ as well as the emergence of novel amyloid‐targeting therapies that require biomarker confirmation of amyloid for treatment eligibility, CMS retired its national coverage decision restricting amyloid PET reimbursement to approved CED studies. This enabled reimbursement for clinical use and thereby increased access to this diagnostic tool for Medicare patients.

Our examination of individual ethnoracial cohorts revealed across all groups the overall change in management exceeded the pre‐specified objective endpoint of 30% change. Our multivariate model demonstrated the strength of association of PET positivity with overall change in management was stronger for individuals with dementia level impairment (compared to MCI) and importantly Black and Latinx ethnoracial identity were not associated with significantly lower odds of change in management. This finding highlights the potential impact of amyloid PET on clinical management among Black and Latinx individuals presenting for dementia specialist care, particularly given they are often diagnosed with greater impairment by the time they access specialist care due to multiple factors (e.g., inadequate care access in local environments, provider‐level biases, etc.).^44^, ^45^, ^46^ We believe our study is the first to demonstrate this among a cohort of this size of Black and Latinx individuals from real‐world specialty clinics across the United States. This finding emphasizes that the utility of amyloid PET in clinical decision making is applicable for all individuals, regardless of ethnoracial identity, and that the primary challenge for minority populations continues to be lack of access to biomarker testing due to a multitude of structural barriers. Increasing access to amyloid PET and other AD biomarker testing has significant implications for management outcomes among these populations based on our results.

Likewise, our examination of varying clinical presentations of MCI and dementia revealed that across atypical and typical presentations the overall change in management exceeded the pre‐specified objective endpoint of 30% change, demonstrating the value of amyloid PET as a diagnostic tool across the clinical spectrum. Overall change in management was > 60% among individuals with typical presentations of MCI and dementia, suggesting amyloid PET has significant impact even when clinical presentation follows a progressive amnestic course that is often observed in AD. We also found that more than half (55.6%) of individuals with atypical presentations were PET positive. Clinicopathological studies suggest many patients have a neuropathological cause of cognitive impairment that differs from clinician suspicion,^1^, ^47^ leading to a misdiagnosis of AD even when they present with “typical” amnestic features suggestive of AD. The clinical diagnosis of late‐onset AD is also only ≈ 70% accurate, according to some clinicopathological studies.^1^, ^48^ While the original Appropriate Use Criteria emphasized a clinical role for amyloid PET in patients presenting with atypical clinical syndromes,^27^ a recent update has broadened the spectrum of appropriate clinical use to include patients presenting with “typical” clinical presentations.^49^ Our data support this update by highlighting high rates of changes in clinical management in patients with both atypical and typical clinical presentations of AD.

Cross‐sectional studies have shown that plasma biomarkers like reduced amyloid beta 42/40 ratios and elevated concentrations of phosphorylated tau (p‐tau) at positions 181, 217, and 231 are associated with amyloid positivity and amyloid load as measured by PET and CSF.^50^, ^51^, ^52^, ^53^, ^54^ Plasma biomarkers thus are highly promising tools for accurate assessment of amyloid pathology, are already being used extensively in research studies and clinical trials, and will likely have an important diagnostic role once validated in more diverse and heterogenous populations in both clinical practice and clinical trials.^55^ Recent FDA approval of a blood‐based biomarker is a testament to the progress that has been made in accuracy of blood‐based assays. Though blood‐based biomarkers will likely be widely adopted into clinical settings given their scalability, PET may continue to offer key advantages in certain clinical scenarios (e.g., topographical visualization of amyloid burden, indeterminate blood‐based biomarker results, impact of renal insufficiency and body mass index on blood‐based biomarkers, etc.). Current appropriate use criteria (AUC) also include numerous clinical scenarios that are indications for amyloid and tau PET.^49^ AUC for lecanemab and donanemab also suggest preference of PET or CSF over blood‐based biomarkers, though this may change.^56^, ^57^ New IDEAS also collected plasma from ≈ 1500 participants as an optional component of the study, and future work will investigate plasma biomarkers in this unique cohort of individuals receiving “real world” dementia specialty care.

We observed lower amyloid PET positivity rates among Black and Latinx individuals compared to AORE individuals, mirroring findings from the original IDEAS study^58^ and from other observational cohorts and clinical trials.^59^, ^60^ A possible explanation is the greater contribution of non‐AD pathology (e.g., cerebrovascular disease) to cognitive impairment among Black and Latinx individuals, driven by varying dementia risk profiles (e.g., presence/absence of vascular risk factors, etc.).^61^, ^62^, ^63^ Adverse social determinants of health (SDOH), the conditions of the neighborhoods in which we are born, age, work, and live, are disproportionately more common among Black and Latinx individuals and are associated with dementia risk factors like vascular disease. Specific aspects related to SDOH in New IDEAS are being investigated further and will be explored in detail in a forthcoming manuscript.

Despite lower rates of amyloid PET positivity, we found that a greater proportion of Black and Latinx individuals had dementia (versus MCI) at the time of study entry, compared to AORE individuals. These results are in line with previous research, including findings from the original IDEAS study.^58^, ^64^, ^65^ Several epidemiological studies have found a higher prevalence of dementia among Black and Latinx individuals compared to non‐Latinx White individuals.^66^, ^67^, ^68^ These findings are the result of complex factors that determine a patient's level of impairment at diagnosis, which include SDOH that drive variability in risk factors for disease, access to health care, timeliness of presentation for medical care, and other entities. It is important to highlight that there is no biological basis for racial and ethnic categorization in the United States given that these categories are created social constructs.^21^, ^69^ Thus, differences between groups need to be interpreted appropriately and not as the result of inherent biological variations that exist between racial and ethnic identities.

The amyloid‐targeting monoclonal antibody lecanemab received traditional FDA approval and CMS coverage in July 2023, allowing us to capture early data on the use of this novel medication toward the latter part of our enrollment window. Clinicians in our study, based on PET results, made changes in the care plan and recommended the use of lecanemab to > 10% of patients. We believe these data represent some of the earliest “real‐world” prescriber data for lecanemab. Our findings also illustrate the complexity of decision making involved in anti‐amyloid therapy use. Additional biomarkers and patient factors may have informed decisions about lecanemab use beyond PET scan result alone. Lecanemab and other amyloid‐targeting therapies are resource‐intensive treatments that require significant infrastructure and updating of clinical care pathways even at highly experienced specialty care settings.^56^ It is likely that most clinical sites were not yet fully equipped to offer this therapy in the narrow window during which it was available in New IDEAS. Future studies will capture a larger effect of amyloid PET on prescription of molecular‐specific therapies. Nevertheless, our findings suggest that access to amyloid PET influenced lecanemab use recommendations and that PET in a multimodal assessment may reduce the risk of misdiagnosis, personalize treatment more effectively, and ensure that therapies are prescribed when appropriate.

Our study has multiple strengths. By actively recruiting a wide range of individuals from diverse ethnoracial and socioeconomic backgrounds, we provide multiple insights into ADRD management across populations that have not been adequately represented in prior research. The large number of participants and specialists, and the performance of PET at clinical practices, generated a substantial real‐world data set that can be used to identify patterns and trends related to ADRD diagnosis and management. Our study also has limitations. We did not directly assess management changes driven by other diagnostic tools, nor did we perform a comparison to those without amyloid PET. Our categorization of individuals into three ethnoracial groups may oversimplify the significant heterogeneity seen within each of these groups. We experienced recruitment challenges and enacted a study enrollment pause. We did not capture additional detailed provider characteristics that could impact management nor additional details of clinical presentation of disease.

Several recent studies have highlighted the validity of amyloid PET in routine clinical practice, with evidence showing that it leads to a shift in etiological diagnosis, enhances diagnostic confidence, and results in changes to patient management.^70^, ^71^, ^72^, ^73^, ^74^, ^75^, ^76^ However, as demonstrated in our study, individual and group differences may exist, particularly given the significant gap in the inclusion of underrepresented populations in research. Addressing these gaps is essential to ensure that diagnostic advances benefit all populations equitably, leading to more accurate and inclusive management strategies for all individuals with cognitive impairment.

## CONFLICT OF INTEREST STATEMENT

CCW reports receiving grants from the Alzheimer's Association and the NIA. He has received honorariums from LCN, Kinetix Group, and Onviv Inc. CG and BEH report support from the American College of Radiology. JR, LH, and KS were supported by the American College of Radiology through institutional funding. MCC is a full‐time employee of the Alzheimer's Association. EG and AM are salaried employees of the American College of Radiology. IFG and RG report support from the Alzheimer's Association and the American College of Radiology. RAR reports research support from the National Institute on Aging and the Alzheimer's Association and is a consultant for Amydis Inc, Bioivt, Lexeo, Keystone Bio, Allyx, DiamiR, Ionis, and PrecisionMed. BAS reports receiving grants to institution from the American College of Radiology (ACR) during the conduct of the study; personal fees from Avid Radiopharmaceuticals, Curium Pharma, Progenics Pharmaceuticals, Lantheus Medical Imaging, the American Medical Foundation for Peer Review and Education, Siemens Healthineers (for spouse), ACR (also for spouse), Capella Imaging, ECOG‐ACRIN Medical Research Foundation (also for spouse), Evicore Healthcare (for spouse), GE Healthcare, and Radiological Society of North America (also for spouse) outside the submitted work; and grants from Curium Pharma, Progenics Pharmaceuticals, ImaginAb, and Blue Earth Diagnostics outside the submitted work. RAW reports grants from the NIH and NIA and consulting fees from Genentech PanNeuro. She is the epidemiology section editor for *Alzheimer's and Dementia*. CJW is a full‐time employee of the Alzheimer's Association. CHW reports grants from the NIH, PCORI, and the American College of Radiology. GDR received research support from Genentech. He has served as a paid scientific consultant for Alector, Bristol Myers Squibb, C2N, Avid Radiopharmaceuticals, Eli Lilly, Johnson & Johnson, Merck, Novo Norodisk, Roche. He is an associate editor for *JAMA* and *JAMA Neurology*. All other authors report no conflicts of interest relevant to this work. Author disclosures are available in the supporting information.

## CONSENT STATEMENT

Authorized site staff obtained written informed consent directly from the patient or, in cases in which the patient lacked capacity to consent, from a legally authorized representative with patient for all human subject participants in this study.

## Supporting information



Supporting Information

Supporting Information

Supporting Information

Supporting Information

Supporting Information

Supporting Information

Supporting Information

Supporting Information

Supporting Information

Supporting Information

Supporting Information

Supporting Information

Supporting Information

Supporting Information
