# Supplementary material for: Amyloid PET and clinical management in a diverse, cognitively impaired population: The New IDEAS Study
Source: Alzheimers Dement. 2025 Jul 29;21(7):e70504. doi: 10.1002/alz.70504 (PMC12305457; doi:10.1002/alz.70504)
Supplement: Supplementary file 1 — Supporting Information [file ALZ-21-e70504-s014.pdf]

New IDEAS: Imaging Dementia—Evidence for Amyloid Scanning Study
A Study to Improve Precision in Amyloid PET Coverage and Patient Care

**Directed by:**	**Alzheimer's Association**
**Sponsored and Managed by:**	**American College of Radiology**
**Advised by:**	**Centers for Medicare & Medicaid Services (CMS)**

**Study Chair**<br>Gil D. Rabinovici, M.D.<br>University of California, San Francisco<br>(415) 514-2374<br>Gil.Rabinovici@ucsf.edu	**Co-Chair**<br>Maria C. Carrillo, Ph.D.<br>Alzheimer's Association<br>(312) 335-5722<br>mcarrillo@alz.org
**Co-Chair**<br>Bruce E. Hillner, M.D.<br>Virginia Commonwealth University<br>(804) 239-0768<br>bruce.hillner@vcuhealth.org	**Co-Chair**<br>Barry A. Siegel, M.D.<br>Washington University<br>(314) 362-2809<br>siegelb@wustl.edu
**Co-Chair**<br>Peggye Dilworth-Anderson, Ph.D.<br>University of North Carolina – Chapel Hill<br>(919) 843-2494<br>dilworth@email.unc.edu	**Co-Chair**<br>Rachel A. Whitmer, Ph.D.<br>University of California – Davis<br>530-754-0887<br>rawhitmer@ucdavis.edu
**Co-Chair**<br>Consuelo Wilkins, M.D.<br>Vanderbilt University<br>(615) 953-2820<br>Consuelo.H.Wilkins@Meharry-Vanderbilt.org	**Co-Chair**<br>Sid O'Bryant, Ph.D.<br>University of North Texas<br>(817) 735-2963<br>Sid.OBryant@unthsc.edu
**Co-Chair**<br>Robert Rissman, Ph.D.<br>University of Southern California<br>University of California, San Diego<br>(858) 246-0140<br>rr_863@usc.edu	**Study Statistician**<br>Constantine Gatsonis, Ph.D.<br>Center for Statistical Sciences<br>Brown University<br>(401) 863-2175<br>gatsonis@stat.brown.edu
New IDEAS Study Operations Office<br>American College of Radiology<br>50 South 16th Street, Suite 2800<br>Philadelphia, PA 19103	Version Date: January 25, 2023<br>Clinicaltrials.gov ID: NCT04426539

This protocol was designed and developed by the American College of Radiology and the Alzheimer's Association. It is intended to be used only in conjunction with institution-specific IRB approval for study entry. No other use or reproduction is authorized by ACR, nor does ACR assume any responsibility for unauthorized use of this protocol.

Table of Contents

List of Abbreviations and Acronyms **4**
SCHEMA **5**
STUDY OBJECTIVES/SPECIFIC AIMS **6**
1.0 ABSTRACT **7**
2.0 INTRODUCTION/BACKGROUND **9**
2.1 IDEAS 9
2.2 New IDEAS 11
2.2.1 Rationale for evaluating 12-month outcomes for amyloid positive versus amyloid negative patients 12
2.2.2 Rationale for investigating the effect of amyloid PET in ethnoracial minorities. 14
2.2.3 Rationale for investigating the effect of amyloid PET in typical, atypical, and early onset dementia patients 17
2.2.4 Rationale for collecting plasma and DNA samples in a biorepository 18
3.0 OBJECTIVES **18**
3.1 Aim 1 18
3.1.1 Primary Objective 18
3.1.2 Secondary Objectives 19
3.1.3 Exploratory Objective 19
3.2 Aim 2 19
3.2.1 Primary Objective 19
3.2.2 Secondary Objectives 20
3.2.3 Exploratory Objectives 20
3.3 Aim 3 21
3.3.1 Primary Objective 21
3.3.2 Secondary Objectives 21
3.3.3 Exploratory Objective 22
3.4 Additional Objectives 22
3.4.1 Objective A: Biorepository 22
3.4.2 Objective B: PET Image Collection 23
3.5 Approach and Expertise 23
4.0 PATIENT ELIGIBILITY CRITERIA AND REGISTRATION **25**

4.1 Inclusion Criteria 25
4.1.1 NIA-AA Research Framework for MCI and Dementia 25
4.1.2 Classification of Clinically Typical or Clinically Atypical Alzheimer's Disease 26
4.2 Exclusion Criteria 27
4.3 Referring Physician (Dementia Specialist) Practice Selection 28
4.4 Dementia Specialist and Research Staff Eligibility 28
4.5 PET Facility Eligibility and Registration Requirements 29
4.6 Recruitment and Screening 29
4.6.1 Screening and Responsibilities 29
4.6.2 Informed Consent 30
4.6.3 Participant Registration in the Study 30
4.7 Inclusion of Women and Minorities 31
4.7.1 Strategies for Minority Recruitment 31
4.8 Monitoring Enrollment and Retention 34
5.0 STUDY DESIGN AND CALENDAR 34
5.1 VISIT 1: Clinical Assessment—Pre-PET Visit Reporting Intended Management 36
5.1.1 Socio-demographic electronic Case Report Form (eCRF) 36
5.1.2 Pre-PET electronic Case Report Form (eCRF) 36
5.2 VISIT 2: Amyloid PET Imaging Visit 37
5.2.1 Imaging Acquisition 37
5.2.2 Subjectivity of Amyloid PET Interpretation 38
5.2.3 Digital Image Submission Using TRIAD™ to ACR PET Imaging Archive 38
5.3 BioSpecimen Collection 38
5.3.1 Saliva Collection 38
5.3.2 Blood Collection 39
5.4 Amyloid PET Disclosure 39
5.5 VISIT 3: 90 Days (± 30 Days) After Amyloid PET Scan (Visit 2) 40
5.6 Case Completion and Reimbursement 40
5.7 CMS Claims Data Collection 41
5.8 Central Institutional Review Board Approval for Study Participation 41
5.9 New IDEAS Study and CMS-Suggested Design Specifications 41
5.10 New IDEAS Study Website and Data Collection 45
6.0 CONFLICT OF INTEREST 45

7.0 DATA PROTECTION, ACCESS, AND PUBLICATION 45
7.1 Data Access and Publication Policy 45
7.2 Data Privacy 46
7.3 Record Retention 47
8.0 STATISTICAL CONSIDERATIONS 47
8.1 Aim 1 47
8.1.1 Primary Objective 47
8.1.2 Secondary Objectives 48
8.1.3 Exploratory Objective 48
8.2 Aim 2 49
8.2.1 Primary Objective 49
8.2.2 Secondary Objectives 50
8.2.3 Exploratory Objectives 51
8.3 Aim 3 52
8.3.1 Primary Objectives 52
8.3.2 Secondary Objectives 53
8.3.3 Exploratory Objective 54
9.0 REFERENCES 55
Appendix I: Saliva and Blood Collection 60
Appendix II: New IDEAS Oversight and Organization Structure 63

List of Abbreviations and Acronyms

AA	Alzheimer's Association
Aβ	Beta-Amyloid
ACR	American College of Radiology
AD	Alzheimer's Disease
ADNI	Alzheimer's Disease Neuroimaging Initiative
ApoE	Apolipoprotein E
ApoE4	Apolipoprotein E4
ATRI	Alzheimer's Therapeutic Research Institute
AUC	Appropriate Use Criteria
CBC	Complete Blood Count
CED	Coverage with Evidence Development
CMS	Centers for Medicare & Medicaid Services
CRI	Center for Research and Innovation
CRF	Case Report Form
CT	Computed Tomography
ED	Emergency Department
eCRF	Electronic Case Report Form
EC	Ethics Committee
FDA	Food and Drug Administration
IAC	Intersocietal Accreditation Commission
IRB	Institutional Review Board
MCI	Mild Cognitive Impairment
MMSE	Mini Mental State Examination
MRI	Magnetic Resonance Imaging
NAPA	National Alzheimer's Project Act
NCD	National Coverage Decision
NIA-AA	National Institute on Aging – Alzheimer's Association
NIH	National Institutes of Health
NOPR	National Oncologic PET Registry
PET	Positron Emission Tomography
PET/CT	Positron Emission Tomography/Computed Tomography
PET/MRI	Positron Emission Tomography/Magnetic Resonance Imaging
SNMMI	Society of Nuclear Medicine and Molecular Imaging
USC	University of Southern California

SCHEMA

Time point	Participant Events	Data Collection
T1 (Visit 1)	**Clinical Visit with dementia specialist:** Participant screened for eligibility, consented and referred for amyloid PET scan.	**Registrar:** Submit Case Registration Form and Socio-demographics Form.
T2		**Dementia specialist:** Submit Pre-PET Form within 7 days of Case Registration.
T3 (Visit 2)	**Amyloid PET Scan** Scan cannot begin until AFTER T2 – receipt of Pre-PET form<br><br>Scan must be completed within 60 days of T2	**Facility Staff:** Submit Scan Completion Form within 7 days of the day of scan
		**Radiologist/Nuc. Med. Phys.:** Dictate report and complete PET Assessment Form within 7 days of the scan. Note: same physician must interpret scan and complete assessment.
T4	Time point at which dementia specialist discloses results of the amyloid PET scan. This is a standard of care appointment. Adjustments to patient management based on PET results recommended if appropriate.<br><br>Saliva collection kit mailed to participant's address. Blood collection kit mailed, if applicable	No study data collection is associated with this time point
T5 (Visit 3)	**Clinical visit with Dementia specialist:** Clinical visit to assess participant's status, assess whether changes to management made at T4 have been implemented (i.e. adherence to and tolerance of treatment), and to gather data for Post-PET form.<br><br>This visit should take place approximately 90 days after PET scan, but no less than 60 days and no more than 120 days.	**Dementia specialist:** Submit Post-PET form. Post-PET form requests status update for each management item that had been planned before-PET and any new items added since the PET scan.

STUDY OBJECTIVES/SPECIFIC AIMS

The over-arching goal of the New IDEAS Study is to evaluate the utility of beta-amyloid PET to "exclude Alzheimer's disease (AD) in narrowly defined and clinically difficult differential diagnoses" in a broad and diverse population of Medicare beneficiaries with cognitive impairment, as described in [CMS' Decision Memo for Beta Amyloid Positron Emission Tomography in Dementia and Neurodegenerative Disease (CAG-00431N), Sept. 27, 2013.]

New IDEAS will align with the Center for Medicare and Medicaid Services' (CMS) National Coverage Determination (NCD) stated clinical objectives to (1) "*develop better treatment and prevention strategies for AD*" patients, those who are most likely to develop AD, and for those for whom Alzheimer's disease (AD) has been excluded. The study will also identify subpopulations at risk for AD, and (2) "*resolve clinically difficult differential diagnoses*." We will follow and compare two cohorts of patients: those for whom a beta amyloid PET scan is consistent with underlying AD as causing or contributing to cognitive impairment, and those for whom the scan has ruled out AD, to determine if beta-amyloid PET imaging has affected patient health outcomes, including short term outcomes related to changes in management and longer term dementia outcomes. In addition, New IDEAS will concentrate on the recruitment of Blacks/African Americans and Latinx/Hispanics, as well as Medicare beneficiaries with early-onset (age < 65) cognitive impairment. New IDEAS will evaluate and compare changes in management and CMS claims-derived health outcomes in amyloid PET-positive versus amyloid PET-negative individuals in a diverse sample of patients with mild cognitive impairment (MCI) and dementia. The study adds saliva collection for genotyping of ApoE, an important risk factor for brain amyloidosis, in all participants. Additional objectives of the study are to establish a biorepository of plasma and DNA samples and to archive PET images from study participants who consent to these optional elements.

The New IDEAS protocol addresses the requirements of the Coverage with Evidence Development (CED) provisions of the National Coverage Decision (NCD) in an expanded profile of African-American and Latinx/Hispanic participants. The vast majority of participants in studies on amyloid PET and its relationship to clinical and genetic phenotypes have been White/Caucasian. Data from more diverse populations are needed, particularly in Blacks/African Americans and Latinx/Hispanics, who may be at higher risk for dementia. New IDEAS seeks to answer CED Criterion/Question #2:

"Are there specific subpopulations, patient characteristics or differential diagnoses that are predictive of improved health outcomes in patients whose management is guided by the PET Aß imaging?"

AIM 1: To compare 12-month claims-derived health outcomes in amyloid PET-positive versus amyloid PET-negative individuals presenting with MCI and dementia in the entire study cohort of diverse Medicare beneficiaries.

AIM 2: To describe the association of amyloid PET findings with changes in patient management and 12–month claims-derived health outcomes among Blacks/African Americans, Latinx/Hispanics and Whites/Caucasians presenting with MCI and dementia.

AIM 3: To describe the association of amyloid PET findings with changes in management and 12-month claims-derived health outcomes in individuals presenting with typical (progressive amnestic) versus atypical clinical presentations of MCI and AD dementia.

ADDITIONAL OBJECTIVES

There are two additional objectives described below that are outside the scope of the NCD. Separate funding will be sought for them.

Objective A: Biorepository

The objective of the Biorepository is to collect and bank plasma and DNA from this unique, practice-based sample of cognitively impaired patients. These samples will serve as a resource to the field, enabling the testing and validation of emerging genetic and blood biomarkers that predict brain amyloidosis, and may, in the future, enable a multi-tiered, cost-effective approach for determining which select patients should undergo amyloid PET. Saliva will be collected from all study participants for DNA analysis. After analysis, saliva samples will be destroyed. Plasma and DNA will be extracted from whole blood samples, and stored for future research, from those participants who specifically consent to blood collection.

Objective B: PET Image Collection

Amyloid PET images of consenting patients will be collected, archived, and serve as a resource for future research. As in the first IDEAS Study, participants will have the option to "opt out" of this part of the study during the consent process.

ELIGIBILITY (see Section 4.0 for full inclusion/exclusion criteria)

Participants must be Medicare beneficiaries with Medicare as their primary health insurance, be referred by qualified dementia specialists, and meet the following inclusion criteria:

- 2018 NIA-AA criteria for MCI or dementia.
- Meet criteria for "clinically typical" or "clinically atypical" presentation of Alzheimer's disease.

1.0 ABSTRACT

This protocol for a prospective human research study will be conducted according to United States and International Conference on Harmonization Good Clinical Practice Guidelines, applicable government regulations (e.g., Title 45, Part 46 Code of Federal Regulations), and the American College of Radiology (ACR) research policies and procedures.

New IDEAS is an observational, open-label, longitudinal cohort study designed to address the requirements of the CED provisions of the NCD on beta-amyloid PET. Building on the initial Imaging Dementia-Evidence for Amyloid Scanning (IDEAS) study, New IDEAS will evaluate the association between amyloid PET and patient-centered outcomes in an expanded and more

ethnoracially and clinically diverse group of Medicare participants presenting with cognitive impairment.

A total of 7,000 Medicare beneficiaries meeting the study's eligibility criteria will be consented and enrolled over 30 months at sites throughout the United States. To ensure diversity, the study will enroll at least 2,000 Blacks/African Americans, at least 2,000 Latinx/Hispanics, and up to 3,000 additional participants from other racial and ethnic backgrounds. Based on disease stage prior to PET, all participants will be classified as having MCI or dementia as their disease stage. Based on their clinical presentation prior to PET, all participants will be classified as having "typical" (i.e. progressive amnestic) or "atypical" clinical presentations of AD as the potential *cause* of dementia or MCI.

Dementia specialists will team with PET facilities that have trained radiologists/nuclear medicine physicians and access to perform amyloid PET. All participating physicians and study staff will complete comprehensive training to ensure adherence of data requirements and study timelines. Amyloid PET will be performed and interpreted at each facility with results provided to the ordering dementia specialist for support in further decision making. The dementia specialists will record their diagnosis and *intended* management plan based on the current clinical and diagnostic information, and assuming no future access to amyloid PET at the "Pre-PET visit." This represents a "thought experiment" documenting the management plan that would be recommended by the specialist if the participant were not enrolling in New IDEAS and thus had no access to amyloid PET. PET results will be disclosed to patients and any consequent changes in management (if any) will be recommended at the "PET disclosure visit." Patients will return 90 ± 30 days following PET for an in person "Post-PET visit." At this final visit, the dementia specialists will record the diagnosis and *implemented* management plan, incorporating amyloid PET into clinical decision making. Medicare claims data will be collected directly from CMS for 12 months prior to the PET imaging and 12 months after the PET imaging, for each participant.

Aim 1 utilizes Medicare claims data to compare 12-month claims-derived outcomes in amyloid PET-positive versus amyloid PET-negative individuals with MCI and dementia across the entire cohort. Aims 2 and 3 investigate these associations in sub-groups of study participants based on self-identified race and ethnicity (Aim 2) and clinical presentation (Aim 3). Aims 2 and 3 additionally evaluate changes in management between the pre- and post-PET visits in the relevant sub-groups, to test whether benefits in health outcomes are mediated by changes in clinical management. Our over-arching hypothesis, supported by preliminary data from the first IDEAS study, is that amyloid PET results will be associated with changes in clinical management, which in turn will translate into improved health outcomes in patients with amyloid PET-positive scans in comparison with patients with amyloid PET-negative scans. We further hypothesize that these effects will be seen across patients of different ethnoracial backgrounds, clinical presentations and disease stages (MCI and dementia).

Optional components of the study include the collection and archival of participant's amyloid PET images and blood plasma. These repositories will serve as a resource to the field, enabling the testing and validation of emerging genetic and blood biomarkers. Separate consent will be obtained for participation in these components.

New IDEAS will be led by the team of co-investigators that successfully conducted the first IDEAS study, joined by new experts in recruitment of diverse populations and fluid-based biomarkers. The Study Chair will remain Dr. Gil Rabinovici, Distinguished Professor of Neurology, Radiology and Biomedical Imaging at the University of California San Francisco. Co-Chairs will remain Drs. Maria Carrillo, Chief Science Officer, Alzheimer's Association; Barry Siegel, Professor of Radiology and Medicine, Washington University School of Medicine; Bruce Hillner, Professor, Internal Medicine, Virginia Commonwealth University, Medical College of Virginia School of Medicine; and Rachel Whitmer, Professor and Chief of Epidemiology, UC Davis. Study statistician will remain Dr. Constantine Gatsonis, Professor of Biostatistics, Department of Biostatistics at Brown University. Additional co-chairs include Dr. Consuelo H. Wilkins, Professor and Vice President for Health Equity, Vanderbilt University Medical Center; Dr. Peggye Dilworth-Anderson, Professor of Health Policy and Management, University of North Carolina, Chapel Hill; Dr. Sid O'Bryant, Professor and Executive Director of the Institute for Translational Research, University of North Texas; and Dr. Robert Rissman, Associate Professor of Neurosciences at the University of California, San Diego (UCSD) and Adjunct Professor of Neurology at University of Southern California (USC).

2.0 INTRODUCTION/BACKGROUND

2.1 IDEAS

The original Imaging Dementia—Evidence for Amyloid Scanning (IDEAS) Study evaluated the impact of beta-amyloid (Aβ) PET on patient-oriented outcomes in Medicare beneficiaries aged ≥ 65 with Mild Cognitive Impairment (MCI) or atypical dementia meeting Appropriate Use Criteria (AUC) for amyloid imaging. The original IDEAS study opened in February 2016 and concluded recruitment in January 2018. The practice-based referral network that was established for IDEAS is unprecedented in dementia research in its size and scope. The study engaged 946 dementia experts, who recruited Medicare beneficiaries from 595 dementia clinics and referred the subjects for imaging at 343 PET facilities across the United States. There were 16,008 participants registered for Aim 1 of the study, of which 13,444 had completed an amyloid PET scan by January 2018. The median age of study participants in the Aim 1 analysis was 75 years; 50.9% were female and 88% were Caucasian. The leading suspected etiology for symptoms was AD in 76.9% of the participants, with 44.3% of participants prescribed medications for AD at enrollment. For these patients, amyloid imaging was positive in 55.3% categorized as MCI and 70.1% categorized as dementia.

The final Aim 1 data analysis on 11,409 participants demonstrated that there was a change between the pre-PET and post-PET composite management endpoint in 60.2% of patients with MCI and 63.5% of patients with dementia, significantly higher than the 30% rate of change hypothesized in the study protocol for each sub-population ($p<0.001$). [1] The primary etiologic diagnosis changed in 35.6% of study participants (25.1% from AD to a non-AD diagnosis, and 10.5% from non-AD to AD). In patients with a positive amyloid scan, the rate of AD diagnosis increased from 80.3% pre-PET to 95.5% post-PET. An even larger effect was seen in patients with a negative amyloid scan, as the rate of AD diagnosis decreased from 71.5% pre-PET to 10.2% post-PET. Having access to the results of PET imaging also had a substantial impact on the use of AD medications. Specifically, for patients diagnosed with dementia with a positive amyloid PET scan, the use of AD drugs increased from 63.2% pre-PET to 91.2% post-PET.

Longitudinal follow-up and analysis of the cohort for Aim 2 (claims-based medical outcomes) will be completed in early 2020, with publication anticipated in mid-2020.

Given the lack of neuropathological validation in IDEAS, one question that has arisen is whether amyloid PET status accurately predicts underlying AD pathology, thus justifying changes in diagnosis and management based on PET results. The neuropathological diagnosis of AD is based on the distribution and burden of amyloid plaques and tau-containing neurofibrillary tangles.[2] Based on extensive clinicopathological studies, intermediate-to-high AD neuropathological changes (ADNC) are considered clinically significant. Moderate-to-Frequent density of neuritic plaques (as measured by the Consortium to Establish a Registry for AD (CERAD) criteria[3] are required to diagnose this level of ADNC. Absent-to-low neuritic plaque density is therefore considered inconsistent with clinically significant AD neuropathology.

The FDA approved amyloid PET tracers based on pivotal studies that demonstrated that blinded visual reads of *ante mortem* PET showed 88%-98% sensitivity and 80%-95% specificity in identifying CERAD moderate-frequent neuritic amyloid plaques detected at autopsy. [4,5,6] A recent multi-site study from UCSF, Mayo Clinic, University of Pittsburgh and the Australian Imaging Biomarker and Lifestyle studies examined PET-to-autopsy correlations in 179 research participants, most with MCI or mild dementia (mean age 73 ± 12 years; mean PET-to-autopsy 3.1 ± 2.1 years). Amyloid PET showed 84% sensitivity and 88% specificity in distinguishing none-absent from intermediate-high AD Neuropathological Changes (positive/negative predictive values 92%/76% respectively).[7]

A recent study from UCSF compared visual reads of amyloid versus FDG-PET head-to-head in 101 consecutive cognitively impaired research participants with autopsy-confirmed diagnoses (mean age: 67, PET-to-autopsy interval: 4.4 years). Compared to FDG-PET, PIB-PET showed higher sensitivity for detecting AD pathology (96% [89-100%] vs 80% [68-92%] for FDG, p=0.02) and higher negative predictive value (96% [91-100%] vs 84% [74-93%], p=0.01), with equivalent specificity (86% [76-95%] vs 84% [74-93%], p=0.80).[8]

These data all support the high accuracy of amyloid PET in detecting clinically meaningful AD neuropathology, and support the PET-driven changes in diagnosis and management observed in the first IDEAS study.[1]

Anticipating the opportunity to perform more research on this unique cohort, we prospectively asked IDEAS participants for additional consent to archive PET images (95.1% affirmative response) and to be contacted about additional research (80.7% affirmative response). IDEAS "add-on" studies have received extramural funding to enrich the study by incorporating longitudinal computer-based cognitive and functional measures (Brain Health Registry, BHR-IDEAS), genetics (Amyloid Neuroimaging and Genetics Initiative, ANGI), amyloid risk screening (Plasma Test for Amyloid Risk Screening, PARIS), and caregiver outcomes (Caregivers Reactions and Experience, CARE-IDEAS) in a sub-set of IDEAS participants.

Thus, in addition to pursuing aims relevant to the reimbursement of amyloid PET, the IDEAS Study has established an unparalleled practice-based network that can be utilized to address novel questions in Alzheimer's disease (AD) research in clinically relevant populations, pursuant

to the goals of the National Alzheimer's Project Act (NAPA), Public Law No: 111-375 (01/04/2011). However, enrollment of IDEAS participants in add-on studies has proven challenging after completion of their participation in the primary study, primarily due to logistical problems related to post-hoc recruiting. Thus, the overall proportion of IDEAS participants recruited to add-on studies has been 5% or less.

Overall, the IDEAS Study has demonstrated that a large-scale, "real world" study in dementia practice is feasible, that participants are motivated to engage in research, and that the IDEAS network can be leveraged to address both CED-relevant questions as well as additional research questions that are critical to achieve the goals put forth in NAPA.

2.2 New IDEAS

New IDEAS is a novel CED study that will address additional gaps in knowledge that are highly relevant to improve precision in future coverage decisions and implementation of amyloid PET in clinical practice. A major limitation of the IDEAS cohort was lack of racial and ethnic diversity, with 88% of participants identified as Non-Hispanic White/Caucasian. This likely reflects discrepancies in minority access to specialist care, as well as the need for tailored approaches to successfully recruit these populations into research studies. In 35 years, one-third of the U.S. population over the age of 85 will be Latinx/Hispanic, Black/African-American or other ethnoracial minority; if trends persist, >50% of these individuals will suffer from dementia.

Numerous studies have shown that in comparison to Whites, Blacks/African-Americans have a 50-100% higher rate of dementia, Latinx/Hispanics a similar or higher rate depending on geography (West Coast versus East Coast), and Asian-Americans a markedly lower rate.[9] Little is known about amyloid PET or blood biomarker (genetic or plasma) performance in these under-represented minorities, even though altogether these groups will comprise the majority of the elderly population by 2050. In the first IDEAS study, rates of amyloid PET-positivity were significantly lower in minority populations versus Whites with MCI or dementia (see section 2.2.2 below), consistent with neuropathological studies that demonstrate a relatively greater contribution of cerebrovascular disease to cognitive impairment, and relatively lower contribution of AD, in Latinx/Hispanics and Blacks/African-Americans compared to non-Hispanic Whites. [10] These data support the need for further study of racial and ethnic differences in the relationship between amyloid PET and cognitive decline. A comprehensive, multi-disciplinary approach is needed to effectively recruit these under-served populations into clinical and biomarker research.[11,12]

Since the publication of the initial Appropriate Use Criteria for amyloid PET, new data from multiple studies show high rates of negative amyloid PET (35%-50%) in clinically "typical" (i.e., memory-predominant) MCI and AD dementia, particularly in ApoE4-negative individuals. Autopsy studies suggest that these patients have an alternative neuropathological cause of cognitive impairment and have been misdiagnosed with AD,[13,14] supporting an important clinical role for amyloid PET in these patients.[15,16] These findings run contrary to the assumption in the AUC that patients with "typical" AD would not require amyloid PET for diagnostic purposes. Patients with typical clinical presentations of AD were not included in the original IDEAS Study, and ApoE genotyping was not performed in the study.

2.2.1 Rationale for evaluating 12-month outcomes for amyloid positive versus amyloid negative patients

In a pre-specified secondary outcome in the first IDEAS study, we examined whether 12-month rates of hospitalizations and ED visits differed in amyloid PET-positive versus amyloid PET-negative participants. This analysis combined CMS claims data and IDEAS amyloid PET information status. Scan results were available for 11,213 participants for whom CMS records were identified. Table 1 presents the proportion of IDEAS participants who had a hospitalization or an ED visit within 12 months from their scan. The analysis indicated that a positive scan was associated with a lower probability of 12-month hospitalizations in both the MCI and dementia subgroups. A similar association was observed for 12-month ED visits for Dementia and a weaker one for the MCI cohort. A more detailed analysis of the same dataset using logistic regression led to similar conclusions. In addition to amyloid PET result, impairment status, and their interaction, the model included covariates representing sociodemographic characteristics and comorbid conditions.

Table 1: 12-month hospitalization and ED visit rates vs. amyloid PET results in IDEAS study

	**Hospitalization within 12 months**			**ED visit within 12 months**		
	Amyloid PET positive	Amyloid PET negative	OR[1] , (95% CI)[2]	Amyloid PET positive	Amyloid PET negative	OR, (95%CI)
MCI[3]	0.19 (701/3750)	0.23 (662/2897)	0.78 (0.70, 0.88)	0.40 (1515/3750)	0.41 (1178/2897)	0.99 (0.89,1.10)
Dementia	0.24 (743/3098)	0.31 (402/1312)	0.77 (0.66, 0.89)	0.47 (1460/3098)	0.53 (695/1312)	0.85 (0.75, 0.96)
Total	0.21 (1444/6848)	0.25 (1064/4209)	0.78 (0.71,0.87)	0.43 (2975/6848)	0.45 (1873/4209)	0.95 (0.86, 1.03)

[1] OR = Odds Ratio: measure of association between the diagnosis and the rate of hospitalization or ED visits.
[2] CI = Confidence Interval (95%)
[3] MCI = Mild Cognitive Impairment

Complimentary analyses are underway to attempt to better understand the mechanisms underlying these observations. As demonstrated in Kaiser Northern California[17] and other care systems[18], a targeted and multi-disciplinary care plan for cognitively impaired patients can reduce hospitalizations and ED visits by improving safety planning, enhancing caregiver knowledge and confidence and providing continuous support to alleviate acute care needs. It is possible that a positive amyloid scan, providing support for a definitive diagnosis of AD, enhances the likelihood of developing a comprehensive care plan, translating into improved health outcomes. Conversely, a negative scan may perpetuate diagnostic uncertainty, and thus fail to activate downstream care plans. An alternative explanation is that conditions that lead to MCI or dementia in the absence of amyloid (e.g., vascular or parkinsonian dementias) may also be associated with increased risk of hospitalizations compared to AD. However, this explanation is less likely given that the association between amyloid PET result and hospitalizations is maintained in a logistic regression model that controls for co-morbid conditions and previous utilization. Overall, the results described above represent to our knowledge the first reported associations between amyloid PET results and health outcomes in cognitively impaired

individuals. An important caveat is that the population studied in the first IDEAS study was primarily White, and all patients were over age 65 and met AUC, i.e. had atypical clinical presentations. Therefore, these results cannot be generalized to more ethnoracially diverse populations, or to patients with early age-of-onset (under age 65) or typical clinical presentations of AD.

In New IDEAS, we seek to build on and replicate these findings in a more diverse population than was included in the first IDEAS study, emphasizing recruitment of ethnoracially diverse patients, and including patients with typical as well as atypical clinical presentations of MCI and AD, as well as patients with early age-of-onset cognitive impairment. Analogous to IDEAS, we have selected as our primary outcome for Aim 1 12-month hospitalization rates. These represent high-stakes, resource-intensive, patient-oriented outcomes. Secondary outcomes will include 12-month ED visits; 12-month hospitalization due to ambulatory-sensitive conditions; 12-month ED visit due to ambulatory-sensitive conditions; and overall resource utilization during the 12-month period from baseline testing. Exploratory analyses will assess the association of amyloid PET result with 12-month hospitalizations and 12-month ED visits in the presence of information about change of management following the amyloid PET scan. This aim is directly responsive to CED Criteria #2: "Are there specific subpopulations, patient characteristics or differential diagnoses that are predictive of improved health outcomes in patients whose management is guided by the PET Aβ imaging?"

Patients with dementia have a two- to three-fold increased risk for hospitalizations and ED visits compared to elderly individuals without dementia.[19,20] Patients with MCI or dementia may have difficulty recognizing dangerous symptoms, effectively managing their self-care of chronic conditions, and communicating symptoms and changes in their health status. Patients with dementia are significantly more likely to experience delirium, agitation, iatrogenic complications, decreased functional ability, organ dysfunction, severe sepsis, death, longer hospital stays, and transition from hospitalization to institutional care.[21-23] Hospitalization is also associated with a substantially increased risk of death and shorter survival time for patients with dementia compared to those without dementia.[58] Some racial/ethnic minority groups are significantly *more* likely to be hospitalized or have an emergency room visit. As seen in Table 2, in an analysis of almost 60,000 patients with dementia,[12] African-Americans were 30% more likely to have an ED visit or hospitalization within the first 3 months after diagnosis.
Our underlying hypothesis, informed by the preliminary data in Table 1, is that patients with positive amyloid PET scans will have lower rates of hospitalizations and ED visits compared to patients with negative scans. We further hypothesize that this effect will be mediated by changes in patient management, supporting the premise that the care plan triggered by a definitive diagnosis is responsible for improved health outcomes. In our analyses, we will examine the association between amyloid PET results and health outcomes in different sub-populations, including patients with MCI vs. dementia, typical vs. atypical clinical presentations, and patients from different racial and ethnic backgrounds. Overall, this study will assess the prognostic utility of amyloid PET in a broad and diverse population representative of seen in specialty memory clinics.

2.2.2 Rationale for investigating the effect of amyloid PET in ethnoracial minorities.

In 35 years, one-third of the U.S. population over the age of 85 will be Latinx/Hispanic, Black/African American or other ethnoracial minority; if trends persist, >50% of these individuals will suffer from dementia. Numerous studies have shown that in comparison to Whites, Blacks/African Americans have a 50-100% higher rate of dementia, Latinx/Hispanics have a similar or higher rate depending on geography (West Coast versus East Coast), and Asian-Americans have a markedly lower rate.[9] Risk factors for dementia, including hypertension, diabetes and low educational attainment, are more common in Blacks/African Americans and Latinx/Hispanics.[10] Redressing these ethnic racial disparities is a major goal of NAPA. Yet, the current base of scientific knowledge about amyloid PET, the primary biomarker for AD, is based primarily on cohorts or clinical trial subjects that are disproportionately non-Hispanic White.[24] To date there is a dearth of information and data on the primary AD biomarker (amyloid PET) in Latinx/Hispanics and Non-Whites. This is a major public health inequity that New IDEAS can directly redress.

For example, participants in the Alzheimer's Disease Neuroimaging Initiative, the largest, multi-site longitudinal observational study of AD, are 89% White; less than 5% of AD clinical trials participants are African American and fewer than 3% are Latinx.[15,16] *This inequity is highly troubling given that currently we know the least about the accuracy and utility of AD biomarkers in the very groups that are at highest risk.* Given the dearth of data, it is unknown if these differences in rates reflect differences in burden of amyloid pathology, clinical management, or contribution of known genetic risk factors for AD such as ApoE4 genotype.[25,26] This information is greatly needed to advance our understanding of AD in minority populations. Recruitment in IDEAS unfortunately mirrored prior clinical trials, with approximately 88% of participants Non-Hispanic White/Caucasian, 3.8% Black/African American, and 4.0% Hispanic/Latinx.

Disparities in rates of MCI, AD dementia, and non-AD dementia are less clear than often assumed, and some studies of cognitive decline do not find corresponding disparities. While prevalence studies generally indicate that Blacks/African Americans have double the risk of dementia as Whites/Caucasians, incidence studies have reported no elevation.[27,28] Incidence [24,29-31] and prevalence [24,29] studies in Latinx/ Hispanics report rates of dementia that are between 1.5 and 3 times higher than those of Whites/Caucasians. However, these studies usually apply to East Coast and Caribbean Hispanics, whereas studies from California of Hispanics of Mexican descent do not show elevated risk.[32]

These differences in ethnoracial patterns of prevalence of dementia versus ethnoracial patterns of cognitive decline suggest that the trajectory and, possibly, the pathology behind dementia is not uniform across all race groups; indeed there is still much to be learned regarding the basic epidemiology of dementia and AD in Non-Whites. Analysis of global studies finds that dementia rates in Latin America may be lower than those in the U.S.[33]

Figure 1 shows the 14-year cumulative incidence of dementia in 274,543 Kaiser Permanente/Northern California members over the age of 64 (n = 18,778 African-American, n = 4543 American Indian/Alaska Native,[9] n = 21,000 Latinx, n = 440 Pacific Islander, n = 206,490 white, n = 23,032 Asian-Americans). African Americans had the highest cumulative incidence of dementia, while risk was similar in Latinx and non-Hispanic whites. Cumulative 25-

year risk at age 65 showed similar trends, with prevalence reported at: 38% African Americans, 35% Native Americans, 32% Latinx, 25% Pacific Islanders, 30% White, and 28% Asian Americans. The analysis of Kaiser Permanente/Northern California patient data[9] showed marked differences in rates of hospitalization after diagnosis of dementia by ethnoracial group:

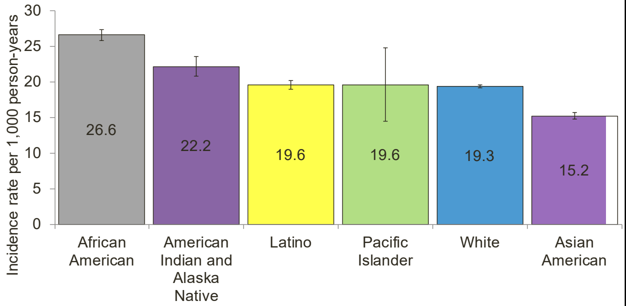

Figure 1. 14-year Incidence Rates of All Cause Dementia in 274,000 Members of Kaiser Permanente Northern California.Figure adapted from Mayeda et al., Alzheimer's & Dementia 2017.

- Mean age at dementia diagnosis was 83.1 years
- More than half of dementia patients had an emergency room (ER) visit within 12 months of diagnosis (Figure 1)
- More than a third of dementia patients had a hospitalization within 12 months of diagnosis (Figure 1)
- Asian Americans were least likely to have an ER visit or hospitalization; African Americans and American Indians/Alaska Natives were most likely (Table 2). Compared with Asian Americans, African Americans and American Indians/Alaska Natives were ~30% more likely to have an ER visit at all time points and ~25% more likely to have a hospitalization at all time points (Table 2).

Table 2: Logistic Regression Models of Ethnicity/Race and Risk of ER Visits and Hospitalizations*

	**3 months**	**6 months**	**1 year**
**Emergency Room Visits**			
	OR[1] (95% CI[2])	OR (95% CI)	OR (95% CI)
Black	1.28 (1.16-1.40)	1.30 (1.19-1.42)	1.31 (1.20-1.42)
Latinx	1.08 (0.98-1.19)	1.14 (1.04-1.25)	1.12 (1.02-1.22)
American Indian/Alaska Native	1.32 (1.15-1.52)	1.39 (1.22-1.58)	1.41 (1.24-1.61)
White	1.11 (1.03-1.19)	1.16 (1.08-1.24)	1.16 (1.09-1.24)
Asian	1.00	1.00	1.00
**Inpatient Hospitalization**			
Black	1.27 (1.14-1.41)	1.27 (1.16-1.40)	1.25 (1.14-1.37)
Latinx	0.91 (0.81-1.02)	0.97 (0.88-1.07)	1.01 (0.92-1.11)
American Indian/Alaska Native	1.20 (1.02-1.40)	1.28 (1.11-1.47)	1.25 (1.09-1.43)
White	1.05 (0.97-1.15)	1.08 (1.00-1.17)	1.09 (1.02-1.17)
Asian	1.00	1.00	1.00

**Logistic regression models adjusted for age at diagnosis, gender, stroke, depression, diabetes and cardiovascular disease in 59,494 Dementia Patients (in an analysis of Kaiser Permanente/Northern California patient data)* [9]

[1] OR = Odds Ratio: measure of association between the diagnosis and the rate of hospitalization or ED visits.

[2] CI = Confidence Interval (95%)

Disparities in healthcare access based on race and ethnicity represent a major barrier to effective and equitable care. Studies have shown that Blacks/African Americans and Latinx/ Hispanics first present to medical attention at a later stage of cognitive impairment than Whites/Caucasians.[1] In IDEAS, 51.7% of African Americans and 55.8% of Latinx/Hispanics

presented to the study at the dementia stage (in contrast to the earlier MCI stage), while in Whites the rate of dementia was 37.6%, consistent with a later disease stage at clinical presentation in high-risk minorities. [1] (Table 3)

Table 3: Level of Cognitive Impairment by Race/Ethnicity in IDEAS Study[1]

	Black n=639	*Hispanic n=848*	*Asian n=328*	*White n=15,568*	*Multiracial n=31*	*Other n=33*	*Unknown n=1,103*	*Total n=18,550*
**MCI**	307<br>48%	375<br>44%	175<br>53%	9,768<br>63%	21<br>68%	15<br>45%	614<br>56%	11,275
**Dementia**	332<br>52%	473<br>56%	153<br>47%	5,800<br>37%	10<br>32%	18<br>55%	489<br>44%	7,275

Established dementia risk factors, including ApoE, have different magnitudes of effects in different ethnoracial groups. Effect modification due to ethnoracial status is well established in medicine, but little is known about ethnicity by risk factor interactions for dementia. For example, elevated systolic blood pressure increases the risk of stroke 3 times more for Blacks than Whites;[34] the effect of age on intracerebral hemorrhage is greater in middle age in Blacks and greater in late life in Whites;[35] and inflammation increases stroke risk more in Blacks than Whites.[36] It is not known if these vascular risk factors have correspondingly greater effect for dementia in Blacks than Whites. In contrast to findings with Whites, ApoE4 in Blacks is not consistently found to confer elevated AD risk or to lower age of onset.[37-40]

A recent study reports that the impact of a polygenetic dementia risk score is significantly higher for American Whites than for American Blacks.[41] There are also conflicting reports about the prevalence of AD versus other causes of dementia (e.g., cerebrovascular disease) in minority populations versus Whites. One report found more AD dementia in Whites and more vascular dementia in Blacks, [42] others report no difference in AD pathology[42-45] and an admixture study found a protective effect of African ancestry against AD pathology.[46] Heterogeneity in findings across pathology, admixture, and rates of dementia warrant the urgent need to delineate and understand AD biomarker amyloid PET in Non-Whites on a population level. New IDEAS is poised to successfully achieve this.

Community-based MRI studies indicate that the Black-White disparities in stroke extends to "silent" brain infarcts.[47] Thus, there is conflicting evidence about levels of AD pathology in Blacks, and some evidence that cerebrovascular lesions may be more prevalent in Asians, Blacks, and in Hispanics than in Whites. *To date, no large-scale studies have investigated the relative burden of amyloid pathology in a multi-ethnic study*. In IDEAS, the rate of amyloid PET positivity was higher in Whites (71.7% in dementia, 57.0% MCI) than in Blacks/African Americans (65.1% dementia, 42.2% MCI) and Latinx/Hispanics (53.7% dementia, 46.3% MCI), suggesting that non-AD causes of cognitive impairment may be more prevalent in these minority populations.

Compared to Whites, all minority groups in IDEAS were more likely to: have been diagnosed with dementia (vs MCI) prior to PET scan; have consent by proxy; have Medicare Advantage and have diabetes. Blacks/African Americans were more likely to have hypertension and live

alone, while Latinx/Hispanics and Asian/Asian Americans were less likely to live alone compared to Whites. In addition, it was more likely for Blacks/African Americans (51%) and Latinx/Hispanics (63%) to have a high-school diploma or less, compared to Whites (31%). The preliminary data from IDEAS has shown the need to further investigate the interaction of sociocultural and demographic factors with race and ethnicity in AD.

In summary, the evidence to date suggests that Blacks/African Americans and Latinx/Hispanics bear a disproportionate burden of dementia, whereas the vast majority of the literature about genetic risks and neuropathological underpinnings of dementia are derived from overwhelmingly White cohorts. Thus, New IDEAS will provide novel insights into the causes and predictors of cognitive impairment in the groups that are at highest risk and will redress this public health inequity.

2.2.3 Rationale for investigating the effect of amyloid PET in typical, atypical, and early onset dementia patients

Clinicopathological studies suggest that the clinical diagnosis of late-onset AD is only ~70% accurate compared to autopsy.[13] In the Aim 1 analysis in IDEAS, we found that only 63.8% of patients with a pre-PET diagnosis of AD were amyloid positive, versus 52.2% PET positivity in patients with a pre-PET diagnosis of a non-AD dementia, suggesting that pre-PET diagnosis did not reliably predict amyloid status in the AUC population.[1] However, discordance between amyloid PET and clinical diagnosis occurs frequently and also in patients with a “typical” (i.e., memory-predominant) clinical presentation of AD. For example, data from observational studies (e.g., the Alzheimer’s Disease Neuroimaging Initiative [ADNI]) and clinical trials suggest that ~30% of patients meeting strict NIA-AA clinical criteria for probable AD dementia, and ~50% of patients clinically diagnosed with MCI due to likely AD (i.e., amnestic MCI, single or multi-domain) have negative amyloid PET.[15,16] Autopsy studies have shown that these cases represent clinical misdiagnosis of the underlying neuropathology, as opposed to lower sensitivity of the amyloid tracer in ApoE4-negative individuals.[10,11]

ApoE genotyping is highly correlated with brain amyloidosis, and may be useful in stratifying MCI/dementia patients who are likely to have amyloid from those likely to have negative amyloid PET. Recent data from ADNI and clinical trials demonstrate that, in patients who meet NIA-AA clinical criteria for MCI due to AD or AD dementia,[48,49] presence of the apolipoprotein E ε4 risk allele is highly predictive of positive amyloid biomarkers (>90%), whereas patients who don’t carry ApoE4 show 35%-50% rates of amyloid biomarker negativity.[15,16] In the Alzheimer’s Biomarkers in Daily Practice Study, a Dutch study on the clinical utility of amyloid PET, the rate of amyloid PET positivity was 90.5% in patients with probable AD who were ApoE4 carriers, but only 58.5% in patients who fulfilled the same clinical criteria but were negative for ApoE4.[50] Therefore, we hypothesize that in ApoE4-positive patients, amyloid PET will confirm the clinical suspicion of underlying AD, and thus have little incremental benefit. In contrast, in ApoE4-negative patients, there will be a high rate of negative PET results, thus changing management, eliminating unnecessary treatments, and ultimately leading to more precise care and improved outcomes.

Though not included in the original IDEAS Study, early-onset MCI/dementia was recommended as one of the Appropriate Use Criteria for amyloid PET.[51] This is justified by studies showing

high rates of misdiagnosis and delayed diagnosis in this population, stemming in part from atypical symptoms and clinical overlap between different types of dementia (e.g., AD and frontotemporal dementia).[52] For example, in the NIH-funded Longitudinal Evaluation of Early-Onset Alzheimer's Disease (LEADS) study, a longitudinal, observational study recruiting 400 patients with MCI or mild AD dementia at 15 academic sites across the U.S. (Drs. Apostolova, Dickerson, Carrillo and Rabinovici co-PIs), the rates of amyloid PET positivity are only ~75% in patients meeting clinical criteria (unpublished data). Furthermore, the positive predictive value of an amyloid PET scan is high in this age range, because of a low prevalence of brain amyloidosis in cognitively normal subjects of this age.[25] We therefore hypothesize that amyloid PET will have a high impact on patient management and outcomes in patients with early-onset cognitive impairment. In New IDEAS, we will recruit Medicare beneficiaries under age 65. Participants will be categorized as MCI/dementia and typical/atypical based on the same criteria applied in older study participants.

2.2.4 Rationale for collecting plasma and DNA samples in a biorepository

An important recent advance that could have major implications for the implementation of amyloid PET in clinical practice is the development of genetic predictors and plasma-based tests for brain amyloidosis. Polygenetic risk scores, which integrate genetic information from multiple common risk-modifying single nucleotide polymorphisms, can increasingly predict individual AD risk, while novel plasma-based tests of Aβ fragments have shown high concordance with amyloid PET results in pilot studies. These advances foreshadow a future in which blood-based testing could be used to stratify cognitively impaired participants in order to determine who would most benefit from amyloid PET. Importantly, genomic and blood-based biomarkers could, if validated, be used to exclude the need for PET in a large number of cognitively impaired individuals, thereby providing a tremendous cost savings. An important caveat is that these biomarkers have thus far been developed and tested primarily in highly selected research populations. At the time IDEAS was designed, blood and genomic predictors of amyloidosis were not yet sufficiently developed to justify their routine collection in the study. Therefore, a new study addressing these relationships must dedicate resources towards recruiting a more racially and ethnically diverse cohort.

3.0 OBJECTIVES

3.1 Aim 1

Aim 1: To compare 12-month claims-derived health outcomes in amyloid PET-positive versus amyloid PET-negative individuals presenting with MCI and dementia in the entire study cohort of diverse Medicare beneficiaries.

3.1.1 Primary Objective

To compare the proportions of participants with a hospitalization within 12 months, between amyloid PET-positive and amyloid PET-negative participants in the study, separately for patients with MCI and dementia.

We hypothesize that positive amyloid PET will be associated with lower proportions of participants with hospitalizations over 12 months in patients with MCI and dementia.

3.1.2 Secondary Objectives

1) To compare the proportions of participants with an ED visit within 12 months, between amyloid PET-positive and amyloid PET-negative participants in the study, separately for patients with MCI and dementia.

We hypothesize that positive amyloid PET will be associated with lower proportions of participants with ED visits over 12 months in patients with MCI and dementia.

2) To compare the proportions of participants who had a preventable hospitalization (i.e., related to ambulatory care-sensitive conditions) within 12 months, between amyloid PET-positive and amyloid PET-negative participants, separately for patients with MCI and dementia.

We hypothesize that positive amyloid PET will be associated with lower proportions of participants with preventable hospitalizations over 12 months in patients with MCI and dementia.

3) To compare overall resource utilization within 12 months between amyloid PET-positive and amyloid PET-negative study participants, separately for patients with MCI and dementia.

We hypothesize that positive amyloid PET will be associated with lower overall resource utilization over 12 months in patients with MCI and dementia.

3.1.3 Exploratory Objective

To evaluate whether the association of amyloid PET result with 12-month hospitalizations is mediated by changes in each component of the composite management endpoint.

We hypothesize that differences in proportions of 12-month hospitalizations between amyloid PET-positive and amyloid PET-negative patients will be mediated by changes in the composite management endpoint, as defined in the first IDEAS study.

3.2 Aim 2

Aim 2: To describe the association of amyloid PET findings with changes in patient management and 12–month claims-derived health outcomes among Blacks/African Americans, Latinx/Hispanics and Whites/Caucasians presenting with MCI and dementia.

3.2.1 Primary Objective

In each ethnoracial subgroup and level of impairment (MCI or dementia), to estimate the rate (proportion) of change between the pre-PET care plan and the 90-day

implemented post-PET care plan, in a composite defined as change in one or more of the following items:

- AD-specific medications (cholinesterase inhibitors and memantine)
- Non-AD medications related to CNS conditions or risk factors
- Counseling about safety and future planning.

We hypothesize that, within each ethnoracial subgroup, the frequency of 90 day post-PET changes in the management composite endpoint will be ≥ 30%.

3.2.2 Secondary Objectives

1) In each ethnoracial subgroup, to compare the proportions of participants with a hospitalization or ED visit within 12 months, between amyloid PET-positive and amyloid PET-negative participants in the study.

We hypothesize that, within each ethnoracial sub-group, positive amyloid PET will be associated with lower proportions of participants with hospitalizations or ED visits over 12 months.

2) In each ethnoracial subgroup, to compare the proportions of participants with preventable hospitalizations over 12 months, between amyloid PET-positive and amyloid PET-negative participants in the study.

We hypothesize that, within each ethnoracial sub-group, positive amyloid PET will be associated with lower proportions of participants with preventable hospitalizations over 12 months.

3.2.3 Exploratory Objectives

1) To assess and compare the prevalence of amyloid PET positivity in Blacks/African-Americans and Latinx/Hispanics to Whites/Caucasians, separately for MCI and dementia.

We hypothesize that Blacks/African-Americans and Latinx/Hispanics, in comparison with Whites/Caucasians, will have a lower proportion of amyloid PET positivity at both the MCI and dementia stage. This is driven by the hypothesis that, in minority populations, non-AD processes, in particular cerebrovascular disease (with negative amyloid PET), will have a greater attributable risk for MCI and dementia, and AD (amyloid PET positive) will have a lower attributable risk compared to Whites/Caucasians. This hypothesis is supported by preliminary data from the first IDEAS Study.

2) To assess the positive and negative predictive values of ApoE4 for amyloid positivity in Blacks/African Americans and Latinx/Hispanics to Whites/Caucasians.

Based on existing literature, we further hypothesize that ApoE4 will be less predictive of amyloid PET positivity in Blacks/African Americans and Latinx/Hispanics compared to Whites/Caucasians.

3) To assess and compare the level of cognitive impairment (proportion of MCI vs. dementia) at study entry (pre-PET visit) in Blacks/African Americans and Latinx/Hispanics vs. Whites/Caucasians.

4) To assess and compare the use of AD medications at study entry (pre-PET visit) in Blacks/African Americans and Latinx/Hispanics vs. Whites/Caucasians.

We hypothesize that, because of disparities in access to specialist care, socioeconomic factors and cultural differences in recognition of dementia, Blacks/African Americans and Latinx/Hispanics will present to the study at a later stage of cognitive impairment (higher proportion of dementia vs. MCI), and show lower baseline use of AD medications at the pre-PET visit. All initial analyses will be based on self-identified race/ethnicity, but collection and future analysis of DNA may allow a re-analysis in the future based on admixture data.

3.3 Aim 3

Aim 3: To describe the association of amyloid PET findings with changes in management and 12-month claims-derived health outcomes in individuals presenting with typical (progressive amnestic) versus atypical clinical presentations of MCI and AD dementia.

3.3.1 Primary Objective

To assess and compare the proportion of change in the composite management endpoint in patients with "typical" (progressive amnestic) versus atypical clinical presentations of AD, separately for MCI and dementia.

We hypothesize that the frequency of 90-day post-PET changes in the management composite endpoint will be ≥ 30% in patients with both typical and atypical presentations of AD.

3.3.2 Secondary Objectives

1) To assess and compare the proportions of participants with a hospitalization within 12 months, between amyloid PET-positive and amyloid PET-negative participants in the study, separately for patients with typical versus atypical clinical presentations of AD.

We hypothesize that positive amyloid PET will be associated with lower proportions of participants with hospitalizations over 12 months in patients with both typical and atypical clinical presentations of AD.

2) To assess and compare rates of 12 month hospitalizations, ED visits, preventable hospitalizations and overall resource utilization between patients with typical versus atypical clinical presentations of AD.

We hypothesize that positive amyloid PET will be associated with lower proportions of participants with 12 month hospitalizations, ED visits, preventable hospitalizations and overall resource utilization.

3) To assess and compare the proportion of change in the composite management endpoint in patients with "typical" (progressive amnestic) versus atypical clinical presentations of AD, separately for MCI and dementia.

We hypothesize that amyloid PET will lead to ≥ 30% change between the pre-PET and post-PET composite management endpoint in patients with both "typical" and "atypical" clinical presentations. We further hypothesize that the proportion of change will be higher in atypical vs. typical cases.

3.3.3 Exploratory Objective

To assess the association between change in the composite management primary endpoint and the following potential predictors and interactions:

- *Age (dichotomized at age 65)*
- *Level of cognitive impairment (MCI vs. dementia)*
- *Typical vs. atypical clinical presentation*
- *ApoE4 carriers vs. non-carriers*
- *Race / Ethnicity*
- *Medical co-morbidities (documented in pre-PET form)*

We hypothesize that early age-of-onset (<65), MCI level of cognitive impairment, atypical clinical presentations, ApoE4 non-carrier genetic status, racial/ethnic minority, and increased vascular co-morbidities will all be independently associated with greater change in management, controlling for all other variables in the model.

3.4 Additional Objectives

3.4.1 Objective A: Biorepository

The objective of the Biorepository is to collect and bank plasma and DNA from a practice-based sample of cognitively impaired patients. Patients in the core study will be asked to "opt in" to the blood collection component of the study. Saliva will be collected from all study participants for DNA analysis. After analysis, saliva samples will be destroyed. Plasma and DNA will be extracted from whole blood samples, and stored for future research, from those participants who specifically consent to blood collection. These samples will allow for testing and validation of emerging genetic and plasma biomarkers for AD and associated diseases. The long-term goal is the development of a multi-tiered, cost-effective approach for determining which select patients should undergo amyloid PET. As the biorepository is outside of the scope of the CMS-supported amyloid PET scans, it will be funded independently.

3.4.2 Objective B: PET Image Collection

As in the first IDEAS Study, participants will have the option to "opt out" of having their images archived. The amyloid PET images of those who consent will be collected and archived at the ACR and will serve as a resource for future research. An imaging archive will facilitate comparisons with blood-based biomarkers by enabling measurement of amyloid burden as a continuous quantitative measure.

3.5 Approach and Expertise

The IDEAS network extends to diverse urban and rural regions in the U.S., and we believe that a targeted effort is ideally suited to address key questions regarding dementia outcomes and their relationship to amyloid PET and biomarkers in diverse populations. New IDEAS will be led by the team of co-investigators that successfully conducted the first IDEAS study, joined by new experts in recruitment of diverse populations and fluid-based biomarkers.

The Study Chair will remain Dr. Gil Rabinovici, Distinguished Professor of Neurology, Radiology and Biomedical Imaging at the University of California San Francisco. Dr. Rabinovici is a behavioral neurologist with 15 years experience conducting PET research in AD and related disorders. Co-Chairs will remain Drs. Maria Carrillo, Chief Science Officer, Alzheimer's Association; Barry Siegel, Professor of Radiology and Medicine, Washington University School of Medicine; Bruce Hillner, Professor, Internal Medicine, Virginia Commonwealth University, Medical College of Virginia School of Medicine; and Rachel Whitmer, Professor and Chief of Epidemiology, UC Davis. Study statistician will remain Dr. Constantine Gatsonis, Henry Ledyard Goddard University Professor of Biostatistics and Chair, Department of Biostatistics at Brown University, This team has worked effectively and productively since 2014 in designing and conducting the first IDEAS study. Drs. Siegel and Hillner also led the National Oncology PET Registry (NOPR) evaluating the clinical utility of FDG-PET and other nuclear medicine diagnostics in the staging and treatment of cancer.

To lead the implementation of best practices for minority recruitment and retention for New IDEAS, we have added Drs. Consuelo H. Wilkins and Peggye Dilworth-Anderson to the core study team as Co-Chairs. Dr. Wilkins is Vice President for Health Equity at Vanderbilt University Medical Center and holds faculty appointments in the departments of medicine at both Vanderbilt University Medical Center and Meharry Medical College. Dr. Wilkins is the director of the Community Engaged Research Core in the Vanderbilt Institute for Clinical and Translational Science and is widely recognized for her work in developing and implementing innovative methods of engaging patients and communities in research. She is Principal Investigator of a Patient-Centered Outcomes Research Institute (PCORI) research award focused on understanding the impact of patient engagement on research through Community Engagement Studios,[36] in which researchers receive direct input from representative groups to frame research questions, enhance study design, implement protocols, and disseminate research. Dr. Wilkins has substantial leadership experience in community and stakeholder engagement at the Clinical and Translational Science Award (CTSA) program and national levels. Prior to her current role, Dr. Wilkins was a clinical investigator in the Knight Alzheimer's Disease Research Center at Washington University and studied cognitive impairment and early AD in African Americans.[45, 53-56]

Dr. Dilworth-Anderson was added to the core study team as Co-Chair to lead the implementation of best practices to improve minority recruitment and retention. Dr. Dilworth-Anderson is Professor of Health Policy and Management at the Gillings School of Global Public Health, and Associate Director of Integrating Special Populations Unit of the North Carolina Translational and Clinical Sciences Institute, University of North Carolina, Chapel Hill. Her expertise will support inclusive recruitment and retention strategies for diverse groups in Alzheimer's disease research. Her expertise, informed by many years of research on caregiving to older adults with dementia, have provided strategies on how best to involve patients, family caregivers and family decision makers in the recruitment process.[57-60] She will also bring years of leadership experience to the team (former president of Gerontological Society of America, former member the Medical Scientific Advisory Council of the Alzheimer's Association, and current member of the Global Council on Brain Health) to help build community relations and create partnerships with community organizations to enhance recruitment efforts in the trial.

Dr. Sid O'Bryant, joining the team as Co-Chair of the study and co-leader of the Biorepository, is a global leader in the field of blood-based biomarkers associated with Alzheimer's disease. He has created and cross-validated novel blood-based diagnostic and companion diagnostic tools to advance precision medicine in AD. He also has considerable experience with minority recruitment. He is principal investigator of the ongoing HABLE Study (n=1,000 Mexican American, n=1,000 non-Hispanic whites; R01AG054073). He also was principal investigator of the EPA-funded rural cognitive aging study, Project FRONTIER, CMS-funded MIGHTY Care program and other studies that have enrolled urban and rural dwelling underserved populations (African American, Mexican American and other Latinx groups, impoverished groups). In total, he has been responsible for enrolling approximately 10,000 participants into funded clinical and research programs with approximately 80% of these participants being from underserved populations.

Dr. Robert Rissman, joining the team as study Co-Chair and co-leader of the Biorepository, has an established, NIH-funded research program focused on fluid biomarker discovery and analysis and considerable experience in large scale biobanking for neurodegenerative disease cohort studies and clinical trials. Dr. Rissman is Associate Professor of Neurosciences at the University of California, San Diego (UCSD) and Adjunct Professor of Neurology at University of Southern California (USC). He is Director of the USC Alzheimer's Clinical Trials Consortium (ACTC) Biomarker Unit and the UCSD Alzheimer's Disease Research Center (ADRC) Neuropathology and Biomarker Cores. Together with New IDEAS Co-chair, Dr. Sid O'Bryant, Dr. Rissman is principal investigator of the HABLE Study of minority individuals.

Dr. Rachel Whitmer, Professor and Chief of Epidemiology at University of California, Davis, a current IDEAS Study Co-Chair and New IDEAS Co-Chair, leads a large research program on racial disparities in dementia utilizing both electronic health record data and field studies. She is currently the principal investigator of four NIH-funded cohort studies, which focus on enrollment, and recruitment of ethnic minority groups: The KHANDLE (Kaiser Healthy Aging and Diverse Life Experience) Study of 1711 ethnically diverse elderly individuals to evaluate disparities in dementia incidence; the Kaiser STAR (Study of Healthy Aging in African Americans) in 700 African Americans over the age of 50, which will delineate cognitive aging

starting in midlife; and Life After 90, a cohort study of brain aging, dementia and pathology in 800 ethnically diverse oldest-old individuals. Dr. Whitmer is also Principal Investigator of US POINTER, the first lifestyle intervention trial to prevent cognitive decline.

4.0 PATIENT ELIGIBILITY CRITERIA AND REGISTRATION

All inclusion and exclusion criteria must be confirmed by the referring dementia specialist and/or the participant's medical records, prior to registration.

4.1 Inclusion Criteria

1. Medicare beneficiary with Medicare as primary insurance;
2. Meets clinical criteria for Mild Cognitive Impairment (MCI) or Dementia as defined by the 2018 National Institute on Aging – Alzheimer's Association Research Framework:[61] (Refer to section 4.1.1 for guidance);
3. Brain MRI and/or CT within 24 months prior to enrollment;
4. Clinical laboratory assessment (complete blood count [CBC], comprehensive metabolic panel[CMP], thyroid stimulating hormone [TSH], vitamin B12) within the 12 months prior to enrollment;
5. Able to tolerate amyloid PET required by protocol, to be performed at a participating PET facility;
6. Willing and able to provide consent. Consent may be by proxy;
7. Neuropsychiatric syndrome can be classified into "clinically typical" or "clinically atypical" categories. (Refer to section 4.1.2 for guidance)

4.1.1 NIA-AA Research Framework for MCI and Dementia

- Mild Cognitive Impairment (MCI):
  - Cognitive performance below expected range for that individual based on all available information. This may be based on clinical judgment and/or on cognitive test performance (which may or may not be based on comparison to normative data with or without adjustments for age, education, occupation, sex, etc.). Cognitive performance is usually in the impaired/abnormal range based on population norms, but this is not required as long as the performance is below the range expected for that individual.
  - In addition to evidence of cognitive impairment, evidence of decline in cognitive performance from baseline must also be present. This may be reported by the individual or by an observer (e.g., study partner) or observed by change on longitudinal cognitive testing/behavioral assessments or by a combination of these.
  - May be characterized by cognitive presentations that are not primarily amnestic.
  - Although cognitive impairment is the core clinical criteria, neurobehavioral disturbance may be a prominent feature of the clinical presentation.

  - Performs daily life activities independently, but cognitive difficulty may result in detectable but mild functional impact on the more complex activities of daily life, either self-reported or corroborated by a study partner.

- Dementia:
  - Substantial progressive cognitive impairment that affects several domains and/or neurobehavioral symptoms. May be reported by the individual or by an observer (e.g., study partner) or observed by change on longitudinal cognitive testing.
  - Cognitive impairment and/or neurobehavioral symptoms result in clearly evident functional impact on daily life. No longer fully independent/ requires assistance with daily life activities. This is the primary feature differentiating dementia from MCI.

4.1.2 Classification of Clinically Typical or Clinically Atypical Alzheimer's Disease

- The **"clinically typical"** group is intended for patients with a memory-predominant presentation of MCI and dementia in whom the clinical course and progression are highly suggestive of AD as the underlying cause. Patients in the "clinically typical" group must meet the following criteria: [51]
  - Insidious onset. Symptoms have a gradual onset over months to years, not sudden over hours or days.
  - History of worsening of cognition by report or observation.
  - The initial and most prominent cognitive deficits are impairment in episodic memory (i.e., learning and recall of recently learned information). For a diagnosis of dementia, impairment in another cognitive domain (language, visuospatial, executive functions) is required.
  - The diagnosis of typical AD should not be applied when there is evidence of (a) substantial concomitant cerebrovascular disease, a history of a stroke temporally related to the onset or worsening of cognitive impairment; or the presence of multiple or extensive infarcts or severe white matter hyperintensity burden; or (b) core features of Parkinson's disease or dementia with Lewy bodies other than MCI or dementia; (c) prominent features of behavioral variant frontotemporal dementia; or (d) prominent features of semantic variant primary progressive aphasia or nonfluent/agrammatic variant primary progressive aphasia; (e) evidence for another concurrent, active neurological disease, or a non-neurological medical comorbidity or use of medication that could have a substantial effect on cognition.

- The **"clinically atypical"** group is intended for in whom underlying AD is considered a possible cause of MCI and dementia, but are not "clinically typical" because they have one or more of the following features:
  - The primary symptoms are not related to memory (e.g. primary deficits in executive functions, language, visuospatial, psychiatric or motor functions)*

- Presence of significant co-morbidities that can contribute to cognitive decline (e.g. medical conditions, pre-existing neurological or psychiatric conditions; substance abuse or other drug effects)
- The course of clinical progression is atypical (i.e. not slowly and gradually progressive)
- The clinical course has mixed features of AD and non-AD dementing illnesses (e.g. Parkinson's disease, Lewy body disease, frontotemporal dementia)

***Note**: Non-amnestic phenotypes associated with AD neuropathology, such as language-predominant presentation (also known as logopenic-variant primary progressive aphasia,[62] visuospatial/visuoperceptual presentation (also known as posterior cortical atrophy[63] and dysexecutive presentation (also known as frontal-variant AD[64] should be included in the "clinically atypical" group.

4.2 Exclusion Criteria

1. Normal cognition or subjective complaints that are not verified by cognitive testing or key informant.
2. Knowledge of amyloid status, in the opinion of the referring dementia expert, may cause significant psychological harm or otherwise negatively impact the patient or family.
3. Amyloid or tau status already known to patient or referring clinician based on prior imaging or cerebrospinal fluid analysis.
4. Previous amyloid PET scan obtained.
5. Current or previous treatment with an anti-amyloid agent.
6. Current or previous enrollment in an anti-amyloid therapeutic trial.
7. Scan is being ordered solely based on a family history of dementia, presence of apolipoprotein E (ApoE) 4, or in lieu of genotyping for suspected autosomal mutation carriers.
8. Scan being ordered for nonmedical purposes (e.g., legal, insurance coverage, or employment screening).
9. Cancer requiring active therapy (excluding non-melanoma skin cancer).
10. Hip/pelvic fracture within the 12 months prior to enrollment.
11. Body weight exceeds PET scanner weight limit.
12. Currently pregnant or planning to become pregnant within 90 days of registration.
13. Life expectancy less than 24 months based on medical co-morbidities.
14. Residence in a skilled nursing facility (assisted living facility is not an exclusion criterion).

4.3 Referring Physician (Dementia Specialist) Practice Selection

Each practice site must complete a site feasibility questionnaire and receive and invitation from the study to participate. Each participating dementia specialist practice will be included in a contractual agreement with the ACR that will ensure eligibility and facilitate payment for submitted data. Payment will be made to the practice via Bank of America into the bank account identified by the practice. A Form W-9 will be collected prior to practice activation. Each practice must obtain Institutional Review Board (IRB) approval by the central IRB contracted for the study and an IRB-stamped informed consent form for use in consenting study participants. Recruitment material will be produced centrally and provided to the practice for contact information customization. Each practice must have at least one approved dementia specialist before the practice may be activated on the study.

4.4 Dementia Specialist and Research Staff Eligibility

For the purpose of this study, the criteria to be eligible to participate as a dementia specialist will be consistent with the definition in the AUC publication.[51] A dementia specialist is a self-identified physician trained and board-certified in neurology, psychiatry or geriatric medicine who devotes a substantial proportion (≥25%) of patient contact time to the evaluation and care of adults with acquired cognitive impairment or dementia.[51] Dementia specialists will be recruited through societies such as the International Association of Gerontology and Geriatrics, American Academy of Neurology, American Society of Neuroradiology, and clinician outreach through psychiatrists, members of the Alzheimer's Association, as well as media outreach. A clinician who is board certified in another specialty but otherwise appears to meet the AUC definition of a dementia specialist may apply to the New IDEAS Study team for an exemption by submitting his/her curriculum vitae and a letter of justification outlining his/her experience. Dementia specialists must be enrolled in the Medicare Patient Enrollment Chain and Ownership System (PECOS) to provide services to Medicare patients, even if they have opted to be non-participating physicians. Each participating dementia specialist practice will be included in a contractual agreement with the ACR that will ensure eligibility and facilitate payment for submitted data.

To be eligible to participate in the study, the dementia specialist and research staff must also:

- Review the New IDEAS training modules on the study website.
- Complete training for research with human subjects (e.g., CITI, GCP).
  - Documentation of human subjects training must be uploaded to the practice registration page within the study database for all dementia specialists. Practice staff who are designated consenting authorization by their site's principal investigator also need to upload documentation of training.
  - Documented training must have occurred within 3 years prior to profile registration.
  - If training is needed, ACR will cover the cost through affiliation with CITI. Users may affiliate with the American College of Radiology during their CITI registration process in order to complete the "Human Subject Research – Basic" course, free of charge.

4.5 PET Facility Eligibility and Registration Requirements

An eligible PET facility will have experience in PET brain imaging. A participating PET facility may be (1) free standing and accredited by either the ACR, the Intersocietal Accreditation Commission (IAC) or RadSite or (2) hospital based and accredited by the Joint Commission (or another Medicare-approved hospital-based accrediting organization) with or without additional accreditation by ACR, IAC or RadSite. The PET facility must document that it has experience performing brain PET, PET/CT or PET/MRI with one of the FDA-approved amyloid imaging agents or with F-18 fluorodeoxyglucose or with both.

Only facilities with full-ring BGO, GSO, LSO or LYSO PET, PET/CT or PET/MRI scanners are eligible to participate; partial-ring systems and dedicated NaI systems are not eligible for use in the New IDEAS Study. The entity applying as a PET facility should be the entity that bills Medicare for either the technical charges or the global charges for PET studies. The PET facility must execute a business associate agreement (BAA) and contractual agreement with the ACR before the facility will be activated as a collaborator on the study. All required documents will be available for download at www.ideas-study.org. Participating radiologists/nuclear medicine physicians reading images must 1) be board certified, 2) have completed vendor-provided reader training for each radiopharmaceutical agent to be utilized, and 3) agree to participate in the study and adhere to protocol procedures. Radiologists/nuclear medicine physicians also must be enrolled in the Medicare Patient Enrollment Chain and Ownership System (PECOS) to provide services to Medicare patients, even if they have opted to be non-participating physicians.

4.6 Recruitment and Screening

4.6.1 Screening and Responsibilities

All referrals to the study and for amyloid PET will come from dementia specialists who will confirm eligibility. As articulated in AUC, substantial clinical expertise and experience are required to determine if amyloid PET is indicated, and to correctly interpret the results of amyloid PET in the context of a specific clinical syndrome, and after appropriate exclusion of alternative causes of cognitive impairment. In order to ensure a diverse patient population in the study cohort, and in order to avoid potential bias related to disproportionate recruitment by a single dementia specialist, the maximum enrollment by any individual dementia specialist will be capped. Please refer to the enrollment cap policy found in the Policies section of the New IDEAS website.

To meet inclusion criteria, patients will be required to have a diagnosis of MCI or dementia established within 24 months following a standard-of-care assessment for cognitive impairment as defined in the American Academy of Neurology practice parameter.[49] The standard assessment includes a medical and neurological history and physical examination, mental status testing with formal neuropsychological testing if indicated, to include the MOCA or MMSE tests, laboratory tests for systemic/reversible causes of cognitive impairment (at minimum CBC, standard blood chemistry profile, TSH, vitamin B12) within 12 months of enrollment, and structural neuroimaging (head CT or MRI) within 24 months. It is required that elements of the standard assessment be either performed or verified by the dementia specialist (i.e., no need to

repeat tests that have been ordered prior to the dementia specialist's evaluation, but the data need to be reviewed prior to referral for amyloid PET).

If a participant has been evaluated by the referring dementia specialist as described above within the last 3 months, re-evaluation at the time of consent/registration is not necessary, and the pre-PET form, which is to be completed only by the approved dementia specialist, may be obtained from that prior evaluation. However, the benefits and risk of learning amyloid status, including potential psychological impact, should be discussed again at the time of consent if a formal re-evaluation is not performed. The dementia specialist will be responsible for identifying patients with MCI and dementia who meet inclusion criteria, and for screening these candidates for exclusion criteria.

4.6.2 Informed Consent

The referring dementia specialist and/or authorized designee will obtain informed consent using the IRB-approved ICF. A face to face, in-person informed consent process is the preferred best practice, however remote and electronic consent is allowed. The remote informed consent process should occur in a way that is similar to what would be conducted in-person under normal circumstances. These conversations may occur via telephone, conference call, video conferencing, telemedicine, or other methods used by the consenting site. The IRB approved informed consent must be sent to the participant prior to engaging in the informed consent conversation, so the participant can reference the document during the conversation. When obtaining remote consent, researchers must document (1) how the ICF was transmitted to the participant (e.g., email, fax, mail, etc.) and (2) how the participant's signature was obtained. The consenting site must ensure the fully executed ICF is returned to the study team. Prior to consent, dementia specialists will determine if the patient has capacity to consent to research based on the dementia specialist's expertise and training. If the patient is determined by the dementia specialist to lack capacity, proxy consent will be allowed. Patients or their proxies will be consented for saliva collection and will be given the opportunity to accept or decline consent to image archiving and blood collection. All participants, or their proxies as appropriate, will provide consent to allow access to their healthcare data to complete study electronic case report forms (eCRFs) as well as to their Medicare administrative claims for up to three years for follow-up purposes. If planning on obtaining informed consent remotely, thorough guidance can be found on the study website.

4.6.3 Participant Registration in the Study

The referring dementia specialist (or an authorized designee who has obtained the registrar role enabling access to the database) will register the participant in the database, including documentation that participant consent has been obtained. Registration is processed via a secure, role-based online application where identifying information about the patient and dementia specialist is introduced on the Patient Registration eCRF. The participant's self-identification of their race will be collected as part of the registration process to verify availability within defined cohorts, as advised by the Centers for Medicare and Medicaid Services (CMS). The database will issue a unique study case number for that patient and send a confirmation e-mail to the dementia specialist practice and the PET facility. The dementia specialist or designee and the PET facility will work closely with the participants to coordinate the timing of the amyloid PET scan to

ensure scan completion no later than 60 days after the pre-PET eCRF submission. The amyloid PET will be read locally at the PET facility, which will provide the ACR Center for Research and Innovation (CRI) Data Management Center with the amyloid PET report text and an Amyloid PET Assessment eCRF completed by the radiologist/nuclear medicine physician who interpreted the study. The dementia specialist will be responsible for:

- Completing the pre-PET eCRF at the time of PET referral after formulation of the patient's intended management plan prior to PET;
- Communicating the results of the amyloid PET imaging to the patient after the scan is performed at which point changes in treatment may be implemented;
- Completing the post-PET eCRF at the 90-day post-PET follow up visit and submitting the data no later than 30 days after that visit (visit window: 60-120 days post PET). The actual management will be recorded in the post-PET eCRF based on information collected during the in-person follow-up clinical visit.

Note: The ACR Operations Center uses a role-based database; each user is required to have a username/password and current e-mail address for access and receipt of notifications/alerts. Per ACR policy, passwords must be changed every 90 days and physicians must do their own data entry.

4.7 Inclusion of Women and Minorities

Both men and women and members of all ethnic groups are eligible for this study. Given our goal of recruiting a diverse population, the study pre-specifies recruitment caps based on self-identified race/ethnicity.

4.7.1 Strategies for Minority Recruitment

Clinical trial participation rates are extremely low among racial and ethnic minorities,[65] which limits the generalizability of research results, decreases acceptability of diagnostic and treatment options, and impacts the relevance of outcomes. These differences can further broaden existing disparities in health outcomes. Fundamental challenges related to minority recruitment are well known and include key barriers at the scientist, participant, and study levels (see Box 1).[57-59] For example, scientists often lack inclusive recruitment strategies, and participants have limited knowledge about the meaning of clinical trials and know of few opportunities to participate in them. There are also study-level obstacles to implementing recruitment strategies including limited resources and expertise to translate or adapt literacy level of documents,

Box 1. MOST FREQUENTLY CITED BARRIERS TO MINORITY PARTICIPATION IN CLINICAL TRIALS

Scientist –level barriers
- Lack of sociocultural theoretical and conceptual models to guide recruitment
- Lack of innovative and inclusive recruitment strategies
- Insufficient long-term relationships with community organizations serving underrepresented groups
- Few researchers or staff from underrepresented groups

Participant-level barriers
- Perceived harms, fear
- Mistrust or lack of trust of research
- Costs, transportation, access, convenience
- Info unavailable in preferred language
- Lack of knowledge about clinical trials

Study-level barriers
- Budgets that have not factored in recruitment costs
- Resources/expertise to translate documents or adapt literacy level
- Expertise to culturally tailor documents
- Lack of culturally congruent research staff
- Lack of relationships with community organizations

limited expertise to culturally tailor recruitment strategies, limited expertise to culturally tailor recruitment strategies, inadequate budget, and lack of culturally congruent research staff.

This study will use strategies and tools Drs. Wilkins and Dilworth-Anderson have developed and successfully implemented in the recruitment of thousands of minority research participants for clinical research, including for studies with more than 50% minority older adults.[56, 66-71] We will also leverage resources of The Alzheimer's Association, the Vanderbilt Recruitment Innovation Center (PIs: Wilkins and Harris), the only national recruitment center funded by NIH and the UNC Center for Health Equity Research (Director: Giselle Corbie-Smith, Faculty Associate: Dilworth-Anderson). [72,73] The overall approach to minority recruitment will engage community partners and representatives from minority communities across the spectrum of recruitment from awareness of the study to dissemination of study results (see Figure 2).

Figure 2. Multifaceted strategies toward increasing study awareness and engagement in under-represented minorities.

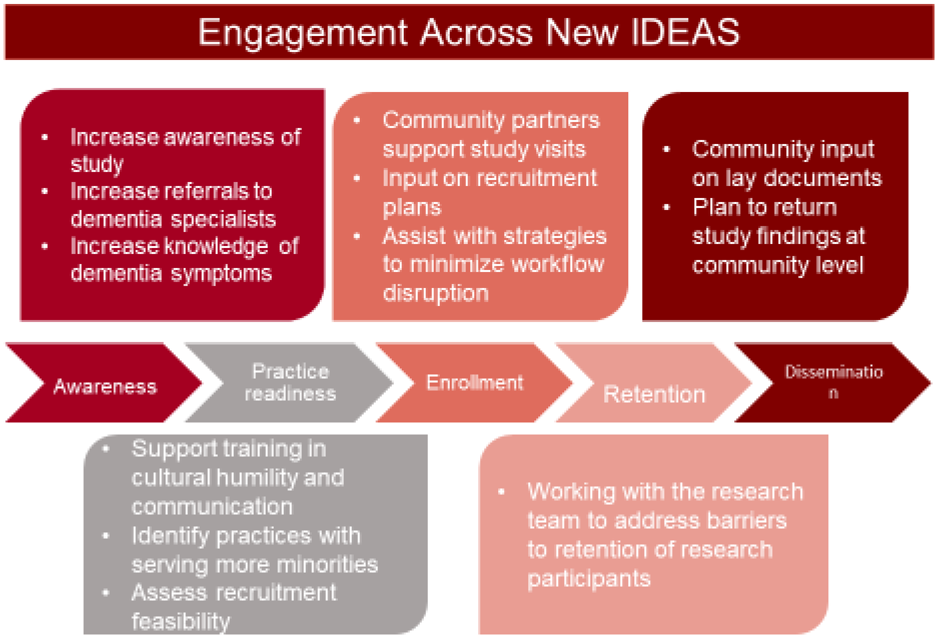

To ensure we achieve our minority accrual goals of 2,000 Blacks/African Americans and 2,000 Latinx/Hispanics, we will implement a three-phase approach:

Phase 1: Identify 10 Regions with Dementia Specialists with Capacity to Engage Minority Patients

- ***Prospectively, identify dementia specialists with higher percentages and/or large numbers of minority patients***. We will adapt the screening tool used by dementia specialists in IDEAS and include a) an assessment of the percentage and number of minority patients in the practice, b) prior experience recruiting minorities, and c) availability of staff with experience engaging minorities.

- ***Retrospectively, engage dementia specialists who participated in IDEAS and recruited higher percentages of minority patients***. We will contact all dementia specialists with higher minority recruitment rates and proactively recruit them to participate in this study. Registration of these dementia specialists will be prioritized and we will specifically elicit their feedback on strategies to optimize clinician engagement. The success of the original IDEAS Study resulted in an extensive site network, which may be leveraged for this study. However, since this study design requires only approximately one-third the number of participants, it is anticipated that the full network will not be involved. Rather, site selection will be targeted with criteria to include geographic location, research experience and staffing, and previous accrual success.

Phase 2: Launch Local Community Engagement

- ***Phased Local Launches***. From the identification of dementia specialists, we will identify 10 regions of the U.S. (major urban center or region of a state) to target local community-engagement. One region will be identified with multiple dementia specialists who participated in IDEAS. We will jump-start recruitment in this one region, and then have a three-month delayed launch for the other 9 regions.
- ***Adapt recruitment materials to local cultural contexts.*** Using templates, images and culturally appropriate language developed by the Vanderbilt Recruitment Innovation Center, we will create study specific recruitment materials targeted towards each minority group. Based on prior experience recruiting minority older adults, we will create recruitment materials intended for the family caregivers, adult children, and adult grandchildren of potential participants, as they are often more involved in decision making about clinical trials.[14] Materials will be created in English and Spanish. Community representatives will provide feedback on the materials.
- ***Engage community organizations to form local community-based recruitment teams.*** Leveraging the robust national network of the Alzheimer's Association, we will identify Chapters with active involvement in minority communities to form local community-based recruitment teams. Local community-based recruitment teams will disseminate recruitment materials to trusted churches, social, civic, and cultural groups in African American and Latinx communities. Local community-based recruitment teams will provide feedback about local barriers to participation. Adherence assessors will support local community-based recruitment teams in overcoming barriers to recruitment. Tiered incentive structures will be used to compensate teams. Local chapters will also assist with identification of PCPs and dementia specialists with strong ties to minority communities.
- ***Engage community organizations committed to reducing disparities in AD to form a recruitment advisory board.*** Bi-directional communication between researchers and community is standard practice to develop trust, study awareness, and ultimately satisfactory recruitment and retention of minorities. Local community contacts will identify members of organizations with strong ties to minority communities to serve on a Recruitment Advisory Board, which will provide bi-directional communication—receiving quarterly retention protocol adherence reports—and providing input to Drs. Dilworth-Anderson and Wilkins. They will also assist with communication and leverage their well-established networks and social media.

Phase 3: Participant/Caregiver Engagement

- ***Provide the additional resources required to recruit and retain minorities.*** The costs to recruit and retain minorities in clinical studies are often underestimated. In general, the staff and time required to recruit racial and ethnic minorities is twice that required for the general population. For example, clinicians and nursing staff will likely spend more time discussing research studies, speaking with family members and caregivers involved in decision-making, reviewing consent forms, and scheduling and confirming appointments. Recognizing these additional burden/costs to maintain patient/caregiver engagement, we will allocate adequate resources for assistance.
- ***Provide patient/caregivers follow-up support to engage and retain.*** The burden of navigating the specialist appointments, PET facilities, and fees is a major deterrent to study participation. In general, participants and their caregivers benefit from additional support to ensure completion of all study activities. Research personnel will serve as "navigators" to provide logistical support and ensure completion of study activities, thereby reducing attrition.

4.8 Monitoring Enrollment and Retention

An important element of a successful recruitment is tracking and responding to deviations from anticipated recruitment and retention rates. Particularly with the use of local recruitment teams, monitoring of adherence to protocols and bi-directional communication is key for timely responsiveness to local barriers to participation. We will proactively collect information from local recruitment teams and monitor progress to promptly address issues as they arise. We will monitor progress with onsite observation of local community recruitment team's protocol adherence. We will generate enrollment reports with projected enrollment and retention rates alongside actual enrollment and retention rates, and weekly reports will be reviewed at team meetings. At a predetermined threshold of discordance, alerts will trigger notifications and the recruitment and retention team will identify challenges and develop plans to address the issue. A standard operating procedure will outline steps to be taken before the issue can be closed. When necessary, we will convene community engagement studios to elicit feedback from participants and families and/or clinicians to inform and adapt recruitment plans.

5.0 STUDY DESIGN AND CALENDAR

New IDEAS is an observational, open-label, longitudinal cohort study collecting participant pre-PET assessments (clinical information, and the dementia specialist ***intended*** management plan), saliva for ApoE4 genotyping, amyloid PET results, amyloid PET images (only if the subject provides consent to image archival), post-PET assessments (clinical information, and ***actual*** management 90 days (visit window: 60-120 days) after the amyloid PET imaging, and, among consenting subjects, blood for genomics and testing for other potential biomarkers. Medicare claims data from standard medical procedures will be collected directly from CMS for 12 months prior to the PET imaging and 12 months after the PET imaging. Each referring physician practice that is invited to participate will complete qualification procedures and contracting requirements. Referring dementia specialists and research staff will need to work closely with a PET imaging facility capable of performing and interpreting amyloid PET examinations. Referrals for amyloid PET will come from dementia experts; all providers will be provided educational

materials about amyloid PET that highlight the fact that amyloid pathology is not synonymous with clinical or neuropathological AD. Each individual patient will be asked to provide consent allowing his or her data collected for the study to be used for research purposes; if the patient is unable to provide consent, a proxy may consent on his or her behalf. Following is the study calendar:

**New IDEAS Study Calendar**					
**Study Procedure**	**Visit 1: Eligibility/ Registration (T1)***	**Visit 1: Pre-PET Clinical Assessment (Complete Intended Management) (T2)***	**Visit 2: Amyloid PET (Within 60 Days after Pre-PET Form Completion) (T3)**	**Disclosure of PET Results* and Biospecimen Collection (T4)**	**Visit 3: Post-PET Office Visit(90 ± 30 Days after PET (Complete Actual Management) (T5)***
Screening/Eligibility Review	X				
Informed Consent (Allowable by proxy)	X				
Case Registration Online	X				
Socio-demographic Form	X				
Saliva Collection (5)				X	
Blood Collection (6)				X	
Refer for Amyloid PET	X				
Pre-PET Form Completion (Reporting intended management (1)		X			
Amyloid PET at PET Facility / Upload to ACR TRIAD™ within 7 Days (2)			X		
Disclosure of PET Results to Patient by Dementia Specialist (3)				X	X
Post-PET Form Completion (Reporting actual management) (4)					X

* In-office clinic visit is the preferred best practice for conducting visit, however can be conducted via video conference or telemedicine platform

(1) The Pre-PET Form completed by the Dementia Specialist is required to be submitted online within 7 days after the visit/assessment.

(2) PET images must be uploaded to ACR Image Archive via TRIAD™ within 7 days after the scan completion. PET Completion Form completed by PET Facility staff must be submitted online within 7 days of the day the scan is performed. Same day submission is recommended best practice. Amyloid PET Assessment Form completed by the radiologist/nuclear medicine physician and PET Report uploaded by PET Facility staff must be submitted online within 7

days after the scan.

(3) Disclosure of the PET scan results to the patient, per standard of care, should not wait for the 90-day visit. The goal of the 90-day case report form is to capture changes in management that have been implemented between the PET scan and the 90-day visit, incorporating amyloid PET results.

(4) The post-PET Form completed by Dementia Specialist must be submitted to ACR CRI Data Management Center within 30 days after the visit/assessment. New IDEAS expects dementia experts to schedule the participant for an office visit within the 60-120 post-PET window.

(5) Saliva collection kits will be mailed directly to the New IDEAS participants after the amyloid PET visit is complete. Detailed instructions for collection and shipment back to the Biorepository will be included in the kit.

(6) For those who opt into this component, blood collection kits will be sent directly to the New IDEAS participant after the amyloid PET visit is complete. Detailed instructions will be included in the kit. Participants will then either A) take the kits to their local Quest Laboratories or B) complete the process for scheduling an at-home blood draw and have their blood drawn at-home by ExamOne Laboratories. Quest or ExamOne will be responsible for blood collection and shipment of the blood tubes to the Biorepository. Note: Blood collection tubes, sharps and other supplies for blood collection will be provided by Quest or ExamOne and not included in the collection kit.

5.1 VISIT 1: Clinical Assessment—Pre-PET Visit Reporting Intended Management

Visit 1 includes the clinical assessment and eligibility review and formulation of the dementia specialist's intended management as recorded on the pre-PET eCRF. An in-office clinic visit is the preferred best practice for conducting Visit 1, however a remote visit is allowed via video conference or telemedicine platform. An e-mail confirming participant registration will be sent to the submitting dementia specialist, the practice administrator, the registrar and the affiliated PET facility. The referring dementia specialist must complete and submit the electronic Pre-PET eCRF within 7 days of participant registration or the case will cancel and re-registration will be required; a confirmation of the pre-PET eCRF submission is automatically generated to the PET facility administrator who will then forward any such study notifications to schedulers. The Pre-PET eCRF will collect the following information: (1) the specific reason for the PET study referral; (2) the patient's working clinical assessment/provisional diagnosis; (3) the referring physician's documentation or assessment of prior evaluation and treatment for cognitive impairment; and (4) the referring physician's intended management if PET were not available.

5.1.1 Socio-demographic electronic Case Report Form (eCRF)

The referring dementia specialist (or an authorized designee who has obtained the registrar role enabling access to the database) will complete an online CRF within 7 days of participant registration. Data elements must be collected via patient self-reporting interview and will include the following:

- Patient's educational attainment, marital status, financial strain, preferred language, and living arrangements

5.1.2 Pre-PET electronic Case Report Form (eCRF)

The referring dementia specialist will complete an online CRF prior to the amyloid PET imaging and within 7 days of participant registration. Data elements to be collected include the following:

- Certification of eligibility (inclusion/exclusion criteria)
- Medical history including Covid-19 exposure
- The pre-PET clinical diagnosis:
    1) MCI or dementia

2) "Clinically Typical" or "Clinically Atypical"
3) Suspected etiologic cause/s (as a differential diagnosis)
4) Previous evaluation
5) AD drug therapy

- The dementia specialist's management plan based on the current clinical and diagnostic information, and assuming no future access to amyloid PET, including:
  1) Watchful waiting
  2) Intended changes in AD therapy (cholinesterase inhibitors and/or memantine)
  3) Intended changes in other relevant medications, including psychiatric drugs, drugs that can negatively impact cognition, drugs to treat medical conditions that can impact cognition (e.g., cardiovascular disease, diabetes), other neurological conditions, and targeted therapies
  4) Guidance about safety and planning
  5) Referral to family support systems (Alzheimer's Association for care plans, legal and safety education)
  6) Additional diagnostic procedures
  7) Referral to non-pharmacologic interventions
  8) Plans to refer individuals to clinical trials (for AD or non-AD dementia)

5.2 VISIT 2: Amyloid PET Imaging Visit

The PET scan must be completed within 60 days after completion of the Pre-PET eCRF. It may not be completed before the Pre-PET eCRF has been submitted. The PET facility will receive an e-mail notification when the Pre-PET eCRF has been completed. Performing the PET scan before the Pre-PET form is received is a serious protocol violation. If the amyloid PET is delayed for more than 60 days from the time of the Pre-PET eCRF submission by the dementia specialist, the registration will automatically cancel, sending an e-mail notification to the PET facility administrator that this has occurred. If the amyloid PET is still to be performed at a later date, it will be necessary for the participant to be re-registered and the pre-PET eCRF to be submitted anew.

When the amyloid PET scan has been completed, the PET facility staff or physician documents this by submitting the Amyloid PET Completion eCRF online as soon as possible, preferably by midnight on the day the scan was performed, but no later than 7 days. Within 7 days of the scan, the PET Facility physician or staff must upload a copy of the full PET report to the New IDEAS database and must upload the amyloid PET images to the ACR image archive, if participant consent for image archival was obtained. The radiologist/nuclear medicine physician who interprets the amyloid PET must submit the online Amyloid PET Assessment eCRF within 7 days of the scan.

5.2.1 Imaging Acquisition

The PET scan in New IDEAS is a standard of care clinical procedure. Therefore, imaging facilities should follow local policies, procedures and standards that align with the SNMMI Procedure Standard/EANM Practice Guideline for Amyloid PET Imaging of the Brain 1.0.

5.2.2 Subjectivity of Amyloid PET Interpretation

As part of the FDA approval process, each of the vendors was required to demonstrate the reliability of dichotomous qualitative PET scan reads (positive versus negative) for discriminating no or sparse amyloid plaques versus moderate or frequent amyloid plaques by comparison with brain histopathology as the reference standard. Each radiologist and nuclear medicine physician who interprets amyloid PET images as part of the New IDEAS Study is required to have completed the vendor-provided in-person or online training courses specific to the amyloid imaging agent (or agents) used at his or her participating PET facility for New IDEAS Study participants. Each vendor already has in place and will continue to provide for IDEAS Study research, consultative resources to assist PET facilities with technical aspects of amyloid imaging, including patient preparation and positioning, dosing and administration, imaging acquisition and reconstruction, and scanner and image quality. Each vendor already has in place and will continue to provide for the New IDEAS Study consultative resources to assist with image interpretation. The availability of these vendor-supported resources, which are part of standard amyloid imaging practices, are expected to ensure that amyloid imaging quality and interpretations for the New IDEAS Study meet the standard of care.

5.2.3 Digital Image Submission Using TRIAD™ to ACR PET Imaging Archive

Brain amyloid PET scans will be collected for all subjects who do not "opt out." Images will be submitted to the ACR archive within 7 days of scan acquisition using the web-based TRIAD™ application. Only case IDs of patients who do not "opt out" will be visible within TRIAD and available for image upload. This is an integrated safe-guard measure to ensure that only authorized images are transferred. TRIAD™ is the ACR image exchange application. It provides participating PET facilities a secure method to transmit subject images. TRIAD™ anonymizes and validates the images as they are transferred. Site radiology/nuclear medicine staff who will submit images through TRIAD™ will need to be establish access as part of the PET Imaging facility.

5.3 BioSpecimen Collection

Collection of participant saliva and blood, as described below, will occur after the PET Scan Visit (V2). Referring dementia practices and PET imaging facilities are not responsible for collecting any specimens or data associated with specimen collection. Collection kits with detailed instructions will be sent to the participant's address on file once the PET scan is completed. Referring dementia specialists and practice staff are responsible for accurately explaining biospecimen collection details within the protocol to all participants during the informed consent process.

5.3.1 Saliva Collection

Saliva (for ApoE4 genotyping) will be captured from all study participants. A saliva collection kit will be mailed to each participant's address on file from the New IDEAS Biorepository at the Alzheimer's Therapeutic Research Institute (ATRI) at USC Keck School of Medicine (See Appendix I). The collection kit will be mailed to the participant after the Amyloid PET Imaging Visit (V2) is completed. Saliva will be collected in-home by the participant and immediately

shipped to the New IDEAS Biorepository at ATRI USC for DNA extraction and ApoE genotyping. Detailed instructions on collection and shipment will be included in the kit that is mailed to the participant. After sample analysis is complete by ATRI, the saliva samples will then be destroyed.

5.3.2 Blood Collection

If consent is given for blood collection, a Blood Collection Kit will be sent to the participant's address on file. The participant may either A) bring the kit to their local Quest Diagnostics Laboratory or B) complete the process for scheduling an in-home blood draw and have their blood drawn at their residence by ExamOne. Quest/ExamOne personnel will then collect plasma using Quest/ExamOne blood tubes, sharps, and other phlebotomy supplies. The New IDEAS Blood Collection Kit will then be used to ship the tubes back to the New IDEAS Biorepository at ATRI. The blood kit will be mailed to the participant after the Amyloid PET Imaging Visit (V2) is completed.(See Appendix I). Given the real-world setting of the New IDEAS infrastructure, this offers a unique and first-of-a-kind opportunity for the field to test blood-based biomarkers specifically within the context of use and existing medical infrastructure within which such tests would actually have to function. Participants who agree to participate in the optional blood collection component will be compensated by the study for their time, effort, and transportation needs.

5.4 Amyloid PET Disclosure

Disclosure of amyloid PET results to the patient and family should occur as part of clinical care, and no specific timeframe or parameters for accomplishing this are dictated in this protocol. However, best practices suggest that disclosure should be done as soon as possible after the scan results are available, based on physician and patient availability. In most cases it would *not* be appropriate to wait until Study Visit 3 (90 days post-PET) to disclose results. Results should be disclosed by the referring dementia specialist, and disclosure should not be delegated to non-clinical staff. In most cases it is preferred that scan results be disclosed in person, however a remote visit is allowed via video conference or telemedicine platform. Every attempt should be made to avoid the patient receiving results directly from an electronic medical record portal. A training video will be updated prior to opening of the protocol. For more information about best practice recommendations for amyloid PET counseling, refer to "Development of a process to disclose amyloid imaging results to cognitively normal older adult research participants." At this time point, the referring dementia specialist should also recommend any subsequent changes in management that are clinically appropriate incorporating PET results and any additional clinical information. Potential management actions include further diagnostic testing/consultation, imaging, laboratory or genetic analysis, referrals and counseling for non-pharmaceutical care, and a detailed documentation of pharmaceutical treatments (started, continued, or stopped) by drug categories. Implementation and adherence to recommended changes in management made at the disclosure visit will subsequently be evaluated at the 90-day post-PET Visit (see Visit 3 below).

5.5 VISIT 3: 90 Days (± 30 Days) After Amyloid PET Scan (Visit 2)

A mandatory 90-day (from day of PET scan) clinical office follow-up is required. The same dementia specialist will complete both the Pre- and Post-PET eCRFs. The Post-PET eCRF is due within 30 days after the 90-day visit. The 90-day visit may occur within a window of 60-120 days post-PET scan date. An in-office clinic visit is the preferred best practice for conducting Visit 3, however a remote visit is allowed via video conference or telemedicine platform. Exceptions reflecting subsequent study exclusion are: a) if patients have had subsequent events leading to prolonged care in a skilled nursing facility or b) death. The Post-PET eCRF must be completed even in these circumstances, however, to document the reason the visit was not completed. In rare instances in which the patient is not able to return for clinical follow-up within the allotted time (e.g., because of geographic distance from the dementia specialist), the post-PET visit may occur by telephone contact between the dementia specialist and the patient and family. The dementia specialist will need to document this protocol variation in the post-PET eCRF, and the IDEAS Study team will contact the physician if the reason for telephone follow-up is deemed unacceptable or the frequency of telephone visits appears excessive. ***Under no circumstances is the dementia specialist permitted to delegate the post-PET contact (in person visit or telephone) to other staff or to another physician.***

At this 90-day visit(s), the actual patient management (according to the dementia specialist or others) will be documented and characterized. *Implementation and adherence to management actions recommended at the amyloid PET disclosure visit (or subsequent clinical visits, prior to the 90-day visit) will be recorded in the Post-PET eCRF*, including: further diagnostic testing/consultation, imaging, laboratory or genetic analysis, referrals and counseling for non-pharmaceutical care, and a detailed documentation of pharmaceutical treatments (started, continued, or stopped) by drug categories. The goal of the Post-PET eCRF is to capture *actual patient management* as reflected by management changes that have been implemented into patient care, in contrast with *intended management changes*, i.e., changes that have been recommended but not adopted or implemented.

Emergency room visits, and all cause hospitalizations during this 90-day interval also will be collected. If 90-day follow-up cannot be completed because the patient died, withdrew from care by the dementia specialist, withdrew consent, or was lost to follow-up, the specific reasons must be recorded on the post-PET eCRF. We will ask the dementia specialist to note on the post-PET eCRF any adverse effects reported by the patient or caregiver that are attributable to learning amyloid status.

5.6 Case Completion and Reimbursement

After all Pre- and Post-PET eCRFs have been completed and all data uploaded to the ACR CRI data management center within the required time windows by the dementia specialist, payment for the case will be made via Bank of America to the bank account registered by the referring physician practice. Upon completion of the amyloid PET scan and related data submission to the ACR CRI data management center within the required windows by the PET facility, claim(s) for the amyloid PET study may be submitted to Medicare for reimbursement.

5.7 CMS Claims Data Collection

The ACR CRI Data Management Center and Brown University will coordinate CMS claims data collection and analysis for the longitudinal cohort for Aim 3, which uses claims analysis to assess participant outcomes. The data provided by participating sites will be used for coordinating collection of CMS claims data one year after the scan. In addition, consent will be obtained to follow CMS claims for 36 months post-PET.

5.8 Central Institutional Review Board Approval for Study Participation

Participating sites will be required to use the central IRB specified by the Operations Center for regulatory approval of the protocol and informed consent documents. The primary entity charged with operationalizing this study is the New IDEAS Study team, described on the cover page of this document; the New IDEAS Study team intends to use the data it is collecting for research purposes in accordance with the consent for such use provided by the patient and the patient's treating doctor. A central IRB will be charged with reviewing and approving the consent forms prior to study activation at the referring physician's site. Each site must provide a copy of the final consent form to ACR, and must notify ACR and the central IRB if any revisions are made to the consent during the study.

5.9 New IDEAS Study and CMS-Suggested Design Specifications

In its National Coverage Determination (CAG-00431N), CMS indicated it would cover one amyloid PET scan per patient through coverage with evidence development (CED), under §1862(a)(1)(E) of the Act, in clinical studies that meet certain specific criteria. The primary endpoint of interest to CMS is that clinical studies under the CED program must address one or more aspects of the following questions. The New IDEAS Study has a particular focus on Criterion/Question 2, below, in African-American and Hispanic/Latinx populations:

For Medicare beneficiaries with cognitive impairment suspicious for AD, or who may be at risk for developing AD:

1) Do the results of amyloid PET lead to improved health outcomes? Meaningful health outcomes of interest include: avoidance of futile treatment or tests; improving, or slowing the decline of, quality of life; and survival.
2) Are there specific subpopulations, patient characteristics or differential diagnoses that are predictive of improved health outcomes in patients whose management is guided by the amyloid PET?
3) Does using amyloid PET in guiding patient management, to enrich clinical trials seeking better treatments or prevention strategies for AD, by selecting patients on the basis of biological as well as clinical and epidemiological factors, lead to improved health outcomes?

Table 4. CMS National Coverage Standards of Scientific Integrity and Relevance to Medicare Beneficiaries for CED Assessments

Element	New IDEAS Study
The principal purpose of the research study is to test whether a particular intervention potentially improves the participants' health outcomes.	Yes. See Aims 1-3 and statistical plan.
The research study is well supported by available scientific and medical information or it is intended to clarify or establish the health outcomes of interventions already in common clinical use.	Yes. Evidence indicates that amyloid PET will lead to more accurate diagnosis of the cause of MCI or dementia, with resultant appropriate change in management. Data from the first IDEAS study indicates positive amyloid PET results are associated with lower risk for hospitalizations and emergency room visits in comparison with negative scans. Converging data suggest that Blacks/African-Americans and Latinx/Hispanics may be at higher risk for dementia than non-Hispanic Whites.[1,25] The study will provide novel insights into the causes and predictors of cognitive impairment in these high-risk groups.
The research study does not unjustifiably duplicate existing studies.	Yes. The over-arching goal of the New IDEAS Study is to inform amyloid PET coverage decisions by studying the association of amyloid PET results with changes in health outcomes in a broad and diverse population of Medicare beneficiaries with cognitive impairment. New IDEAS will address gaps in the original IDEAS Study by emphasizing recruitment of Blacks/African Americans and Latinx/Hispanics, adding Medicare beneficiaries with early-onset (age < 65) cognitive impairment and comparing outcomes in patients with typical versus atypical clinical presentations. The study adds saliva collection for genotyping of ApoE, an important risk factor for brain amyloidosis, in all participants. The study will replicate and extend observations from the first IDEAS study regarding the association between PET result and health outcomes in a more clinically and ethnoracially diverse population.

Element	New IDEAS Study
The research study design is appropriate to answer the research question being asked in the study.	Yes.
The research study is sponsored by an organization or individual capable of executing the proposed study successfully.	Yes. The investigators and organizations managing this study have extensive prior experience with large CED studies.
The research study is in compliance with all applicable Federal regulations concerning the protection of human subjects found at 45 CFR Part 46. If a study is regulated by the Food and Drug Administration (FDA), it must be in compliance with 21 CFR parts 50 and 56.	Yes. The study protocol and consent documents will be approved by a central IRB. Dementia specialists and designated staff will be required to complete training in human subjects protections.
All aspects of the research study are conducted according to appropriate standards of scientific integrity in accordance with the International Committee of Medical Journal Editors (ICMJE).	Yes.
The research study has a written protocol that clearly addresses, or incorporates by reference, the standards listed here as Medicare requirements.	Yes.
The clinical research study is not designed to exclusively test toxicity or disease pathophysiology in healthy individuals. Trials of all medical technologies measuring therapeutic outcomes as one of the objectives meet this standard only if the disease or condition being studied is life threatening as defined in 21 CFR §312.81(a) and the patient has no other viable treatment options.	Yes. The clinical research study is not designed to exclusively test toxicity or disease pathophysiology in healthy individuals.
The clinical research study is registered on the ClinicalTrials.gov website by the principal sponsor/investigator prior to the enrollment<br>of the first study subject.	Yes. The study has been registered (identifier NCT-TBD).
The research study protocol specifies the method and timing of public release of all pre-specified outcomes to be measured including release of outcomes if outcomes are negative or the study is terminated early. The results must be made public within 24 months of the end of data collection. If a report is planned to be published in a peer reviewed	Yes. These reporting standards were all achieved in our prior work with the NOPR. The IDEAS Study Aim 1 results have already been published (Rabinovici, et al., JAMA 2019)[1], and preparation of IDEAS Aim 2 results for publication is in process.

Element	New IDEAS Study
journal, then that initial release may be an abstract that meets the requirements of the ICMJE. However a full report of the outcomes must be made public no later than three (3) years after the end of data collection.	
The research study protocol must explicitly discuss subpopulations affected by the treatment under investigation, particularly traditionally underrepresented groups in clinical studies, how the inclusion and exclusion criteria effect enrollment of these populations, and a plan for the retention and reporting of said populations on the trial. If the inclusion and exclusion criteria are expected to have a negative effect on the recruitment or retention of underrepresented populations, the protocol must discuss why these criteria are necessary.	Yes. A primary aim of the study is to recruit self-identified African-Americans and Hispanics/Latinx.
The research study protocol explicitly discusses how the results are or are not expected to be generalizable to the Medicare population to infer whether Medicare patients may benefit from the intervention. Separate discussions in the protocol may be necessary for populations eligible for Medicare due to age, disability or Medicaid eligibility.	Yes. This study will address several major subpopulations of Medicare beneficiaries who may potentially benefit from amyloid imaging: these include patients with MCI and those with dementia of uncertain cause after conventional diagnostic evaluation, patients with typical AD presentations of MCI or dementia, younger Medicare patients (< age 65), and minority subpopulations.

5.10 New IDEAS Study Website and Data Collection

The New IDEAS Study website (www.ideas-study.org) is the portal for all facility registrations, case registrations, and data entry of the eCRFs. Instructional and informational material are available for downloading from the website by participating facilities and other interested parties. Table 5 includes the data collection timelines.

Table 5. Data Collection Timelines

Form	Completed By:	Due Date Requirements
Case Registration Form	Dementia Specialist or registrar	After patient consent
Socio-demographics Form	Coordinator/Clinical Staff	Within 7 days of case registration
Pre-PET Form<br>(Clinical Assessment Form)	Dementia Specialist	Within 7 days of case registration
Amyloid PET Completion Form	PET Facility	Within 7 days ofthe day the scan was performed
Amyloid PET Report Submission Form	PET Facility	Within 7 days after completion of amyloid PET
Amyloid PET Assessment Form	Radiologist/Nuclear Medicine Physician	Within 7 days after completion of amyloid PET
Post-PET Form<br>(Clinical Assessment Form)	Same Dementia Specialist who has Completed Pre-PET Form	Within 30 days after completion of the Post-PET 90-Day Visit (60-120 day window)

6.0 CONFLICT OF INTEREST

Any New IDEAS Study co-chair who has a conflict of interest with this study (such as patent ownership, royalties, or financial gain greater than the minimum allowable by ACR policy) must fully disclose the nature of the conflict of interest to the ACR.

7.0 DATA PROTECTION, ACCESS, AND PUBLICATION

7.1 Data Access and Publication Policy

An essential goal of the New IDEAS Study is to produce an invaluable data resource for the Alzheimer's Disease research community at large. The New IDEAS Study will be open to and encourage data sharing of the resources collected during this project. The New IDEAS Study will be cooperating with external groups to make de-identified data, via safe-harbor standards, available to the Alzheimer's Disease research community. However, no part of the results of the study obtained under this protocol, nor any information provided to the investigator for the purposes of performing the study, will be published or passed on to any third party without the consent of New IDEAS Study leadership. Any investigator involved in this study is obligated to provide ACR with complete test results and all clinical data obtained from the participants in this protocol. Investigators will follow the New IDEAS Data Access and Publication Policies posted at the study's website www.ideas-study.org. These policies provide information and guidelines

on how to request access to clinical and imaging data and biorepository data or biological samples archived during the study.

7.2 Data Privacy

Protected Health Information detailed in the New IDEAS study Case Report Forms will be stored on secure servers at the American College of Radiology (ACR). The Alzheimer's Therapeutic Research Institute (ATRI) will have access to the participants' names and mailing addresses during the shipping and handling procedures as described in Appendix 1. PHI will only be accessed within the ACR's secure study portal, never to be downloaded, copied, or removed from the ACR server. A unique ID number will accompany the specimen collection materials and samples will be stored with only the unique ID number for analyses.

Brown University Statistical Center will serve as the statistical center for New IDEAS and have access to de-identified case report forms in the study database for statistical analyses and ongoing study reports.

Participants in the New IDEAS study have the option to provide additional consent to be contacted for participation in future research opportunities. Participants that provide their additional consent to this optional component, give permission for their patient identifiable data (contact information) to be provided to TrialMatch®, a service offered by the Alzheimer's Association that assists interested parties in finding appropriate clinical trials. TrialMatch® will only contact consenting participants regarding studies that have been approved by a committee of New IDEAS investigators and collaborators. These approved studies will add to the body of knowledge about dementia, especially when combined with information collected by New IDEAS itself, and, by enrolling from the New IDEAS cohort, may also provide insight on amyloid PET scans that New IDEAS alone cannot find. None of the approved studies are an official part of New IDEAS and New IDEAS has no influence over their procedures, but all the studies are complementary to the goals of New IDEAS. No data will be transferred without a comprehensive Data Use Agreement in place with between the ACR and the requesting party.

Data will be regularly exchanged throughout the New IDEAS enrollment period. The New IDEAS coordinating center will prepare Excel spreadsheets listing the names, addresses, telephone numbers, e-mail addresses and referring physician names for every patient who indicated an interest in being contacted about additional research who has completed their New IDEAS Amyloid PET scan. The list will also indicate whether the patient signed the consent form himself or herself, as TrialMatch® agents may need to speak with a person authorized to make medical decisions instead of speaking directly with the patient. In addition, the New IDEAS coordinating center will provide at least 10 Linking IDs. These Linking IDs are randomly created numbers unique to each New IDEAS participant and each add-on study. They allow the New IDEAS coordinating center to identify a case record in the New IDEAS database as one matching records from an add-on study. If the add-on study wishes to request data for specific patients from the New IDEAS database to link with the data from the add-on study, they can provide a list of the Linking IDs.

The Excel spreadsheets will be encrypted, and placed on the ACR dropbox for TrialMatch® staff to retrieve. Passwords to open and decrypt the files will be sent separately from the dropbox link.

ACR staff will delete the files from the dropbox after TrialMatch® staff have retrieved them. TrialMatch® staff will return the spreadsheets using the ACR dropbox with indicators showing which patients no longer wish to be contacted. All of these details will be outlined in a Data Use Agreement between ACR and TrialMatch®.

7.3 Record Retention

FDA and ACR regulations require that all records related to human subject research be retained by the institution and Investigator for at least 2 years after the completion of the research. Records should be kept in either printed or electronic form and be readily accessible for inspection at reasonable times [21.CFR.312.62(c)]. ACR requires that each institution and investigator associated with the New IDEAS retain all records to meet the DHHS requirements.

All sites participating in New IDEAS are required to use Advarra Inc. as the IRB of record. Advarra will maintain all records of studies on their CIRB site for 3 years after the closure of a study as lined out by their Handbook and per 45 CFR 46.115(b) DHHS guidance.

8.0 STATISTICAL CONSIDERATIONS

8.1 Aim 1

Aim 1: To compare 12-month claims-derived health outcomes in amyloid PET-positive versus amyloid PET-negative individuals presenting with MCI and dementia in the entire study cohort of diverse Medicare beneficiaries.

8.1.1 Primary Objective

To compare the proportions of participants with a hospitalization within 12 months, between amyloid PET-positive and amyloid PET-negative participants in the study, separately for patients with MCI and dementia.

We hypothesize that positive amyloid PET will be associated with lower proportions of participants with hospitalizations over 12 months in patients with MCI and dementia.

Analysis strategy. We will perform the analysis for the primary objective separately for participants with MCI and dementia. For each subgroup, estimates of the proportion of participants with 12-month hospitalization will be derived and compared via a logistic model using a level 0.025 test in order to account for the two simultaneous comparisons.

Sample size considerations. Based on our assumption that the ratio of participants with MCI and Dementia in this study will be between 60/40 and 40/60, we expect that the smaller of the two cohorts will have a sample size of 2800. Assuming a 10% rate of cases with missing information, the effective sample size in the smaller group is expected to be 2520. For power considerations we assumed the proportion of positive amyloid PET scans will be around 50%. This sample size will provide power 80% to detect an Odds Ratio of at most 0.74, using a two-sided test at level 0.025., analogous to the effect observed in the first IDEAS study (Table 1). The power will be higher for

smaller values of the OR. For example, the effective sample size will provide power 86% to detect an OR of 0.7. Considerably larger sample sizes would be required to detect higher ORs. For example, an effective sample size of 5273 would be required for an OR of 0.8.

8.1.2 Secondary Objectives

1) To compare the proportions of participants with an ED visit within 12 months, between amyloid PET-positive and amyloid PET-negative participants in the study, separately for patients with MCI and dementia.

We hypothesize that positive amyloid PET will be associated with lower proportions of participants with ED visits over 12 months in patients with MCI and dementia.

2) To compare the proportions of participants who had a preventable hospitalization (i.e., related to ambulatory care-sensitive conditions) within 12 months, between amyloid PET-positive and amyloid PET-negative participants, separately for patients with MCI and dementia.

We hypothesize that positive amyloid PET will be associated with lower proportions of participants with preventable hospitalizations over 12 months in patients with MCI and dementia.

3) To compare overall resource utilization within 12 months between amyloid PET-positive and amyloid PET-negative study participants, separately for patients with MCI and dementia.

We hypothesize that positive amyloid PET will be associated with lower overall resource utilization over 12 months in patients with MCI and dementia.

Analysis strategy. The analytic strategy for secondary objectives 1 and 2 will be similar to the strategy for the primary objective. For each question, a logistic model will be used, with the corresponding response variable.

For the analysis of secondary objective #3, overall resource utilization will be measured by total Medicare cost during the 12-month period following baseline testing. We will use linear mixed-model regression to compare total Medicare cost, after an appropriate transformation of the response variable. To adjust for multiplicity of inferences, the primary objective and the secondary objectives (1, 2, and 3) will be tested in a hierarchical way, using the order in which they are listed.

8.1.3 Exploratory Objective

To evaluate whether the association of amyloid PET result with 12-month hospitalizations is mediated by changes in each component of the composite management endpoint.

We hypothesize that differences in proportions of 12-month hospitalizations between amyloid PET-positive and amyloid PET-negative patients will be mediated by changes in the composite management endpoint, as defined in the first IDEAS study.

Analysis strategy: In this mediation analysis, patient management change following amyloid PET will be assessed using the approach reported earlier in Rabinovici et al. 2019[1]. For each patient we will derive indicators of management change in each of three domains (AD drug therapy, other drug therapy, and counseling and future planning) and the overall composite of management change in any domain. The association between amyloid PET results (binary predictor) and each of the four management change variables will be assessed using logistic regression models. The indicators of management change will then be included as independent variables in logistic models predicting 12-month hospitalization and ED visits. Separate multivariable logistic regression models will be fitted to the binary 12-month hospitalization and ED visit response variables. In addition to the management change variables, these models will include the following independent variables: binary indicators of amyloid test status and level of impairment (MCI/dementia), their interaction, an indicator for typical/atypical clinical presentation, demographic variables (age, sex, race/ethnicity, education), dual eligibility, and comorbidity indicators (including epilepsy, MS, Parkinson's, delirium, depression, schizophrenia, bipolar disease, diabetes, cerebrovascular disease, CHF, COPD, dyslipidemia, hypertension, ischemic disease, acute myocardial infraction, atrial fibrillation, chronic kidney disease, stroke TIA, and traumatic brain injury). We expect that missing data in the response and the independent variables of primary interest will be negligible. We will use multiple imputation to account for substantial missingness, if it occurs.

8.2 Aim 2

Aim 2: To describe the association of amyloid PET findings with changes in patient management and 12–month claims-derived health outcomes among Blacks/African Americans, Latinx/Hispanics and Whites/Caucasians presenting with MCI and dementia.

8.2.1 Primary Objective

In each ethnoracial subgroup and level of impairment (MCI or dementia), to estimate the rate (proportion) of change between the pre-PET care plan and the 90-day implemented post-PET care plan, in a composite defined as change in one or more of the following items:

- AD-specific medications (cholinesterase inhibitors and memantine)
- Non-AD medications related to CNS conditions or risk factors
- Counseling about safety and future planning.

We hypothesize that, within each ethnoracial sub-group, the frequency of 90 day post-PET changes in the management composite endpoint will be ≥ 30%.

Analysis strategy: The proportion of change in overall management will be estimated in each of six cells defined by the three primary ethnoracial categories (Black/African American, Latinx/Hispanic, and White/Caucasian) and the two levels of impairment (MCI and dementia) and will be compared to a minimum threshold of 30%. Wilson confidence intervals for proportions

will be reported. Tests will be conducted at level 0.0083 (=0.05\6) (two-sided) to account for the six simultaneous comparisons. Rates of change in smaller ethnoracial subgroups (e.g. Asian Americans) will also be estimated, but we anticipate there will be low power to compare these estimates of 30% to the threshold value.

Sample size considerations: Assuming that the ratio of MCI to dementia cases is between 60/40 and 40/60 for each subgroup, the smallest sample size for cells in the 2x3 table of impairment level and ethnoracial group will be 800. This sample size will provide 90% power to reject the null hypothesis that the proportion of change is up to 30% using a two-sided test of level 0.0083, if the true proportion is 37% or higher. This calculation assumes unusable or missing data in 10% of participants.

8.2.2 Secondary Objectives

1) In each ethnoracial subgroup, to compare the proportions of participants with a hospitalization or ED visit within 12 months, between amyloid PET-positive and amyloid PET-negative participants in the study.

We hypothesize that, within each ethnoracial sub-group, positive amyloid PET will be associated with lower proportions of participants with hospitalizations or ED visits over 12 months.

Analysis strategy: For each ethnoracial subgroup the probability of a hospitalization or ED visit within the next 12 months will be compared between amyloid PET positive and negative participants using a logistic model. Separate analyses will be conducted for hospitalization and for ED visit. The analysis will be focused on the three major ethnoracial groups in the study: Black/African American, Latinx/Hispanic and White/Caucasian. In an exploratory analysis, we will examine change in each of the three primary components of the patient management composite as mediators in the relation between test result and 12-month hospitalization or 12-month ED visit. The analytic strategy will be similar to the mediation analysis described in Aim 1 above (section 8.1.3).

2) In each ethnoracial subgroup, to compare the proportions of participants with preventable hospitalizations over 12 months, between amyloid PET-positive and amyloid PET-negative participants in the study.

We hypothesize that, within each ethnoracial subgroup, positive amyloid PET will be associated with lower proportions of participants with preventable hospitalizations over 12 months. The analysis will be focused on the three major ethnoracial groups in the study: Black/African American, Latinx/Hispanic and White/Caucasian.

Analysis strategy: For each ethnoracial subgroup the probability of preventable hospitalization within the next 12 months will be compared between amyloid PET positive and negative participants using a logistic model. In an exploratory analysis, we will examine change in each of the three primary components of the patient management composite as mediators in the relation between test result and 12-month preventable hospitalization. The analytic strategy will be similar to the mediation analysis described in Aim 1 above (section 8.1.3).

8.2.3 Exploratory Objectives

1) To assess and compare the prevalence of amyloid PET positivity in Blacks/African-Americans and Latinx/Hispanics to Whites/Caucasians, separately for MCI and dementia.

We hypothesize that Blacks/African-Americans and Latinx/Hispanics, in comparison with Whites/Caucasians, will have a lower proportion of amyloid PET positivity at both the MCI and dementia stage. This is driven by the hypothesis that, in minority populations, non-AD processes, in particular cerebrovascular disease (with negative amyloid PET), will have a greater attributable risk for MCI and dementia, and AD (amyloid PET positive) will have a lower attributable risk compared to Whites/Caucasians. This hypothesis is supported by preliminary data from the first IDEAS Study.

Analysis strategy: The analysis for this exploratory objectives will use the entire study cohort to develop estimates of the prevalence of amyloid test positivity separately for each cell in a 3x2 table defined by impairment level (MCI or dementia) and ethnic/racial subgroups (Black/African-American or Latinx/Hispanic or White/Caucasian). Wilson confidence intervals will be reported. The estimates of prevalence and predictive value for Black/African-Americans and for Latinx/Hispanics will be compared to those for Whites/Caucasians using two-sided likelihood ratio tests. This analysis will be further elaborated via logistic regression modeling for the test positivity response. Covariates will include ethnic/minority status (expressed via two binary indicators, with White/Caucasian as the reference group), age and other variables of relevance. The model will be used to examine the effects of the covariates for ethnic/ minority status and their interaction. The results of the exploratory analyses will be presented as hypothesis generating findings and will not be adjusted for multiplicity of inferences.

2) To assess the positive and negative predictive values of ApoE4 for amyloid positivity in Blacks/African Americans and Latinx/Hispanics to Whites/Caucasians.

Based on existing literature, we further hypothesize that ApoE4 will be less predictive of amyloid PET positivity in Blacks/African Americans and Latinx/Hispanics compared to Whites/Caucasians.

Analysis strategy: The analysis for this objective will use the entire study cohort to develop estimates of the positive predictive value and negative predictive value of ApoE4 for amyloid positivity. These estimates will be developed separately for each cell in a 3x2 table defined by impairment level (MCI or dementia) and ethnic/racial subgroups (Black/African-American or Latinx/Hispanic or White/Caucasian).

3) To assess and compare the level of cognitive impairment (proportion of MCI vs. dementia) at study entry (pre-PET visit) in Blacks/African Americans and Latinx/Hispanics vs. Whites/Caucasians.

4) To assess and compare the use of AD medications at study entry (pre-PET visit) in Blacks/African Americans and Latinx/Hispanics vs. Whites/Caucasians.

We hypothesize that, because of disparities in access to specialist care, socioeconomic factors and cultural differences in recognition of dementia, Blacks/African Americans and Latinx/Hispanics will present to the study at a later stage of cognitive impairment (higher proportion of dementia vs. MCI), and show lower baseline use of AD medications at the pre-PET visit. All initial analyses will be based on self-identified race/ethnicity, but collection and future analysis of DNA may allow a re-analysis in the future based on admixture data.

Analysis strategy: The analysis for exploratory objectives (3) and (4) will use the entire study cohort to develop estimates of the prevalence of dementia and the proportion of participants using AD medications at study entry separately in each cell in a 3x2 table defined by impairment level (MCI or dementia) and ethnic/racial subgroups (Black/African-American or Latinx/Hispanic or White/Caucasian). Wilson confidence intervals will be reported. This analysis will be further elaborated via logistic regression modeling for each response. Covariates will include ethnic/minority status (expressed via two binary indicators, with White/Caucasian as the reference group), age and other variables of relevance to the particular response. The model will be used to examine the effects of the covariates for ethnic/ minority status and their interaction. The results of the exploratory analyses will be presented as hypothesis generating findings and will not be adjusted for multiplicity of inferences.

8.3 Aim 3

Aim 3: To describe the association of amyloid PET findings with changes in management and 12-month claims-derived health outcomes in individuals presenting with typical (progressive amnestic) versus atypical clinical presentations of MCI and AD dementia.

8.3.1 Primary Objectives

To assess and compare the proportion of change in the composite management endpoint in patients with "typical" (progressive amnestic) versus atypical clinical presentations of AD, separately for MCI and dementia.

We hypothesize that the frequency of 90-day post-PET changes in the management composite endpoint will be ≥ 30% in patients with both typical and atypical presentations of AD.

Analysis strategy: In this aim we will use the data from the entire study cohort of 7,000 participants. The analysis for the primary objective will compare the proportion of change in overall management between typical and atypical participants within the MCI and dementia subsets. To account for the two simultaneous comparisons, tests will be conducted at level 0.025 (two-sided).

Sample size considerations: Assuming that the ratio of MCI to dementia is between 60/40 and 40/60, the smallest sample size in a single subgroup (MCI or dementia) will be 2800 participants. Assuming further that data will be incomplete in up to 10% of all participants the effective minimum sample size in a subgroup will be 2520. This sample size will provide power at least 80% to detect a difference (excess) of 6% in the rate of change in the typical group, and at least 90% to detect a difference of 7% or higher. This computation assumes that the proportion of

change in the atypical cases will be 30% or higher and a 50/50 ratio of typical vs atypical cases. If the ratio is higher in favor of the typical cases, for example 60/40 or 70/30, the power will be slightly lower but will continue to exceed 90% for a difference of at least 7% in the proportion of change in the typical group compared to the atypical group.

8.3.2 Secondary Objectives

1) To assess and compare the proportions of participants with a hospitalization within 12 months, between amyloid PET-positive and amyloid PET-negative participants in the study, separately for patients with typical versus atypical clinical presentations of AD.

We hypothesize that positive amyloid PET will be associated with lower proportions of participants with hospitalizations over 12 months in patients with both typical and atypical clinical presentations of AD.

Analysis strategy: We will use claims data to estimate the proportions of participants with 12-month hospitalization within the subgroups defined by PET result and clinical presentation. We will compare the PET+ and PET- proportions using logistic models with the indicator for PET result as an independent variable. This analysis will be carried out separately for patients with typical and atypical clinical presentation. In an exploratory analysis, we will examine change in each of the three primary components of the patient management composite as mediators in the relation between test result and 12-month hospitalization. The analytic strategy will be similar to the mediation analysis described in Aim 1 above (section 8.1.3).

2) To assess and compare rates of 12 month hospitalizations, ED visits, preventable hospitalizations and overall resource utilization between patients with typical versus atypical clinical presentations of AD.

We hypothesize that positive amyloid PET will be associated with lower proportions of participants with 12 month hospitalizations, ED visits, preventable hospitalizations and overall resource utilization.

Analysis strategy: For binary endpoints (12-month hospitalization, ED visit and preventable hospitalization) we will estimate the corresponding proportions and compare them using logistic models with an indicator of typical or atypical presentation as an independent variable. For the analysis of overall resource utilization we will use linear mixed-model regression to compare total Medicare cost, after an appropriate transformation of the response variable.

3) To assess and compare the proportion of change in the composite management endpoint in patients with "typical" (progressive amnestic) versus atypical clinical presentations of AD, separately for MCI and dementia.

We hypothesize that amyloid PET will lead to ≥ 30% change between the pre-PET and post-PET composite management endpoint in patients with both "typical" and "atypical" clinical presentations. We further hypothesize that the proportion of change will be higher in atypical vs. typical cases.

Analysis strategy: The analysis will be similar to the previous objective, with the response variable being an indicator of change in composite management. The modeling will be done separately for participants with MCI and Dementia.

8.3.3 Exploratory Objective

To assess the association between change in the composite management primary endpoint and the following potential predictors and interactions:

- *Age (dichotomized at age 65)*
- *Level of cognitive impairment (MCI vs. dementia)*
- *Typical vs. atypical clinical presentation*
- *ApoE4 carriers vs. non-carriers*
- *Race / Ethnicity*
- *Medical co-morbidities (documented in pre-PET form)*

We hypothesize that early age-of-onset (<65), MCI level of cognitive impairment, atypical clinical presentations, ApoE4 non-carrier genetic status, racial/ethnic minority, and increased vascular co-morbidities will all be independently associated with greater change in management, controlling for all other variables in the model.

Analysis strategy: Logistic regression modeling will be used in this analysis. Covariates will include age at onset (dichotomized based on cut-off at age 65), indicator of level of cognitive impairment (MCI vs dementia), indicator of clinical presentation (typical vs atypical), indicator of ApoE4 carrier status, set of binary indicators representing combinations of race / ethnicity, and indicators of the presence of medical co-morbidities (as documented in pre-PET form). Interactions of interest will also be included in the model.

9.0 REFERENCES

1. Rabinovici GD, Gatsonis C, Apgar C, Chaudhary K, Gareen I, Hanna L, et al. Association of Amyloid Positron Emission Tomography With Subsequent Change in Clinical Management Among Medicare Beneficiaries With Mild Cognitive Impairment or Dementia. *JAMA.* 2019;321(13):1286–1294.
2. Hyman BT, Phelps CH, Beach TG, Bigio EH, Cairns NJ, Carrillo MC, et al. National Institute on Aging-Alzheimer's Association guidelines for the neuropathologic assessment of Alzheimer's disease. *Alzheimers Dement.* 2012 Jan;8(1):1-13.
3. Mirra, S.S., Heyman, A., McKeel, D., Sumi, S.M., Crain, B.J., Brownlee, L.M. et al. The Consortium to Establish a Registry for Alzheimer's Disease (CERAD). Part II. Standardization of the neuropathologic assessment of Alzheimer's disease. *Neurology.* 1991; 41: 479–486
4. Clark CM, Pontecorvo MJ, Beach TG, Bedell BJ, Coleman RE, Doraiswamy PM, et al. Cerebral PET with florbetapir compared with neuropathology at autopsy for detection of neuritic amyloid-β plaques: a prospective cohort study. *Lancet Neurol.* 2012 Aug;11(8):669-78. Epub 2012 Jun 28.
5. Curtis C, Gamez JE, Singh U, Sadowsky CH, Villena T, Sabbagh MN, et al. Phase 3 Trial of Flutemetamol Labeled With Radioactive Fluorine 18 Imaging and Neuritic Plaque Density. *JAMA Neurol*. 2015; 72(3):287-294.
6. Sabri O, Sabbagh MN, Seibyl J, Barthel H, Akatsu H, Ouchi Y, et al. Florbetaben PET imaging to detect amyloid beta plaques in Alzheimer's disease: phase 3 study. *Alzheimers Dement.* 2015 Aug;11(8):964-74. Epub 2015 Mar 28.
7. La Joie R, Ayakta N, Seeley WW, et al. Multisite study of the relationships between antemortem [11C]PIB-PET Centiloid values and postmortem measures of Alzheimer's disease neuropathology. *Alzheimers Dement.* 2019 Feb;15(2):205-216.. Epub 2018 Oct 19.
8. Lesman-Segev OH, et al. Abstract at Alzheimer's Association International Conference (AAIC) 2018. *Manuscript under review*
9. Mayeda ER, Glymour MM, Quesenberry CP, Whitmer RA. Inequalities in dementia incidence between six racial and ethnic groups over 14 years. *Alzheimers Dement.* 2016;12(3):216-224.
10. Filshtein TJ, Dugger BN, Jin LW, et al. Neuropathological Diagnoses of Demented Hispanic, Black, and Non-Hispanic White Decedents Seen at an Alzheimer's Disease Center. *Alzheimers Dis.* 2019;68(1):145-158.
11. Greiner KA, Friedman DB, Adams SA, et al. Effective recruitment strategies and community-based participatory research: community networks program centers' recruitment in cancer prevention studies. *Cancer Epidemiol Biomarkers Prev.* 2014;23(3):416-423.
12. De las Nueces D, Hacker K, DiGirolamo A, Hicks LS. A systematic review of community-based participatory research to enhance clinical trials in racial and ethnic minority groups. *Health Serv Res.* 2012;47(3 Pt 2):1363-1386.
13. Beach TG, Monsell SE, Phillips LE, Kukull W. Accuracy of the clinical diagnosis of Alzheimer disease at National Institute on Aging Alzheimer Disease Centers, 2005-2010. *J Neuropathol Exp Neurol.* 2012;71(4):266-273.

14. Monsell SE, Kukull WA, Roher AE, et al. Characterizing Apolipoprotein E epsilon4 Carriers and Noncarriers With the Clinical Diagnosis of Mild to Moderate Alzheimer Dementia and Minimal beta-Amyloid Peptide Plaques. *JAMA Neurol.* 2015;72(10):1124-1131.
15. Liu E, Schmidt ME, Margolin R, et al. Amyloid-beta 11C-PiB-PET imaging results from 2 randomized bapineuzumab phase 3 AD trials. *Neurology*. 2015;85(8):692-700.
16. Landau SM, Horng A, Fero A, Jagust WJ, Alzheimer's Disease Neuroimaging I. Amyloid negativity in patients with clinically diagnosed Alzheimer disease and MCI. *Neurology.* 2016;86(15):1377-1385.
17. Imaging Dementia—Evidence for Amyloid Scanning (IDEAS) Study protocol, 2015. ClinicalTrials.gov Identifier: NCT02420756.
18. Possin KL, Merrilees JJ, Dulaney S, et al. Effect of Collaborative Dementia Care via Telephone and Internet on Quality of Life, Caregiver Well-being, and Health Care Use: The Care Ecosystem Randomized Clinical Trial. *JAMA Intern Med.* 2019 Sep 30.
19. Bynum JP, Rabins PV, Weller W, Niefeld M, Anderson GF, Wu AW. The relationship between a dementia diagnosis, chronic illness, medicare expenditures, and hospital use. *J Am Geriatr Soc.* 2004;52(2):187-194.
20. Schwarzkopf L, Menn P, Leidl R, et al. Excess costs of dementia disorders and the role of age and gender - an analysis of German health and long-term care insurance claims data. *BMC Health Serv Res.* 2012;12:165.
21. Lyketsos CG, Sheppard JM, Rabins PV. Dementia in elderly persons in a general hospital. *Am J Psychiatry*. 2000;157(5):704-707.
22. Phelan EA, Borson S, Grothaus L, Balch S, Larson EB. Association of incident dementia with hospitalizations. *JAMA*. 2012;307(2):165-172.
23. Toot S, Devine M, Akporobaro A, Orrell M. Causes of hospital admission for people with dementia: a systematic review and meta-analysis. *J Am Med Dir Assoc*. 2013;14(7):463-470.
24. Gurland BJ, Wilder DE, Lantigua R, et al. Rates of dementia in three ethnoracial groups. *Int J Geriatr Psychiatry.* 1999;14(6):481-493.
25. Jansen WJ, Ossenkoppele R, Tijms BM, et al. Association of Cerebral Amyloid-beta Aggregation With Cognitive Functioning in Persons Without Dementia. *JAMA Psychiatry*. 2018;75(1):84-95.
26. Ossenkoppele R, Jansen WJ, Rabinovici GD, et al. Prevalence of amyloid PET positivity in dementia syndromes: a meta-analysis. *JAMA.* 2015;313(19):1939-1949.
27. Fillenbaum GG, Heyman A, Huber MS, et al. The prevalence and 3-year incidence of dementia in older Black and White community residents. *J Clin Epidemiol.* 1998;51(7):587-595.
28. Katz MJ, Lipton RB, Hall CB, et al. Age-specific and sex-specific prevalence and incidence of mild cognitive impairment, dementia, and Alzheimer dementia in blacks and whites: a report from the Einstein Aging Study. *Alzheimer Dis Assoc Disord.* 2012;26(4):335-343.
29. Perkins P, Annegers JF, Doody RS, Cooke N, Aday L, Vernon SW. Incidence and prevalence of dementia in a multiethnic cohort of municipal retirees. *Neurology*. 1997;49(1):44-50.
30. Tang MX, Cross P, Andrews H, et al. Incidence of AD in African-Americans, Caribbean Hispanics, and Caucasians in northern Manhattan. *Neurology*.

2001;56(1):49-56.

31. Sachs-Ericsson N, Blazer DG. Racial differences in cognitive decline in a sample of community-dwelling older adults: the mediating role of education and literacy. *Am J Geriatr Psychiatry*. 2005;13(11):968-975.
32. Haan MN, Mungas DM, Gonzalez HM, Ortiz TA, Acharya A, Jagust WJ. Prevalence of dementia in older latinos: the influence of type 2 diabetes mellitus, stroke and genetic factors. *J Am Geriatr Soc*. 2003;51(2):169-177.
33. Sosa-Ortiz AL, Acosta-Castillo I, Prince MJ. Epidemiology of dementias and Alzheimer's disease. *Arch Med Res*. 2012;43(8):600-608.
34. Howard G, Lackland DT, Kleindorfer DO, et al. Racial differences in the impact of elevated systolic blood pressure on stroke risk. *JAMA Intern Med.* 2013;173(1):46-51.
35. Howard G, Cushman M, Howard VJ, et al. Risk factors for intracerebral hemorrhage: the REasons for geographic and racial differences in stroke (REGARDS) study. *Stroke.* 2013;44(5):1282-1287.
36. Cushman M, McClure LA, Howard VJ, Jenny NS, Lakoski SG, Howard G. Implications of increased C-reactive protein for cardiovascular risk stratification in black and white men and women in the US. *Clin Chem.* 2009;55(9):1627-1636.
37. Evans DA, Bennett DA, Wilson RS, et al. Incidence of Alzheimer disease in a biracial urban community: relation to apolipoprotein E allele status. *Arch Neurol.* 2003;60(2):185-189.
38. Evans DA, Beckett LA, Field TS, et al. Apolipoprotein E epsilon4 and incidence of Alzheimer disease in a community population of older persons. *JAMA.* 1997;277(10):822-824.
39. Tang MX, Stern Y, Marder K, et al. The APOE-epsilon4 allele and the risk of Alzheimer disease among African Americans, whites, and Hispanics. *JAMA.* 1998;279(10):751-755.
40. Tycko B, Lee JH, Ciappa A, et al. APOE and APOC1 promoter polymorphisms and the risk of Alzheimer disease in African American and Caribbean Hispanic individuals. *Arch Neurol.* 2004;61(9):1434-1439.
41. Marden JR, Walter S, Tchetgen Tchetgen EJ, Kawachi I, Glymour MM. Validation of a polygenic risk score for dementia in black and white individuals. *Brain Behav.* 2014;4(5):687-697.
42. de la Monte SM, Hutchins GM, Moore GW. Racial differences in the etiology of dementia and frequency of Alzheimer lesions in the brain. *J Natl Med Assoc.* 1989;81(6):644-652.
43. Riudavets MA, Rubio A, Cox C, Rudow G, Fowler D, Troncoso JC. The prevalence of Alzheimer neuropathologic lesions is similar in blacks and whites. *J Neuropathol Exp Neurol.* 2006;65(12):1143-1148.
44. Sandberg G, Stewart W, Smialek J, Troncoso JC. The prevalence of the neuropathological lesions of Alzheimer's disease is independent of race and gender. *Neurobiol Aging.* 2001;22(2):169-175.
45. Wilkins CH, Grant EA, Schmitt SE, McKeel DW, Morris JC. The neuropathology of Alzheimer disease in African American and white individuals. *Arch Neurol.* 2006;63(1):87-90.
46. Schlesinger D, Grinberg LT, Alba JG, et al. African ancestry protects against Alzheimer's disease-related neuropathology. *Mol Psychiatry.* 2013;18(1):79-85.

47. Shibata D, Tillin T, Beauchamp N, et al. African Caribbeans have greater subclinical cerebrovascular disease than Europeans: this is associated with both their elevated resting and ambulatory blood pressure and their hyperglycaemia. *J Hypertens.* 2013;31(12):2391-2399
48. Albert MS, DeKosky ST, Dickson D, et al. The diagnosis of mild cognitive impairment due to Alzheimer's disease: recommendations from the National Institute on Aging-Alzheimer's Association workgroups on diagnostic guidelines for Alzheimer's disease. *Alzheimers Dement.* 2011;7(3):270-279.
49. McKhann GM, Knopman DS, Chertkow H, et al. The diagnosis of dementia due to Alzheimer's disease: Recommendations from the National Institute on Aging-Alzheimer's Association workgroups on diagnostic guidelines for Alzheimer's disease. *Alzheimers Dement.* 2011;7(3):263-269
50. de Wilde A, van der Flier WM, Pelkmans W, et al. Association of Amyloid Positron Emission Tomography With Changes in Diagnosis and Patient Treatment in an Unselected Memory Clinic Cohort: The ABIDE Project. *JAMA Neurol*. 2018;75(9):1062-1070.
51. Johnson KA, Minoshima S, Bohnen NI, et al. Appropriate use criteria for amyloid PET: a report of the Amyloid Imaging Task Force, the Society of Nuclear Medicine and Molecular Imaging, and the Alzheimer’s Association. Dual publication in: *Alzheimers Dement*. 2013;9:e-1-16 and *J Nucl Med*. 2013;54:476–490.
52. Mendez MF. The accurate diagnosis of early-onset dementia. *Int J Psychiatry Med*. 2006;36(4):401-12.
53. Johnson DK, Wilkins CH, Morris JC. Accelerated weight loss may precede diagnosis in Alzheimer disease. *Arch Neurol.* 2006;63(9):1312-17.
54. Sheline YI, Mintun MA, Barch DM, Wilkins C, Snyder AZ, Moerlein SM. Decreased hippocampal 5-HT(2A) receptor binding in older depressed patients using [18F]altanserin positron emission tomography. *Neuropsychopharmacology.* 2004;29(12):2235-2241.
55. Wilkins CH, Roe CM, Morris JC, Galvin JE. Mild physical impairment predicts future diagnosis of dementia of the Alzheimer's type. *J Am Geriatr Soc.* 2013;61(7):1055-1059.
56. Wilkins CH, Wilkins KL, Meisel M, Depke M, Williams J, Edwards DF. Dementia undiagnosed in poor older adults with functional impairment. *J Am Geriatr Soc.* 2007;55(11):1771-1776.
57. Dilworth-Anderson P, Cohen MD. Beyond diversity to inclusion: recruitment and retention of diverse groups in Alzheimer research. *Alzheimer Dis Assoc Disord.* 2010;24 Suppl:S14-18.
58. Dilworth-Anderson P, Thaker S, Burke JM. Recruitment strategies for studying dementia in later life among diverse cultural groups. *Alzheimer Dis Assoc Disord.* 2005;19(4):256-260.
59. Dilworth-Anderson, P. Introduction to the Science of Recruitment and Retention Among Ethnically Diverse Populations. *Gerontologist.* 2011:51(Suppl 1): S1–S4.
60. Moon H, Townsend AL, Dilworth-Anderson P, Whitlatch CJ. Predictors of Discrepancy Between Care Recipients With Mild-to-Moderate Dementia and Their Caregivers on Perceptions of the Care Recipients' Quality of Life. *Am J Alzheimers Dis Other Demen.* 2016;31(6):508-515.

61. Jack CR, Jr., Bennett DA, Blennow K, Carrillo MC, Dunn B, Haeberlein SB. NIA-AA Research Framework: Toward a biological definition of Alzheimer's disease. *Alzheimers Dement*. 2018 Apr; 14(4): 535–562.
62. Shen HN, Lu CL, Li CY. Dementia increases the risks of acute organ dysfunction, severe sepsis and mortality in hospitalized older patients: a national population-based study. *PLoS One.* 2012;7(8):e42751.
63. Naylor MD, Karlawish JH, Arnold SE, et al. Advancing Alzheimer's disease diagnosis, treatment, and care: recommendations from the Ware Invitational Summit. *Alzheimers Dement.* 2012;8(5):445-452.
64. Ossenkoppele R, Pijnenburg YA, Perry DC, et al. The behavioural/dysexecutive variant of Alzheimer's disease: clinical, neuroimaging and pathological features. *Brain*. 2015;138(Pt 9):2732-2749.
65. Denicoff AM, McCaskill-Stevens W, Grubbs SS, et al. The National Cancer Institute–American Society of Clinical Oncology Cancer Trial Accrual Symposium: Summary and Recommendations. *J Oncol Pract.* 2013:9(6):267-76.
66. Skinner JS, Morgan A, Hernandez-Saucedo H, et al. Associations between Markers of Glucose and Insulin Function and Cognitive Function in Healthy African American Elders. *J Gerontol Geriat Res.* 2015;4(4):232.
67. Heerman WJ, Jackson N, Roumie CL, et al. Recruitment methods for survey research: Findings from the Mid-South Clinical Data Research Network. *Contemporary Clinical Trials.* 2017, 62:50-55.
68. Skinner JS, Duke L, Wilkins CH. Why Ethnogeriatrics Is Important. Cummings-Vaughn L, Cruz-Oliver DM, eds. *Ethnogeriatrics: Healthcare Needs of Diverse Populations.* Cham: Springer International Publishing; 2017:3-17.
69. Wilkins CH, Skinner JS, Boyer AP, Morrow-Howell N, Smith JM, Birge SJ. A Community-Based Collaborative Care Model to Improve Functional Health in Underserved Community-Dwelling Older Adults. *Journal of Aging and Health.* First published online Nov 15, 2017. Vol.31(3):379-396. Issue published March 1, 2019.
70. Wilkins CH, Sheline YI, Roe CM, Birge SJ, Morris JC. Vitamin D deficiency is associated with low mood and worse cognitive performance in older adults. *Am J Geriatr Psychiatry*. 2006;14(12):1032-1040.
71. Johnson DA, Joosten YA, Wilkins CH, Shibao CA. Case Study: Community Engagement and Clinical Trial Success: Outreach to African American Women. *Clin Transl Sci.* 2015;8(4):388-390.
72. Corbie-Smith G, Thomas SB, St. George DMM. Distrust, race, and research. *Arch Intern Med.* 2002;162(21):2458–63.
73. Canty Williams I, Corbie-Smith G. Investigator beliefs and reported success in recruiting minority participants. *Contemporary Clinical Trials*, 2006. Vol. 27(6):580 – 586.

Appendix I: Saliva and Blood Collection

Dr. Sid O'Bryant, Professor and Executive Director of the Institute for Translational Research at the University of North Texas and an expert on blood-based biomarkers, has been added to the New IDEAS Study team to spearhead the Biorepository initiative (plasma and DNA from those study participants who consent to blood collection). He will also oversee saliva collection (from all participants) for ApoE genotyping. Dr. O'Bryant has multiple, ongoing NIH-funded projects specifically designed to identify and validate blood-based biomarkers for clinical use in AD. Dr. Robert Rissman has been added as the Director of the New IDEAS Biorepository at the Alzheimer's Therapeutic Research Institute (ATRI) at the University of Southern California (USC) Keck School of Medicine. Additional experts will be added to the New IDEAS Biomarker Committee (Dr. O'Bryant, Chair). This Committee will review and evaluate the scientific merit of proposals to share New IDEAS samples with investigators developing genomic and plasma-based biomarkers of brain amyloidosis and AD. The New IDEAS Biorepository will be used for validation, and not discovery studies, with the specific goal of advancing blood-based biomarkers as a first step in determining which patients should undergo amyloid PET scans in clinical practice. Consent for such sample use in such studies will be obtained at the time of enrollment. The biostatistics team at Brown University will ensure analytic integrity of all studies utilizing New IDEAS bio-samples.

I. Saliva DNA Sample Collection for ApoE Genotyping

Saliva collection kits will be provided directly to the New IDEAS participants by ATRI. Participants will collect their saliva sample per the instructions provided within the Saliva Kit.

Instructions for the Participant:

1. Do not eat, drink, smoke, or use oral hygiene products for at least 10 minutes before you start the collection process. Rinse the mouth with water and discard. Please wait at least 5 minutes after this rinse to start the collection procedure.
2. After the five minutes have passed, open the package containing the Saliva DNA Collection Device. Place contents on a clean and dry surface.
3. Place lips over the opening of the Collection Tube Funnel. Collect saliva until the sample level reaches the red indicator line.
4. When collection has been completed, unscrew the Funnel from the Collection Tube by rotating the tube in the direction shown until the tube is completely detached from Funnel. Remove the Cap from the bottom of the Collection Tube and screw it tightly closed.

Additional Instructions for the Participant:

Each participant will be provided with an instructional kit for informational purposes. Next, the participant is to do the following:

1. Vigorously invert the vial 20 times to stabilize the sample. Place tubes in the shipment materials provided in the Saliva Collection Kit and store at ambient air until shipment to ATRI via USPS.
2. All saliva kit samples must be labelled using labels provided by the New IDEAS

Biorepository at ATRI.

II. Blood Collection Protocol (to be collected by Quest Diagnostics Laboratory or ExamOne)

Blood will only be collected from those participants who have consented to blood collection.

Blood Collection Kits will be shipped directly to the participant from the New IDEAS Biorepository. The Blood Collection kit may either A) be taken to a local Quest laboratory by the participant for collection of blood samples or B) be stored at the participant's residence until a date is scheduled for completion of at-home blood draw by ExamOne. All blood collection tubes, sharps and other phlebotomy supplies will be provided by Quest/ExamOne. All instructions for the participant and Quest/ExamOneemployee will be provided in the Blood Collection Kit as will all labeling supplies and materials required for shipment of blood samples to the New IDEAS Biorepository. When performing blood collection, the time of the subject's last meal should be recorded on the Laboratory Procedures Data Form.

New IDEAS study participants will be provided blood collection kits from ATRI. If consent is provided, the Blood Collection Kits will either A) be taken to a local Quest Laboratory or B) utilized by ExamOne at the participant's residence for an at-home blood draw for collection of plasma per the New IDEAS Biorepository Procedures Manual.

Instructions for the Quest Site and ExamOne phlebotomist:

1. Quest/ExamOne site will collect 4 EDTA plasma tubes (~ 40mL or 4 tablespoons) using Quest/ExamOne supplies and standard Quest/ExamOne blood collection protocols.
2. All sample tubes must be labelled for identification using Alzheimer's Therapeutic Research Institute (ATRI) labels.
3. Place labels on ALL collection tubes BEFORE sample collection. This should help to ensure the label properly adheres to the tube before exposure to moisture or different temperatures.
4. Tubes will be stored at ambient temperature until shipment the same day as collection.

Once received at ATRI:

1. Samples will be spun, aliquoted and frozen upon arrival. Plasma and buffy coat will be extracted and banked in the biorepository.
2. Samples will be stored and monitored until a research project has been approved for use of the New IDEAS Biorepository samples
3. Once a study has been approved, the New IDEAS Biorepository will provide the appropriate amount of plasma to the approved investigative team
4. The New IDEAS Biostatistical group will provide all relevant clinical information associated with the samples.

III. Sample Shipping and Tracking:

1. All samples (saliva and blood, if opted in) should be shipped the same day of collection through the ATRI provided Fed Ex account and tracking will occur online using the FedEx tracking number.
    a. Ship samples overnight (Monday-Thursday).
    b. Samples are not to be collected on Fridays.
2. The blood collection worksheets must be completed by the Quest site/ExamOne phlebotomist at the time of the blood collection.
3. Send samples priority overnight by Fed Ex to:

Robert Rissman, PhD
USC ATRI Keck School of Medicine
ATRI Biomarker Lab & Biorepository
6370 Nancy Ridge Drive, STE 112
San Diego, CA 92121
Phone: 858-260-8066
Fax: 858-450-4207
Email: biomarkersupport@atrihub.io

4. Please note the ATRI Biomarker Laboratory is closed for the following US holidays. Sites will be notified in advance of holiday closures.

MLK Day	President's Day	Memorial Day	Independence Day
Labor Day	Thanksgiving (Thursday and Friday)	Winter Recess (Closed Christmas Eve through New Year's Day)	

IV. Sample Quality:

Once the samples arrive at the New IDEAS Biorepository, they will be evaluated for quality based on condition and amount for each sample. The samples will be accounted for by the New IDEAS biorepository. If the plasma or buffy coat sample is not obtained for any reason, this must be recorded on the sample worksheet, including the reason the sample was not obtainable.

Appendix II: New IDEAS Oversight and Organization Structure

The New IDEAS organization structure has been modeled after other successful studies of this type, and attempts to provide the optimal balance between tight control and broad engagement. The combination of a Steering Committee (with associated subcommittees), Operations Center, and Stakeholder Group create an effective structure that will promote rapid completion of the study while ensuring maximum data integrity.

The New IDEAS structure builds upon that of the original IDEAS Study in which Dr. Maria Carrillo and the Alzheimer's Association formed the Amyloid Imaging CED Stakeholder Group, which has grown over time to represent a comprehensive group of constituents. The Stakeholders Group has provided a forum for community engagement to develop the study proposal and has stimulated participation by various leaders in Alzheimer's disease and imaging, to include: Alzheimer's Association, Society of Nuclear Medicine and Molecular Imaging (SNMMI), World Molecular Imaging Society (WMIS), radiopharmaceutical vendors, imaging vendors, Alzheimer's disease specialists, ethicists, and others.

This group invited National Oncologic PET Registry (NOPR) investigators and ACR to participate in the dialogue and then to serve as part of the project team to develop the IDEAS Study, leveraging the knowledge and experience of the NOPR research team. As this relationship has evolved, ACR has been positioned to serve as the operations center for the IDEAS Study. Industry interest and support for this initiative has been evident since the formation of the Stakeholders Group. Three vendors have received FDA approval for amyloid PET tracers (Eli Lilly and Company [Avid Radiopharmaceuticals, Inc.], GE Healthcare, and Life Molecular, LLC) and have been in discussion with three national distributors (PETNET Solutions, IBA RadioPharma Solutions, and Cardinal Health) to arrange for distribution of each agent nationally under the IDEAS Study. The vendors have disparate capabilities to produce and disseminate agent and likely will have unequal demand from the new IDEAS Study sites. As a result of these common and potentially competing interests, the vendors have agreed to work through the Medical Imaging and Technology Alliance (MITA) as a voice of consensus.

New IDEAS Project Team

The New IDEAS Project Team is ideally suited for the ongoing development and conduct of the New IDEAS Study. Project Team members include the Study PI, Dr. Gil Rabinovici, Professor of Neurology, Radiology and Biomedical Imaging at the University of California, San Francisco, a behavioral neurologist with research expertise in amyloid PET, Dr. Bruce Hillner from Virginia Commonwealth University and Dr. Barry Siegel from Washington University, the chair and co-chair, respectively, of the National Oncologic PET Registry (NOPR), Dr. Constantine Gatsonis, Chair of the Department of Biostatistics at Brown University and Chief Statistician of the NOPR and world-renowned expert in diagnostic imaging statistical methods and analysis, Dr. Rachel Whitmer, a Senior Scientist and Epidemiologist at UC-Davis and an expert on population-based studies of dementia risk factors and outcomes, and Dr. Maria Carrillo, Chief Science Officer of Medical & Scientific Relations for the Alzheimer's Association.

To assist with applying best practices in minority recruitment for New IDEAS, we have added Dr. Consuelo H. Wilkins, Vice President for Health Equity at Vanderbilt University Medical

Center, who holds faculty appointments in the departments of medicine at both Vanderbilt University Medical Center and Meharry Medical College, to the core study team as Co-Chair. Dr. Wilkins is the director of the Community Engaged Research Core in the Vanderbilt Institute for Clinical and Translational Science and is widely recognized for her work in implementing best practices for engaging patients and communities in research. She is principal investigator of a PCORI research award focused on understanding the impact of patient engagement on research through Community Engagement Studios, in which researchers receive direct input from representative groups to frame research questions, enhance study design, implement protocols, and disseminate research. Dr. Wilkins has substantial leadership experience in community and stakeholder engagement at the CTSA and national levels. Prior to her current role, Dr. Wilkins was a clinical investigator in the Knight Alzheimer's Disease Research Center at Washington University and studied cognitive impairment and early AD in African Americans.

We have also added Dr. Peggye Dilworth-Anderson to the core study team as Co-Chair to assist with implementing best practices and strategies to improve minority recruitment. Dr. Dilworth-Anderson is Professor of Health Policy and Management at the Gillings School of Global Public Health-University of North Carolina, Chapel Hill. Her work will support this trial on developing innovative and inclusive recruitment and retention strategies for diverse groups in Alzheimer's disease research. Her work will also inform how best to involve patients, family caregivers and family decision makers in the recruitment process. She will also bring years of leadership experience to the team (former president of Gerontological Society of America, former member the Medical Scientific Advisory Council of the Alzheimer's Association, and current member of the Global Council on Brain Health) to help build community relations and create partnerships with community organizations to enhance recruitment efforts in this trial.

Dr. Sid O'Bryant, Professor and Executive Director of the Institute for Translational Research at the University of North Texas and an expert on blood-based biomarkers, has been added to the New IDEAS Study team as a Co-Chair to spearhead the biorepository initiative. Dr. O'Bryant has multiple, ongoing NIH-funded projects specifically designed to identify and validate blood-based biomarkers for clinical use in AD. Dr. Robert Rissman, Associate Professor of Neurosciences at the University of California, San Diego (UCSD) and Adjunct Professor of Neurology at University of Southern California (USC) has also been added to the New IDEAS Study team as a Co-Chair to co-manage the biorepository initiative with Dr. O'Bryant.

The ACR CRI and Brown University Biostatistics and Data Management Center will monitor participant accrual and data submission.

New IDEAS Operation Center

The Operations Center for the New IDEAS Study will be based in the ACR Center for Research and Innovation (CRI) in Philadelphia, Pennsylvania. As the Operations Center, the ACR has complete oversight and responsibility for conducting the research initiative under an agreement with CMS to develop and manage the study under the CMS CED provision. In very general terms, these responsibilities include:

- Oversight and management of study documentation and regulatory compliance
- Recruiting and contracting with participating sites (dementia experts and PET facilities)

- Creation and management of the New IDEAS Study database; collection, transfer and archival of data
- Management of all funds, to include collection, accounting, and distribution of funds.

A simplified organization structure is presented in the following figure:

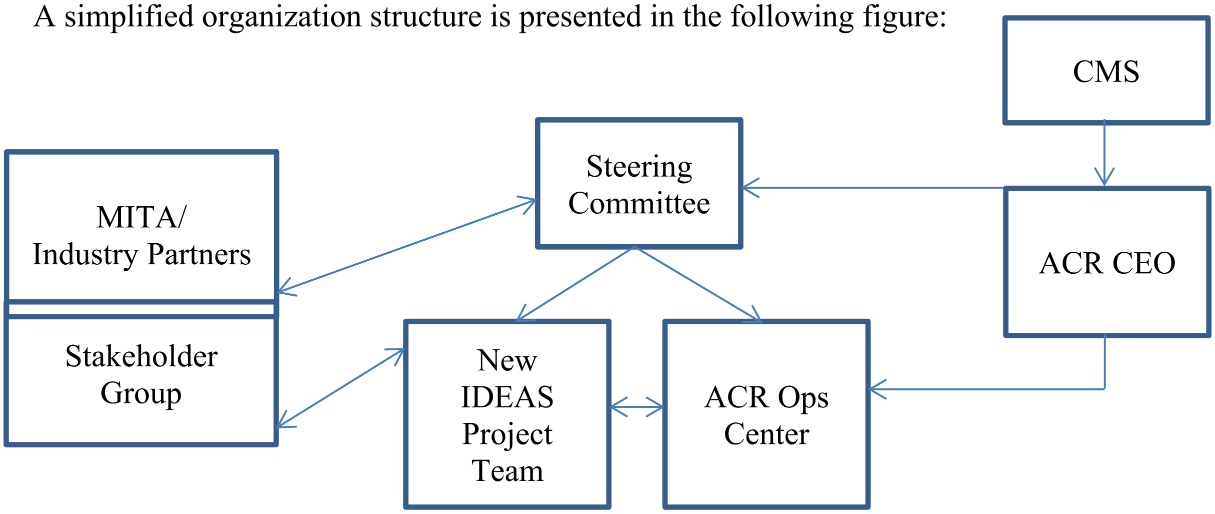

The daily activities undertaken within the Operations Center are guided by regulations and formal research guidelines, the clinical study protocol, and the leadership of the New IDEAS Study Project Team. The New IDEAS-Study Project will be governed by a Steering Committee. The New IDEAS Steering Committee will convene at least quarterly and will support the contract between CMS and ACR by monitoring study progress against established timelines, evaluating scientific program initiatives, ensuring publication and dissemination of data, and approving budgets while monitoring financial compliance thereof. The Steering Committee membership consists of:

- Chair
- ACR Representative
- Alzheimer's Association Representative
- CMS Representative
- Industry Representative (3)
- New IDEAS Project Team PI
- New IDEAS Project Team Statistician
- New IDEAS Project Team Co-chairs (6)
- New IDEAS Operations Center Director (non-voting)
- MITA Representative (non-voting)

Subcommittees will be created under the Steering Committee as needed to perform additional functional roles. It is expected that initial subcommittees will include a Publications and Data Access Committee and a Communications Committee.
