# Supplementary material for: Amyloid PET and clinical management in a diverse, cognitively impaired population: The New IDEAS Study
Source: Alzheimers Dement. 2025 Jul 29;21(7):e70504. doi: 10.1002/alz.70504 (PMC12305457; doi:10.1002/alz.70504)
Supplement: Supplementary file 2 — Supporting Information [file ALZ-21-e70504-s005.pdf]

NEW iDEAS

Imaging Dementia—Evidence For Amyloid Scanning

Dementia Specialist Practice Case Report Form Packet

Version 6 – September. 2023

Contents

Case Registration Form ....pages 2-5

Socio-demographics Form..pages 6-8

Pre-PET Clinical Assessment....................................pages 9-21

Post-PET Clinical Assessment...................................pages 22-38

Document Version History

Version #	Significant Changes	Section	Effective Date
1.0	Initial Launch of Case Report Forms	N/A	DECEMBER 2020
2.0	Inclusion of Covid-19 vaccination data<br>Code Table for Differential Diagnosis<br>Administrative updates	Case Registration<br>Pre-PET<br>Post-PET	JUNE 2021
3.0	Inclusion and Exclusion Criteria<br>Unknown vaccination status<br>Anti-amyloid (aducanumab) as pharma therapy	Case Registration<br>Pre-PET<br>Post-PET	AUGUST 2021
4.0	Supplemental Insurance Data<br>Date of Birth Verifications<br>Updated Exception Options	Case Registration<br>Pre-PET<br>Post-PET<br>Case Exception	December 2021
5.0	Inclusion Criteria	Case Registration	January 2023
6.0	Anti-amyloid (lecanemab) as pharma therapy	Pre-PET<br>Post-PET	September 2023

Case Registration Form

Instructions: This form is to be completed with each new referral. The cohort identification section must be ***self-reported by the participant****. All the assessments needed to determine eligibility are considered standard practice.*

Cohort Identification:

1. Date of birth: ______________________ [MM DD YYYY]

2. Patient's **self-reported** identification of their gender:
   ○ Male
   ○ Female
   ○ Transgender Male
   ○ Transgender Female
   ○ None of these fully describe me (allow free text)
   ○ Prefer not to answer

3. Patient's **self-reported** identification of their race:
   - ☐ American Indian or Alaska Native (For example: Aztec, Blackfeet Tribe, Mayan, Navajo Nation, Nome Eskimo Community)
   - ☐ Asian or Asian American (For example: Asian Indian, Chinese, Filipino, Japanese, Korean, Pakistani, Vietnamese)
   - ☐ Black, African American, or African (For example: African American, Ethiopian, Haitian, Jamaican, Nigerian, Somali)
   - ☐ Hispanic, Latino, or Spanish (For example: Colombian, Cuban, Dominican, Mexican or Mexican American, Puerto Rican, Salvadoran)
   - ☐ Middle Eastern or North African (For example: Algerian, Egyptian, Iranian, Lebanese, Moroccan, Syrian)
   - ☐ Native Hawaiian or other Pacific Islander (For example: Chamorro, Fijian, Marshallese, Native Hawaiian, Tongan)
   - ☐ White or European (For example: English, European, French, German, Irish, Italian, Polish)
   - ○ None of these fully describe me (allow free text)
   - ○ Prefer not to answer

Note: The study's accrual goals limit participation for certain patient cohorts. Below sections will not become available until cohort verification is submitted. An error message will occur if a participant slot is not available for identified cohort. If an error message is received, that patient is unfortunately unable to participate in the New IDEAS study.

Eligibility Confirmation:

All inclusion and exclusion criteria must be confirmed by the referring dementia specialist and/or the participant's medical records, prior to registration. I certify that all of the following are correct:

1. The patient is a Medicare beneficiary with Medicare as primary insurance	○ Yes ○ No
a. Specify beneficiary type:	○ Fee for service (traditional Medicare)<br>○ Medicare Advantage
b. Does the patient have supplemental or secondary insurance?	○ Yes ○ No
c. If yes, Name of plan:	________________________
2. The patient meets clinical criteria for Mild Cognitive Impairment (MCI) or Dementia as defined by the 2018 National Institute on Aging – Alzheimer's Association Research Framework.	○ Yes ○ No
3. The patient has had a brain MRI and/or CT within 24 months prior to enrollment.	○ Yes ○ No
4. The patient has had a clinical laboratory assessment (including complete blood count [CBC], comprehensive metabolic panel [CMP], TSH, vitamin B12) within 12 months prior to enrollment.	○ Yes ○ No
5. The patient is expected to be able to tolerate amyloid PET imaging as required by protocol, to be performed at a participating PET facility.	○ Yes ○ No
6. Neuropsychiatric syndrome can be classified into "clinically typical" or "clinically atypical" categories. (Refer to section 4.1.2 of protocol for guidance)	○ Yes ○ No
7. The Patient has signed consent to participate in the New IDEAS Study. Consent may be by proxy.	○ Yes ○ No
a. Consent provided by:	○ Patient ○ Proxy
b. In what language was the consent form completed	○ English ○ Spanish
c. Date consent signed: [MM DD YYYY]	____________________

The patient does not meet any of the exclusion criteria:

8. Normal cognition or subjective complaints that are not verified by cognitive testing.	□ verified
9. Knowledge of amyloid status, in the opinion of the referring dementia expert, may cause significant psychological harm or otherwise negatively impact the patient or family.	□ verified
10. Amyloid or tau status already known to patient or referring clinician based on prior imaging or CSF analysis.	□ verified
11. Previous amyloid PET scan obtained	□ verified
12. Current or previous treatment with an anti-amyloid agent.	□ verified
13. Current or previous enrollment in an anti-amyloid therapeutic trial.	□ verified
14. Scan is being ordered solely based on a family history of dementia, presence of Apo-lipoprotein E (APOE)□4, or in lieu of genotyping for suspected autosomal mutation carriers.	□ verified
15. Scan is being ordered for nonmedical purposes (e.g., legal, insurance coverage or employment screening).	□ verified
16. Cancer requiring active therapy (excluding non-melanoma skin cancer).	□ verified
17. Hip/pelvic fracture within the 12 months prior to enrollment.	□ verified
18. Body weight exceeds PET scanner weight limit.	□ verified
19. Currently pregnant or planning to become pregnant within 90 days of registration.	□ verified
20. Life expectancy less than 24 months based on medical co-morbidities.	□ verified
21. Residence in skilled nursing facility (assisted living facility is not an exclusion criterion).	□ verified

Optional Component Verification: Image archive, biorepository, and additional research studies:

The patient has consented to collection and archiving of his or her de-identified amyloid PET images for use in future research.	○ Yes ○ No
The patient has consented to collection and archiving of his or her de-identified blood samples for use in future research.	○ Yes ○ No
The IDEAS Study is collaborating with additional research studies investigating amyloid, cognitive decline, Alzheimer's disease and other types of dementia and the patient is willing to be contacted about other research studies for which he or she may be a candidate.	○ Yes ○ No

PATIENT INFORMATION:

Enter patient name as it appears on their Medicare ID card.

First Name: ________________________________

Middle Name: ______________________________

Last Name: ________________________________

Address: __________________________________

Address (line 2): ____________________________

City: _____________________________________

State: _____________________________________

Zip Code: __________________________________

Telephone (home): ___________ (cell) _________

Email: ____________________________________

SSN: _____________________________________

Medicare ID: ________________________________

(Provide traditional Medicare # for all participants, including those with Medicare Advantage, if known)

PATIENT INFORMED CONSENT: (**upload of consent mandatory for each registration**)

PET Facility where Amyloid PET has been scheduled: ______________________________________

Scan Date scheduled [MM DD YYYY]: ____

Name of Person responsible for the data on this form: __________________________________

Referring Dementia Expert: ____

Form submission date: ____

Socio-Demographics Form

Instructions: This form must be submitted within 7 days of the case registration date. Data elements below must be collected by authorized site staff ***during interview with participant****. All responses* ***must be self-reported by the participant****.*

---

1. Please specify marital status:
   - o Married
   - o Living with partner
   - o Widowed
   - o Divorced
   - o Separated
   - o Never married
   - o Prefer not to answer

2. Please specify living arrangements:
   - o Patient lives alone
   - o Patient lives with at least one other person

   With whom does patient live (check all that apply):
   - ☐ Spouse or partner
   - ☐ Child(ren)
   - ☐ Other relative
   - ☐ Caregiver/Household worker/Assisted living
   - ☐ Friend/Roommate
   - ☐ Someone else [free text] ______________________________________

3. Please specify the highest level of education you completed:
   - o No formal education
   - o Grade school - If yes, did you attend regularly?
     - o Yes, all year
     - o No, often missed school
   - o Attended high school but did not graduate - If yes, did you attend regularly?
     - o Yes, all year
     - o No, often missed school
   - o High school graduate - If yes, did you attend regularly?
     - o Yes, all year
     - o No, often missed school
   - o High school equivalence
   - o Some college or associate degree
   - o Bachelor's degree
   - o Master's degree
   - o Doctoral or professional degree

[Answer two questions below if response to Question 3 is "attended high school but did not graduate", or higher]

3a. Was your high school

- Private
- Public
- Taught at home

3b. Where was your high school located?

- Urban (inner city)
- Suburban
- Rural
- Outside the U.S.
- Don't recall
- Prefer not to answer

4. What is your current income?

- $0 - $4,999/year
- $5,000 - $9,999/year
- $10,000 - $14,999/year
- $15,000 - $19,999/year
- $20,000 - $24,999/year
- $25,000 - $29,999/year
- $30,000 - $34,999/year
- $35,000 - $49,999/year
- $50,000 - $74,999/year
- $75,000 and over/year
- Prefer not to answer

5. What was your income when you were 40 years old?

- $0 - $4,999/year
- $5,000 - $9,999/year
- $10,000 - $14,999/year
- $15,000 - $19,999/year
- $20,000 - $24,999/year
- Prefer not to answer
- $25,000 - $29,999/year
- $30,000 - $34,999/year
- $35,000 - $49,999/year
- $50,000 - $74,999/year
- $75,000 and over/year
- Do not recall

6. What is patient's primary (or preferred) language?

- English
- Spanish
- Other, specify ______________________________

[Answer question below if response to #6 is ‘Spanish’ or ‘Other’]

6a. How well do you speak your primary language?
- o Not at all
- o Not well
- o Well
- o Very well

7. How well do you speak English?
   - o Not at all
   - o Not well
   - o Well
   - o Very well

Name of Person responsible for the data on this form: ___________________________________

Form submission date: ____

Pre-PET Clinical Assessment Form

This form is intended to capture medical history data on your patient, as well as your diagnosis and management plan prior to amyloid PET. The management plan section asks that you describe your plan as if amyloid PET imaging were not available to your patient. This form must be submitted within 7 days of the patient's Pre-PET clinic visit.

Pre-PET Visit Status:

Was the Pre-PET visit completed?

Yes, I certify that the Pre-PET visit was completed. The Pre-PET form can be submitted.	No, the Pre-PET visit was not completed. I confirm that the Pre-PET form cannot be submitted.

Patient's Date of Birth [*MM/DD/YYY*]: ___________________________

Note: Verifying the patient's DOB serves as a safeguard to ensure the correct patient's data is being entered and submitted.

1. **Before patient can proceed to amyloid PET scan, Dementia Expert must certify that patient is aware of the ramifications of the test.**

I certify that I have discussed the medical and psychological ramifications of positive and negative amyloid scan results with the patient, family and caregivers, and they wish to proceed.	I have not discussed the medical and psychological ramifications of an amyloid scan. I understand that this makes the patient ineligible to proceed.

2. **Was this visit a face-to-face or VIDEO teleconference meeting between the treating physician and the patient?**
   - ○ Yes, face to face visit
   - ○ Yes, video telemedicine visit
   - ○ Yes, audio only telemedicine visit
   - ○ No
3. **Please specify the level of cognitive impairment:**
   - ○ Mild cognitive impairment
   - ○ Dementia

4. **Describe the patient's presentation of cognitive impairment:**

- Typical Presentation of Alzheimer's Disease **(all elements must apply)**
  - ☐ *Insidious onset. Symptoms have a gradual onset over months to years, not sudden over hours or days.*
  - ☐ *History of worsening of cognition by report or observation.*
  - ☐ *The initial and most prominent cognitive deficits are impairment in episodic memory (i.e., learning and recall of recently learned information). For a diagnosis of dementia, impairment in another cognitive domain (language, visuospatial, executive functions) is required.*
  - ☐ *The diagnosis of typical AD should not be applied when there is evidence of*
    - *(a) substantial concomitant cerebrovascular disease, a history of a stroke temporally related to the onset or worsening of cognitive impairment; or the presence of multiple or extensive infarcts or severe white matter hyperintensity burden; or*
    - *(b) core features of Parkinson's disease or dementia with Lewy bodies other than MCI or dementia;*
    - *(c) prominent features of behavioral variant frontotemporal dementia; or*
    - *(d) prominent features of semantic variant primary progressive aphasia or nonfluent/agrammatic variant primary progressive aphasia;*
    - *(e) evidence for another concurrent, active neurological disease, or a non-neurological medical comorbidity or use of medication that could have a substantial effect on cognition.*

OR

- Atypical for Alzheimer's disease. **(check all that apply)**
  - ☐ *The primary symptoms are not related to memory (e.g. primary deficits in executive functions, language, visuospatial, psychiatric or motor functions)**
  - ☐ *Presence of significant co-morbidities that can contribute to cognitive decline (e.g. medical conditions, pre-existing neurological or psychiatric conditions; substance abuse or other drug effects)*
  - ☐ *The course of clinical progression is atypical (i.e. not slowly and gradually progressive)*
  - ☐ *The clinical course has mixed features of AD and non-AD dementing illnesses (e.g. Parkinson's disease, Lewy body disease, frontotemporal dementia)*
    - ***Note**: Non-amnestic phenotypes associated with AD neuropathology, such as language-predominant presentation (also known as logopenic-variant primary progressive aphasia, visuospatial/visuoperceptual presentation (also known as posterior cortical atrophy and dysexecutive presentation (also known as frontal-variant AD should be included in the "clinically atypical" group.

COGNITIVE ASSESSMENTS

5. **Please enter MMSE and/or MoCA score at last clinical evaluation:**
   a. MMSE: ______
   b. MoCA: ______

   If score is 0, did the patient truly respond to each of the questions, or is score low because of non-compliance with testing?
   - o Truly low score
   - o Patient did not complete test or was otherwise non-compliant with test procedures

6. **Confirm that neither the patient's amyloid nor tau status is known to you or the patient:**
   - ☐ Patient has had no prior amyloid or tau imaging or results are not available
   - ☐ Patient has had no prior CSF testing for amyloid or tau, or previous testing was equivocal

7. **Year of onset of cognitive impairment:___ ___ ___ ___** ☐ **Year unknown:**

8. **Indicate diagnostic procedures that have been performed:**
   a. Confirm these required tests have been completed:
      - ☐ Basic laboratory work-up (complete metabolic panel, TSH, B12) within last 12 months (required)
      - ☐ Structural brain imaging (CT or MRI) within past 24 months (required)

   b. Indicate all of the following that have been done:
      - ☐ Neuropsychological testing
      - ☐ Additional serum laboratory tests (e.g., for infectious or autoimmune encephalopathies)
      - ☐ Genetic testing for Apolipoprotiein E genotyping
      - ☐ Genetic testing for autosomal dominant mutations associated with Alzheimer's disease (e.g., APP, PSEN1, PSEN2)
      - ☐ Genetic testing for autosomal dominant mutations associated with other dementia (e.g., mutations associated with Parkinson's disease, frontotemporal dementia, etc.)
      - ☐ Lumbar puncture for CSF studies excluding Alzheimer's disease CSF biomarkers (CSF Aβ42, total tau, phosphorylated tau)
      - ☐ FDG-PET
      - ☐ SPECT- Dopamine transporter (DaTscan)
      - ☐ SPECT- cerebral perfusion
      - ☐ Polysomnogram

9. **Please indicate whether the patient is currently taking the following Alzheimer's disease medications (Check all that apply):**
   - ☐ Cholinesterase inhibitor (e.g. donepezil, rivastigimine, galantamine)
   - ☐ Memantine

PATIENT MEDICAL HISTORY

10. **Please check all of the following items that are part of the patient's past or current medical history:**
    - o No clinically relevant medical history
    - o At least one condition is checked below *(Check all that apply):*
      - ☐ Congestive heart failure (with or without atrial fibrillation)
      - ☐ Atrial fibrillation
      - ☐ History of acute myocardial infarction
      - ☐ Ischemic heart disease (including angina pectoris and/or prior coronary artery angioplasty, stent or bypass grafting)
      - ☐ Hypertension
      - ☐ Dyslipidemia
      - ☐ Chronic kidney disease
      - ☐ Chronic obstructive pulmonary disease
      - ☐ Diabetes
      - ☐ Active depression
      - ☐ Bipolar affective disorder
      - ☐ Schizophrenia
      - ☐ Prior history of stroke and/or transient ischemic attack (TIA)

        Please indicate timing of stroke or TIA:
        - o Stroke or TIA occurred within past 24 months
        - o Stroke occurred more than 24 months ago
      - ☐ Cerebrovascular disease without stroke
      - ☐ Previous delirium
      - ☐ Epilepsy/seizure disorder
      - ☐ Parkinson's disease
      - ☐ Multiple sclerosis
      - ☐ Traumatic brain injury (TBI)

        Please indicate timing of TBI:
        - o TBI occurred within past 24 months
        - o TBI occurred more than 24 months ago
      - ☐ Tobacco use

        Please indicate timing of tobacco use:
        - o Past
        - o Current
      - ☐ Family history of dementia
        - o Family member diagnosed with Alzheimer's Disease
        - o Family member diagnosed with other or unknown type of dementia

11. Provide the following medical history specific to COVID-19

a. Has patient reported symptoms or suspicion of COVID-19 infection?

  ○ Yes ○ No

b. Has patient tested positive for SARS-CoV2 (by PCR and/or serology)?
  - Yes – I can verify a positive test in the medical record
  - Yes – patient/caregiver report a positive test, but I cannot verify in medical record
  - No – negative test reported by patient/caregiver or documented in medical record
  - No – patient has never been tested

[*Only answer questions 11c and 11d if answer is yes to either of the two questions above*]

c. If patient has had positive testing OR symptoms/suspicion of having COVID-19, what was the severity of their disease?
  - Asymptomatic
  - Mild-Moderate, symptoms controlled at home
  - Severe, hospitalized, but not ventilated
  - Severe, hospitalized and ventilated

d. Did patient experience any of the following neurologic conditions while infected with virus? Select all that apply.
  - ☐ Loss of smell and/or taste
  - ☐ Encephalopathy (e.g., delirium, psychosis)
  - ☐ Impaired consciousness
  - ☐ Increased cognitive impairment
  - ☐ Ischemic stroke
  - ☐ Hemorrhagic stroke
  - ☐ Seizure/s
  - ☐ Inflammatory central nervous system syndrome (e.g., meningitis, encephalitis, acute disseminating encephalomyelitis)
  - ☐ Inflammatory peripheral nervous system syndrome (e.g., Guillain-Barré syndrome, inflammatory neuropathy, radiculopathy or plexopathy)
  - ☐ Other neurologic symptoms, specify: ______________________
  - ☐ No neurologic manifestations reported

FOR CONSIDERATION: These other neurologic symptoms have been noted as possibly related to COVID infection: central nervous system (CNS) manifestations (dizziness, headache, impaired consciousness, acute cerebrovascular disease, ataxia, and seizure), peripheral nervous system (PNS) manifestations (taste impairment, smell impairment, vision impairment, and nerve pain).

e. Do you believe that COVID-19 is contributing to your patient's current cognitive complaint?
  - No
  - Yes - Psychosocial impact of COVID-19 are contributing.
  - Yes - The direct neurologic effects of the virus are contributing.
  - Yes - Both psychosocial and direct neurologic effects are contributing.

f. Has your patient been vaccinated against COVID-19?
   - No
   - Yes
     1. Which vaccine did they receive?
        - Pfizer-BioNTech
          - ☐ Single dose only
          - ☐ Fully vaccinated (two doses)
        - Moderna
          - ☐ Single dose only
          - ☐ Fully vaccinated (two doses)
        - Johnson & Johnson/Janssen
        - Unknown
   - Unknown (*Participant does not know vaccination status and EMR does not indicate vaccination status*)

     2. Date of final dose: ___________________ (MONTH/DAY/YEAR)
        - ☐ Date unknown

DIFFERENTIAL DIAGNOSIS

PRIORITIZE your differential diagnosis for the underlying cause of your patient's cognitive condition using this list of options. For your convenience you may view the entire list in a separate window or print a copy of it for reference.

- *Select the MOST likely etiologic cause of the condition.*
- *Then select up to 3, other differential diagnoses from this list, in order of likelihood.*

We have grouped the options by category, and alphabetized entries within category.

Code Table for Differential Diagnoses for Cause of Cognitive Impairment

DIFFERENTIAL DIAGNOSIS.

12. Please enter the MOST likely etiologic cause of cognitive impairment. For example, if the patient has MCI and you suspect Alzheimer's disease (AD) as the most likely underlying cause of MCI, list AD as your primary choice.

a. Primary differential diagnosis for cause of cognitive impairment

Complete list will pop up

b. Indicate your confidence in your primary diagnosis:

Not at all confident									Certain
1	2	3	4	5	6	7	8	9	10
○	○	○	○	○	○	○	○	○	○

13. Enter up to 3 additional differential diagnoses for this patient <u>in order of likelihood.</u>

a. Additional differential diagnosis — *Complete list will pop up*

   i. Do you wish to add another diagnosis?

      ○ Yes ○ No

b. Additional differential diagnosis (optional) — *Complete list will pop up*

   i. Do you wish to add another diagnosis?

      ○ Yes ○ No

c. Additional differential diagnosis (optional) — *Complete list will pop up*

14. Please rate your estimated likelihood that AD pathology is present and causing or contributing to cognitive symptoms:

Not at all confident									Certain
1	2	3	4	5	6	7	8	9	10
○	○	○	○	○	○	○	○	○	○

<u>MANAGEMENT PLAN</u>

INSTRUCTIONS:

Throughout this section, respond ASSUMING THAT YOUR PATIENT COULD NOT HAVE AN AMYLOID PET SCAN at any time in the near future.

The post-PET form, which will be due approximately 90 days after your patient has the amyloid PET scan, will ask which items <u>from this pre-PET management plan</u> have been implemented. Your selections on this form will drive the questions asked on the Post-PET form.

Non-pharmaceutical interventions include counseling, new testing or imaging, new referrals to specialists or to clinical trials for cognitive conditions. You may also specify other interventions.

Pharmaceutical interventions include drugs or vitamins to treat the complaint with which this patient presented.

15. **If your patient could not have an amyloid PET scan, what would your management plan be at this time?** *(Consider both pharmaceutical and non-pharmaceutical interventions when answering this first question in this section. If your participant is already taking drugs or vitamins specifically for cognitive impairment, select an option that allows you to complete the pharmaceutical section.)*

- ○ Watchful waiting only (i.e., The patient is not already taking drugs for cognition; I plan no drug additions or adjustments; and no new diagnostic tests, counselling or other referrals).
- ○ I would recommend both non-pharmaceutical and either new pharmaceutical interventions or my patient is already taking drugs for their cognitive condition. (*Select at least one option from Question 16a and at least one from 16b.*)
- ○ I would recommend non-pharmaceutical intervention(s), but no new drugs and my patient is not already taking drugs for cognitive impairment. (*Select at least one option from Question 16a but do not respond to Question 16b.*)
- ○ I would recommend new or modified pharmaceutical intervention(s), or my patient is already taking drugs for cognitive impairment. I do not recommend any new diagnostic tests, counselling or other referrals. (*Do not respond to Question 16a, but select at least one item from Question 16b.*)

16a. NON-PHARMACEUTICAL MANAGEMENT

**NON-PHARMACEUTICAL INTERVENTIONS**<br><br>*(See next table/questions 16b. for drug management)*	***16a. Would you recommend this action? For this question, you should assume that the patient DOES NOT HAVE ACCESS TO AMYLOID PET***
**Counseling for safety, planning & social support**	
Counseling about safety precautions (home safety, medication monitoring, driving)	☐ Recommend
Counseling about financial/medical decision making, advanced directives	☐ Recommend
Referral to community patient/caregiver support resources (e.g. social work, Alzheimer's Association, Family Caregiver Alliance, etc.)	☐ Recommend
**Additional diagnostic procedures**	
Neuropsychological testing referral	☐ Recommend

NON-PHARMACEUTICAL INTERVENTIONS<br><br>*(See next table/questions 16b. for drug management)*	***16a. Would you recommend this action? For this question, you should assume that the patient DOES NOT HAVE ACCESS TO AMYLOID PET***
**Imaging (brain/head)**	
CT/CTA with/without contrast	☐ Recommend
MRI/MRA with/without contrast	☐ Recommend
Brain FDG-PET	☐ Recommend
DaTscan (Parkinson's disease)	☐ Recommend
SPECT for regional cerebral perfusion	☐ Recommend
Tau PET	☐ Recommend
**Genetic tests**	
ApoE genotyping	☐ Recommend
Autosomal dominant mutations for AD	☐ Recommend
Autosomal dominant mutations for other conditions	☐ Recommend
**Other Laboratory testing or procedures (non-imaging)**	
Lumbar puncture:	
AD CSF biomarkers (CSF Aβ42, total tau, phosphorylated tau)	☐ Recommend
Other CSF studies	☐ Recommend
Serologic (RPR, HIV, auto-antibodies)	☐ Recommend
**Other Tests**	
EEG	☐ Recommend
Polysomnography	☐ Recommend
**Referral to other specialists for non-pharmacological interventions**	
Other specialist (e.g. psychiatrist, sleep medicine)	☐ Recommend
Surgical intervention (e.g. shunting for hydrocephalus)	☐ Recommend
Substance abuse treatment/support programs	☐ Recommend
Physical, occupational or speech therapy rehabilitation	☐ Recommend
Cognitive rehabilitation	☐ Recommend
**Clinical trial referral**	
Drug therapy or other therapeutic trial for AD (includes amyloid (+) MCI)	☐ Recommend
Drug therapy or other therapeutic trial for non-AD disorder (please specify)	☐ Recommend

NON-PHARMACEUTICAL INTERVENTIONS<br><br>*(See next table/questions 16b. for drug management)*	***16a. Would you recommend this action? For this question, you should assume that the patient DOES NOT HAVE ACCESS TO AMYLOID PET***
Referral to observational (non-interventional) research study	☐ Recommend

16b. PHARMACEUTICAL MANAGEMENT

INSTRUCTIONS:

a. ***ASSUMING THAT AMYLOID PET WERE NOT AVAILABLE, indicate all drugs that your patient is currently taking OR that you recommend starting at this time.***

b. ***For any drug your patient is already taking, and STILL ASSUMING THAT AMYLOID PET WERE NOT AVAILABLE, indicate your plan for managing that drug.***
"Continue" = Continue current drug and dosage
"Adjust" = Adjust dosage or change to another drug within the class

PHARMACEUTICAL INTERVENTIONS	16.b.i. ASSUMING THAT AMYLOID PET WERE NOT AVAILABLE, indicate all drugs that your patient is currently taking OR that you recommend starting at this time	16.b.ii. For any drug that your patient is already taking, and STILL ASSUMING THAT AMYLOID PET WERE NOT AVAILABLE, indicate your plan for managing this drug
**AD Drugs**		
Cholinesterase inhibitors (donepezil, rivastigmine, galantamine)	○ Currently taking<br>○ Recommend starting at this time	○ Continue<br>○ Adjust<br>○ Stop
Memantine	○ Currently taking<br>○ Recommend starting at this time	○ Continue<br>○ Adjust<br>○ Stop

PHARMACEUTICAL INTERVENTIONS	16.b.i. ASSUMING THAT AMYLOID PET WERE NOT AVAILABLE, indicate all drugs that your patient is currently taking OR that you recommend starting at this time	16.b.ii. For any drug that your patient is already taking, and STILL ASSUMING THAT AMYLOID PET WERE NOT AVAILABLE, indicate your plan for managing this drug
Anti-amyloid Therapeutic<br>○ Aducanumab<br>○ Lecanemab	○ Recommend starting at this time	
**Neuropsychiatric drugs impacting cognition**		
Anti-depressants, mood stabilizers	○ Currently taking<br>○ Recommend starting at this time	○ Continue<br>○ Adjust<br>○ Stop
Anti-psychotics	○ Currently taking<br>○ Recommend starting at this time	○ Continue<br>○ Adjust<br>○ Stop
Sedatives/sleep aids	○ Currently taking<br>○ Recommend starting at this time	○ Continue<br>○ Adjust<br>○ Stop
**Non-neuropsychiatric drugs impacting cognition**		
Anti-cholinergic drugs, opiates, muscle relaxants, etc.	○ Currently taking<br>○ Recommend starting at this time	○ Continue<br>○ Adjust<br>○ Stop
**Non-neurology/psychiatric pharmacologic therapies***		
Treatment for medical/vascular risk factors (e.g.; anti-platelets, anti-hypertensives, diabetes medications, lipid lowering drugs, etc.)	○ Currently taking<br>○ Recommend starting at this time	○ Continue<br>○ Adjust<br>○ Stop
**Other neurologic condition**		
Treatment for Parkinson's disease (e.g. carbidopa/levodopa, dopamine agonists, MAO-B inhibitors, others	○ Currently taking<br>○ Recommend starting at this time	○ Continue<br>○ Adjust<br>○ Stop

PHARMACEUTICAL INTERVENTIONS	16.b.i. ASSUMING THAT AMYLOID PET WERE NOT AVAILABLE, indicate all drugs that your patient is currently taking OR that you recommend starting at this time	16.b.ii. For any drug that your patient is already taking, and STILL ASSUMING THAT AMYLOID PET WERE NOT AVAILABLE, indicate your plan for managing this drug
Treatment for epilepsy (i.e. anti-epileptics)	○ Currently taking<br>○ Recommend starting at this time	○ Continue<br>○ Adjust<br>○ Stop
**Targeted therapies**		
Immunosuppressant (auto-immune/ inflammatory encephalopathy)	○ Currently taking<br>○ Recommend starting at this time	○ Continue<br>○ Adjust<br>○ Stop
Vitamin repletion (nutritional deficiency)	○ Currently taking<br>○ Recommend starting at this time	○ Continue<br>○ Adjust<br>○ Stop
Antimicrobials (infectious encephalopathy)	○ Currently taking<br>○ Recommend starting at this time	○ Continue<br>○ Adjust<br>○ Stop

CERTIFICATIONS

17. **All of the actions and prescriptions you planned are listed below. Please certify that this represents your complete management plan, <u>if you could not order an amyloid PET scan</u>.**

I certify that the list above is my complete management plan for this patient, assuming Amyloid PET were unavailable at this time.

I wish to make changes to my selections. Return to management plan questions.

Name of Person responsible for the data on this form: ___________________________________

Form submission date: ____

Post-PET Clinical Assessment Form

This form is used to record the revised diagnosis and actual management plan at 90 days post-PET clinical visit (allowable range is 60-120 days), now incorporating amyloid PET results. This form must be submitted within 30 days of the patient's Post-PET clinical visit.

Follow-Up Visit Status:

1. Was the follow-up visit completed?

Yes, I certify that the Post-PET visit was completed. The Post-PET form can be submitted.	No, the Post-PET visit was not completed. I confirm that the Post-PET form cannot be submitted.

Patient's Date of Birth [*MM/DD/YYY*]: ______________________________

Note: Verifying the patient's DOB serves as a safeguard to ensure the correct patient's data is being entered and submitted.

a. Date of clinic visit or patient contact: ____ / ____ / __________
Days since PET scan _________ (calculated by system)

b. If days since PET scan <60 or >120, indicate the reason(s) follow-up visit was not completed within the expected timeframe, and then complete the rest of the form:

- ☐ Patient or caregiver was unable to make arrangements to return within window
- ☐ Patient developed an intercurrent illness that prevented return within window
- ☐ Dementia specialist was unavailable within window
- ☐ Reason related to COVID-19 pandemic [Participant ill with COVID-19, participant quarantined (self- or government imposed), clinic or physician unavailable due to COVID restrictions or personal illness]
- ☐ Other, specify:____________________________

c. Was this follow-up visit a face-to-face or VIDEO teleconference meeting between the treating physician and the patient?

- ○ Yes, face to face
- ○ Yes, video telemedicine visit
- ○ Yes, audio only telemedicine visit
- ○ No

2. **Specify the results of the amyloid PET scan, as you understand them (select one):**
   - ○ Positive for cortical beta-amyloid
   - ○ Equivocal / Indeterminate for cortical beta amyloid
   - ○ Negative for cortical beta-amyloid
   - ○ Uninterpretable or technically inadequate study

3. **Did the patient, family or proxy report any adverse effects related to learning the amyloid scan result?**
   - ○ No *(Skip to question 4)*
   - ○ Yes *(Please describe the adverse effects of learning results of amyloid PET scan)*.

     ____

     ____

4. **Since the date of the PET scan, has this patient**
   a. Had any hospital admissions?
      - ○ Yes
      - ○ No

   b. Had any visits to an emergency room (in hospital or free standing, but not urgent care)?
      - ○ Yes
      - ○ No

DIFFERENTIAL DIAGNOSIS

PRIORITIZE your differential diagnosis for the underlying cause of your patient's cognitive condition using this list of options. For your convenience you may view the entire list in a separate window or print a copy of it for reference.

- *Select the MOST likely etiologic cause of the condition.*
- *Then select up to 3, other differential diagnoses from this list, in order of likelihood.*

We have grouped the options by category, and alphabetized entries within category.

Code Table for Differential Diagnoses for Cause of Cognitive Impairment

<u>DIFFERENTIAL DIAGNOSIS</u>.

5. **Please enter the <u>MOST likely etiologic cause</u> of cognitive impairment. For example, if the patient has MCI and you suspect Alzheimer's disease (AD) as the most likely underlying cause of MCI, list AD as your primary choice.**

   a. Primary differential diagnosis for cause of cognitive impairment — ***Complete list will pop up***

   b. Indicate your confidence in your primary diagnosis:

Not at all confident									Certain
1	2	3	4	5	6	7	8	9	10
○	○	○	○	○	○	○	○	○	○

6. **Enter up to 3 additional differential diagnoses for this patient <u>in order of likelihood.</u>**

   a. Additional differential diagnosis — ***Complete list will pop up***

      i. Do you wish to add another diagnosis?

      ○ Yes ○ No

   b. Additional differential diagnosis (optional) — ***Complete list will pop up***

      i. Do you wish to add another diagnosis?

      ○ Yes ○ No

   c. Additional differential diagnosis (optional) — ***Complete list will pop up***

7. **Please rate your estimated likelihood that AD pathology is present and causing or contributing to cognitive symptoms:**

Not at all confident									Certain
1	2	3	4	5	6	7	8	9	10
○	○	○	○	○	○	○	○	○	○

8. **<u>MANAGEMENT PLAN</u>**

This section consists of 5 parts.

PART 1: Overview of Management Plan

PART 2: Status of Non-Pharmaceutical Interventions recommended on the Pre-PET form.

PART 3: NEW Non-Pharmaceutical Interventions recommended since the PET scan.

PART 4: Status of Pharmaceutical Interventions recommended on the Pre-PET form.

PART 5: NEW Pharmaceutical Interventions recommended since the PET scan.

You will be reminded of your selections on the Pre-PET form before each Part. You will only be shown parts that are applicable based on your Pre-PET responses and answers you give in Part 1.

PART 1: OVERVIEW OF MANAGEMENT PLAN

THIS IS THE MANAGEMENT PLAN YOU REPORTED PRIOR TO THE AMYLOID PET SCAN.

Non-Pharmaceutical Interventions **Pharmaceutical Interventions**

The Electronic Data Collection System will present items in this section ADAPTIVELY, based on your responses on the Pre-PET form. Some questions will not be available if no response is appropriate given your Pre-PET Management Plan. If you are unable to access a section you wish to answer, please contact New IDEAS HQ for advice on how to do that.

[For options 1-4] **Watchful waiting was the plan you reported for this patient on the pre-PET form. Select the option from this list that matches your current plan.**

- [Option 1] Watchful waiting is still the plan. I have **NOT** recommended any **NEW** counselling, referrals to specialists or clinical trials for cognitive impairment, additional testing, or pharmaceutical therapy.
- [Option 2] Watchful waiting is no longer the plan. Since the PET scan, I have recommended **BOTH non-pharmaceutical and pharmaceutical interventions**.
- [Option 3] Watchful waiting is no longer the plan. Since the PET scan, **I have recommended non-pharmaceutical interventions** (counselling, referrals to specialists or clinical trials, or additional testing.) I have not recommended pharmaceutical intervention.
- [Option 4] Watchful waiting is no longer the plan. Since the PET scan, **I have recommended pharmaceutical interventions** (i.e., prescribed drugs or vitamins for cognitive condition) I have **not** recommended non-pharmaceutical interventions such as counselling, additional testing, referrals to specialists or referral to clinical trials.

[For options 5-8] You indicated at least one intervention, either non-pharmaceutical or pharmaceutical, on the Pre-PET form as your plan for managing this patient. **Have you ADDED any NEW interventions since the PET scan?**

- [Option 5] I have added **BOTH NEW non-pharmaceutical and NEW pharmaceutical interventions** to the management plan for this patient since the PET scan.
- [Option 6] I have added **NEW non-pharmaceutical interventions** to the management plan for this patient since the PET scan, but I have **NOT changed the plan for pharmaceutical management**.
- [Option 7] I have added **NEW pharmaceutical interventions** to the management plan for this patient since the PET scan, but I have **NOT added any non-pharmaceutical interventions** (e.g. referrals to specialists or clinical trials, additional tests, or counseling.)
- [Option 8] I have **NOT ADDED ANY NEW INTERVENTIONS** that were not part of the Pre-PET management plan for this patient.

8a. Did the amyloid PET results contribute significantly to this management plan?

- Yes
- No

PART 2: STATUS OF NON-PHARMACEUTICAL INTERVENTIONS SELECTED ON THE PRE-PET FORM

Instructions: Report the status of the non-pharmaceutical interventions you included in this patient's Pre-PET management plan. Complete EVERY ROW of this table, as each of the items shown is an intervention you selected on the Pre-PET form.

These are the items you selected on the Pre-PET form for **Non-Pharmaceutical Interventions**

Note: If there were no non-pharmaceutical interventions on the pre-PET form, a message appears that says "You did not select any non-pharmaceutical interventions on the pre-PET form. Therefore, Part 2 is omitted.

NON-PHARMACEUTICAL INTERVENTIONS	*8b. Status of interventions that were part of your Pre-PET management plan for this patient.*
**Counseling for safety, planning & social support**	
Counseling about safety precautions (home safety, medication monitoring, driving, whether to continue working)	○ Implemented<br>○ Completed as recommended on Pre-PET<br>○ Significantly modified from Pre-PET<br>○ Not implemented
Counseling about financial/medical decision making, advanced directives	○ Implemented<br>○ Completed as recommended on Pre-PET<br>○ Significantly modified from Pre-PET<br>○ Not implemented
Referral to community patient/caregiver support resources (e.g. social work, Alzheimer's Association, Family Caregiver Alliance, etc.)	○ Implemented<br>○ Completed as recommended on Pre-PET<br>○ Significantly modified from Pre-PET<br>○ Not implemented
**Additional diagnostic procedures**	
Neuropsychological testing referral	○ Implemented<br>○ Completed as recommended on Pre-PET<br>○ Significantly modified from Pre-PET<br>○ Not implemented
**Imaging (brain/head)**	
CT/CTA with/without contrast	○ Implemented<br>○ Completed as recommended on Pre-PET<br>○ Significantly modified from Pre-PET<br>○ Not implemented
MRI/MRA with/without contrast	○ Implemented<br>○ Completed as recommended on Pre-PET<br>○ Significantly modified from Pre-PET<br>○ Not implemented
Brain FDG-PET	○ Implemented<br>○ Completed as recommended on Pre-PET<br>○ Significantly modified from Pre-PET<br>○ Not implemented
DaTscan (Parkinson's disease)	○ Implemented<br>○ Completed as recommended on Pre-PET<br>○ Significantly modified from Pre-PET<br>○ Not implemented
SPECT for regional cerebral perfusion	○ Implemented<br>○ Completed as recommended on Pre-PET<br>○ Significantly modified from Pre-PET<br>○ Not implemented
Tau PET	○ Implemented<br>○ Completed as recommended on Pre-PET<br>○ Significantly modified from Pre-PET<br>○ Not implemented

**NON-PHARMACEUTICAL INTERVENTIONS**	***8b. Status of interventions that were part of your Pre-PET management plan for this patient.***
**Other Laboratory testing or procedures (non-imaging)**	
Lumbar puncture	
AD CSF biomarkers (CSF Aβ42, total tau, phosphorylated tau)	○ Implemented<br>○ Completed as recommended on Pre-PET<br>○ Significantly modified from Pre-PET<br>○ Not implemented
Other CSF studies	○ Implemented<br>○ Completed as recommended on Pre-PET<br>○ Significantly modified from Pre-PET<br>○ Not implemented
Serologic (RPR, HIV, auto-antibodies)	○ Implemented<br>○ Completed as recommended on Pre-PET<br>○ Significantly modified from Pre-PET<br>○ Not implemented
**Genetic tests**	
ApoE genotyping	○ Implemented<br>○ Completed as recommended on Pre-PET<br>○ Significantly modified from Pre-PET<br>○ Not implemented
Autosomal dominant mutations for AD	○ Implemented<br>○ Completed as recommended on Pre-PET<br>○ Significantly modified from Pre-PET<br>○ Not implemented
Autosomal dominant mutations for other conditions	○ Implemented<br>○ Completed as recommended on Pre-PET<br>○ Significantly modified from Pre-PET<br>○ Not implemented
**Other testing**	
EEG	○ Implemented<br>○ Completed as recommended on Pre-PET<br>○ Significantly modified from Pre-PET<br>○ Not implemented
Polysomnography	○ Implemented<br>○ Completed as recommended on Pre-PET<br>○ Significantly modified from Pre-PET<br>○ Not implemented
**Referral to other specialists for non-pharmacological interventions**	
Other specialist (e.g. psychiatrist, sleep medicine)	○ Implemented<br>○ Completed as recommended on Pre-PET<br>○ Significantly modified from Pre-PET<br>○ Not implemented

NON-PHARMACEUTICAL INTERVENTIONS	*8b. Status of interventions that were part of your Pre-PET management plan for this patient.*
Surgical intervention (e.g. shunting for hydrocephalus)	○ Implemented<br>○ Completed as recommended on Pre-PET<br>○ Significantly modified from Pre-PET<br>○ Not implemented
Substance abuse treatment/support programs	○ Implemented<br>○ Completed as recommended on Pre-PET<br>○ Significantly modified from Pre-PET<br>○ Not implemented
Physical, occupational or speech therapy rehabilitation	○ Implemented<br>○ Completed as recommended on Pre-PET<br>○ Significantly modified from Pre-PET<br>○ Not implemented
Cognitive rehabilitation	○ Implemented<br>○ Completed as recommended on Pre-PET<br>○ Significantly modified from Pre-PET<br>○ Not implemented
**Clinical trial referral**	
Drug therapy or other therapeutic trial for AD (includes amyloid (+) MCI)	○ Implemented<br>○ Completed as recommended on Pre-PET<br>○ Significantly modified from Pre-PET<br>○ Not implemented
Drug therapy or other therapeutic trial for non-AD disorder (please specify)	○ Implemented<br>○ Completed as recommended on Pre-PET<br>○ Significantly modified from Pre-PET<br>○ Not implemented
Referral to observational (non-interventional) research study	○ Implemented<br>○ Completed as recommended on Pre-PET<br>○ Significantly modified from Pre-PET<br>○ Not implemented

PART 3: NEW NON-PHARMACEUTICAL INTERVENTIONS RECOMMENDED AFTER THE PET SCAN WAS COMPLETED

Instructions: Complete only the rows of this table for interventions you recommended since the PET scan. Items that were part of your pre-PET management plan are not shown here. List all recommended interventions, even if they have not yet been implemented.

Note: If the response to Part 1 stated no new non-pharmaceutical interventions were selected, a message appears that says: "You indicated in Part 1 that you have not added NEW non-pharmaceutical interventions. Therefore, Part 3 is omitted.

NON-PHARMACEUTICAL INTERVENTIONS	*8b. Status of interventions*
**Counseling for safety, planning & social support**	
Counseling about safety precautions (home safety, medication monitoring, driving, whether to continue working)	Recommended<br><br>Status<br>○ Implemented<br>○ Not implemented
Counseling about financial/medical decision making, advanced directives	Recommended<br><br>Status<br>○ Implemented<br>○ Not implemented
Referral to community patient/caregiver support resources (e.g. social work, Alzheimer's Association, Family Caregiver Alliance, etc.)	Recommended<br><br>Status<br>○ Implemented<br>○ Not implemented
**Additional diagnostic procedures**	
Neuropsychological testing referral	Recommended<br><br>Status<br>○ Implemented<br>○ Not implemented
**Imaging (brain/head)**	
CT/CTA with/without contrast	Recommended<br><br>Status<br>○ Implemented<br>○ Not implemented

NON-PHARMACEUTICAL INTERVENTIONS	*8b. Status of interventions*
MRI/MRA with/without contrast	Recommended<br><br>Status<br>○ Implemented<br>○ Not implemented
Brain FDG-PET	Recommended<br><br>Status<br>○ Implemented<br>○ Not implemented
DaTscan (Parkinson's disease)	Recommended<br><br>Status<br>○ Implemented<br>○ Not implemented
SPECT for regional cerebral perfusion	Recommended<br><br>Status<br>○ Implemented<br>○ Not implemented
Tau PET	Recommended<br><br>Status<br>○ Implemented<br>○ Not implemented
**Other Laboratory testing or procedures (non-imaging)**	
Lumbar puncture	
AD CSF biomarkers (CSF Aβ42, total tau, phosphorylated tau)	Recommended<br><br>Status<br>○ Implemented<br>○ Not implemented
Other CSF studies	Recommended<br><br>Status<br>○ Implemented<br>○ Not implemented
Serologic (RPR, HIV, auto-antibodies)	Recommended<br><br>Status<br>○ Implemented<br>○ Not implemented

NON-PHARMACEUTICAL INTERVENTIONS	*8b. Status of interventions*
**Genetic tests**	
ApoE genotyping	Recommended<br><br>Status<br>○ Implemented<br>○ Not implemented
Autosomal dominant mutations for AD	Recommended<br><br>Status<br>○ Implemented<br>○ Not implemented
Autosomal dominant mutations for other conditions	Recommended<br><br>Status<br>○ Implemented<br>○ Not implemented
**Other testing**	
EEG	Recommended<br><br>Status<br>○ Implemented<br>○ Not implemented
Polysomnography	Recommended<br><br>Status<br>○ Implemented<br>○ Not implemented
**Referral to other specialists for non-pharmacological interventions**	
Other specialist (e.g. psychiatrist, sleep medicine)	Recommended<br><br>Status<br>○ Implemented<br>○ Not implemented
Surgical intervention (e.g. shunting for hydrocephalus)	Recommended<br><br>Status<br>○ Implemented<br>○ Not implemented

**NON-PHARMACEUTICAL INTERVENTIONS**	***8b. Status of interventions***
Substance abuse treatment/support programs	Recommended<br><br>Status<br>○ Implemented<br>○ Not implemented
Physical, occupational or speech therapy rehabilitation	Recommended<br><br>Status<br>○ Implemented<br>○ Not implemented
Cognitive rehabilitation	Recommended<br><br>Status<br>○ Implemented<br>○ Not implemented
**Clinical trial referral**	
Drug therapy or other therapeutic trial for AD (includes amyloid (+) MCI)	Recommended<br><br>Status<br>○ Implemented<br>○ Not implemented
Drug therapy or other therapeutic trial for non-AD disorder (please specify)	Recommended<br><br>Status<br>○ Implemented<br>○ Not implemented
Referral to observational (non-interventional) research study	Recommended<br><br>Status<br>○ Implemented<br>○ Not implemented

PART 4: STATUS OF PHARMACEUTICAL INTERVENTIONS SELECTED ON THE PRE-PET FORM

Instructions: Report the status of the pharmaceutical interventions you included in this patient's Pre-PET management plan. Complete EVERY ROW of this table, as each of the drugs shown is one you selected on the Pre-PET form.

***These are the items you selected on the Pre-PET form for* Pharmaceutical Interventions**

If there were no pharmaceutical interventions on the pre-PET form, a message appears that says "You did not select any pharmaceutical interventions on the pre-PET form. Therefore, Part 4 is omitted."

Your Pre-PET response is shown in the left column. Status options in the right column will vary depending upon your Pre-PET selection.

PHARMACEUTICAL INTERVENTIONS	*8c. Status of Drug*
**AD Drugs**	
Cholinesterase inhibitors (donepezil, rivastigmine, galantamine)<br><br>○ Patient already on drug; recommended continuing<br>○ Patient already on drug; recommended adjusting<br>○ Patient already on drug; recommended stopping<br>○ Recommended starting this drug	○ Patient [*action from pre-PET*] this drug as recommended on the Pre-PET form<br><br>Actions from Pre-PET are these:<br>○ Continued<br>○ Adjusted<br>○ Stopped<br>○ Started<br><br>○ Management varied from Pre-PET<br>*[Action options available will depend upon your responses on the pre-PET form.]*<br>○ Continued<br>○ Adjusted<br>○ Stopped<br>○ Started
Memantine	***Options are as described above for each item in the table.***
Anti-amyloid Therapeutic<br>○ Aducanumab<br>○ Lecanemab	***Options are as described above for each item in the table.***
**Neuropsychiatric drugs impacting cognition**	
Anti-depressants, mood stabilizers	***Options are as described above for each item in the table.***

PHARMACEUTICAL INTERVENTIONS	*8c. Status of Drug*
Anti-psychotics	***Options are as described above for each item in the table.***
Sedatives/sleep aids	***Options are as described above for each item in the table.***
**Non-neuropsychiatric drugs impacting cognition**	
Anti-cholinergic drugs, opiates, muscle relaxants, etc.	***Options are as described above for each item in the table.***
**Non-neurology/psychiatric pharmacologic therapies***	
Treatment for medical/vascular risk factors (e.g.; anti-platelets, anti-hypertensives, diabetes medications, lipid lowering drugs, etc.)	***Options are as described above for each item in the table.***
**Other neurologic condition**	
Treatment for Parkinson's disease (e.g. carbidopa/levodopa, dopamine agonists, MAO-B inhibitors, others	***Options are as described above for each item in the table.***
Treatment for epilepsy (i.e. anti-epileptics)	***Options are as described above for each item in the table.***
**Targeted therapies**	
Immunosuppressant (auto-immune/ inflammatory encephalopathy)	***Options are as described above for each item in the table.***
Vitamin repletion (nutritional deficiency)	***Options are as described above for each item in the table.***
Antimicrobials (infectious encephalopathy)	***Options are as described above for each item in the table.***

PART 5: NEW PHARMACEUTICAL INTERVENTIONS RECOMMENDED AFTER THE AMYLOID PET SCAN

*Instructions: Complete only the rows of this table for interventions you **recommended** since the PET scan. Items that were part of your pre-PET management plan are not shown here. List **all recommended interventions**, even if they have not yet been implemented.*

If the response to Part 1 stated no new pharmaceutical interventions were selected, a message appears saying: "You indicated in Part 1 that you have not added NEW pharmaceutical interventions. Therefore, Part 5 is omitted."

PHARMACEUTICAL INTERVENTIONS	8c. Status of Drug
**AD Drugs**	
Cholinesterase inhibitors (donepezil, rivastigmine, galantamine)	Recommended<br><br>Status<br>○ Implemented<br>○ Not implemented
Memantine	Recommended<br><br>Status<br>○ Implemented<br>○ Not implemented
Anti-amyloid Therapeutic<br>○ Aducanumab<br>○ Lecanemab	Recommended<br><br>Status<br>○ Implemented<br>○ Not implemented
**Neuropsychiatric drugs impacting cognition**	
Anti-depressants, mood stabilizers	Recommended<br><br>Status<br>○ Implemented<br>○ Not implemented
Anti-psychotics	Recommended<br><br>Status<br>○ Implemented<br>○ Not implemented
Sedatives/sleep aids	Recommended<br><br>Status<br>○ Implemented<br>○ Not implemented
**Non-neuropsychiatric drugs impacting cognition**	
Anti-cholinergic drugs, opiates, muscle relaxants, etc.	Recommended<br><br>Status<br>○ Implemented<br>○ Not implemented
**Non-neurology/psychiatric pharmacologic therapies***	
Treatment for medical/vascular risk factors (e.g.; anti-platelets, anti-hypertensives, diabetes medications, lipid lowering drugs, etc.)	Recommended<br><br>Status<br>○ Implemented<br>○ Not implemented

PHARMACEUTICAL INTERVENTIONS	*8c. Status of Drug*
**Other neurologic condition**	
Treatment for Parkinson's disease (e.g. carbidopa/levodopa, dopamine agonists, MAO-B inhibitors, others	Recommended<br><br>Status<br>○ Implemented<br>○ Not implemented
Treatment for epilepsy (i.e. anti-epileptics)	Recommended<br><br>Status<br>○ Implemented<br>○ Not implemented
**Targeted therapies**	
Immunosuppressant (auto-immune/ inflammatory encephalopathy)	Recommended<br><br>Status<br>○ Implemented<br>○ Not implemented
Vitamin repletion (nutritional deficiency)	Recommended<br><br>Status<br>○ Implemented<br>○ Not implemented
Antimicrobials (infectious encephalopathy)	Recommended<br><br>Status<br>○ Implemented<br>○ Not implemented

9. **A list of the non-pharmaceutical and pharmaceutical interventions you selected on the Pre-PET form, the status of those actions as indicated above, and additional interventions selected above appear in the boxes below. Please certify that these represent your complete management plan for your patient.**

**Pre-PET Management Plan**	**Status of Pre-PET Plan**	**New Interventions**
Non-Pharmaceutical Interventions	Non-Pharmaceutical Interventions	Non-Pharmaceutical Interventions
1.<br>2.<br>3.<br>4.<br>…	1.<br>2.<br>3.<br>4.<br>…	1.<br>2.<br>…
Pharmaceutical Interventions	Pharmaceutical Interventions	Pharmaceutical Interventions
5.<br>6.<br>7.<br>8.<br>…	1.<br>2.<br>3.<br>4.<br>…	1.<br>2.<br>…

I certify that the list above is my complete management plan for this patient, and that the status of items I had selected on the Pre-PET form are accurate.

I wish to make changes to my selections. Return to question 8.

Name of Person responsible for the data on this form: ___________________________________

Form submission date: ____

Case Exception Form

Instructions: This form is to be completed when the patient is unable to complete the study.

---

1. **Type of Exception:**
   - o Pre-PET visit did not occur
   - o Pre-PET form not submitted within 7 days of registration
   - o PET Scan visit did not occur
   - o PET scan not completed within 60 days of submission of pre-PET form
   - o Post-PET follow-up visit did not occur
   - o Post-PET follow-up form not received within 120 days of PET scan completion even though visit did occur
   - o Registration of a case for the same patient by another practice
   - o Other type of error occurred
     Specify other error: ____

2. **Nature of the Exception**

   - o **Registration Issue:**
     - o Duplicate registration
       Under what other case ID number was this participant registered? ___________
     - o Registration data error
     - o Participant registered to different practice and prefers to complete the study at that other practice
       Under what other case ID number was this participant registered? ___________
     - o Dementia doctor responsible for participant's care is not an approved New IDEAS physician
     - o Practice's IRB coverage was not in effect at time of registration
     - o Practice staff person who consented the participant had not completed training in the protection of human subjects in research prior to obtaining this participant's consent
     - o Other: [*Free text]* ____

   - o **Death**

     Date of death: ______/ ______/ _________ *[partial dates allowed, but year is required]*
     Month Day Year

     ☐ Date of death unknown

Cause of death:

- Natural causes (in their sleep or found unresponsive)
- Pneumonia
- Sepsis
- Acute M.I.
- Other infection
- Stroke or brain hemorrhage
- Kidney failure
- Liver failure
- Cancer
- Trauma
- Unknown
- Other: ____

- **Ineligible:** *AFTER registration, participant found not to meet inclusion or exclusion criteria: (select only one, even if more than one is true)*
  - Primary insurance is not Medicare
  - Diagnosis of MCI or dementia has not been verified by dementia expert
  - No head CT or MRI completed within 24 months of date of enrollment
  - No clinical laboratory tests performed within 12 months of enrollment
  - Speaks neither English nor Spanish
  - Refused to sign consent form / improperly consented
  - Normal cognition/ no cognitive testing has been performed
  - Amyloid status already known to Participant or physician
  - Current or previous enrollment in an anti-amyloid therapeutic trial
  - Reason for scan is solely due to pt family history of dementia or APOE status
  - Reason for scan is for non-medical purpose (legal, insurance, employment screening)
  - Cancer, other than non-melanoma skin cancer, requiring active therapy
  - Hip or pelvic fracture within 12 months of enrollment
  - Body weight exceeds scanner limit
  - Life expectancy is less than 24 months
  - Resides in a skilled nursing facility
  - Knowledge of amyloid status, in the opinion of the referring dementia expert, may cause significant psychological harm or otherwise negatively impact the patient or family.

- **PET Facility Error:**
  - Facility completed PET, but failed to submit data forms
  - Scan done before pre-PET form received
  - Scan assessment completed by a physician not approved to read New IDEAS scans
  - Facility not eligible to bill Medicare at time of scan

- **Withdrawn:**
    - Withdrew from care of dementia specialist
    - Dementia Specialist who completed Pre-PET evaluation is not available within 60-120 days of the participant's amyloid PET scan. *[Note: no other physician may complete this form]*
    - Different dementia specialist physician completed pre- and post-PET forms
    - Participant could not tolerate scan
    - Facility did not want to perform scan
    - Withdrew consent for participation in the New IDEAS Study
    - Participant feared radiation exposure
    - Reason related to COVID-19 pandemic [Participant ill with COVID-19, participant quarantined (self- or government imposed), clinic or physician unavailable due to COVID restrictions or personal illness]
    - Facility unable to perform PET as scheduled, and could not reschedule within protocol window
        - Participant's travel to facility could not be arranged (no driver, bad weather, etc.)
        - Participant was ill
        - Insurance or cost issues
            - Facility did not receive prior insurance authorization or doubted reimbursement for other reasons
            - Scan refused when participant learned about possible out-of-pocket costs
        - Problem with tracer on day of scan
        - Problem with scanner on day of scan
        - Participant unable to be still in scanner
        - Participant did not meet scanner criteria (e.g. too heavy, could not lie flat, etc.)
        - Other

          Specify other reason: ____

- **Lost contact with participant** *[Dementia expert or designee is expected to make a minimum of three attempts to contact participant and/or proxy before declaring the participant lost to follow up.]*

  Date of first attempt to contact participant: _____/ _____/ __________

  Date of second attempt to contact participant: _____/ _____/ __________

Name of Person responsible for the data on this form: ___________________________________

Form submission date: ____
