# Supplementary material for: Amyloid PET and clinical management in a diverse, cognitively impaired population: The New IDEAS Study
Source: Alzheimers Dement. 2025 Jul 29;21(7):e70504. doi: 10.1002/alz.70504 (PMC12305457; doi:10.1002/alz.70504)
Supplement: Supplementary file 3 — Supporting Information [file ALZ-21-e70504-s006.docx]

**Supplementary Material 3. Missing Data Imputation Strategy**

We imputed amyloid PET scan results and change in management for all participants with complete pre-PET data as follows:

We first imputed amyloid PET scan result using logistic regression modeling with covariates as listed below. (Note: Because amyloid PET scans of 16/926 participants listed as having an incomplete scan in Fig 1 were actually completed but past the defined time window, we decided to include the actual results rather than impute new ones).

Next, we imputed change in counseling, change in AD drugs, change in non-AD drugs (using logistic regression for each), then calculate overall change in management using the imputed components

We generated 100 imputed datasets using MICE (Multivariate Imputation by Chained Equations) package in R.

We calculated percentages, 95% CIs, and P-values for amyloid PET scan result, change in counseling, change in AD drugs, change in non-AD drugs, and overall change in management using Rubin’s rules

The following 76 non-missing covariates were included in all imputation models.

All models included only fixed effects and no interactions.

- Age (continuous)
- Gender
- Ethnoracial cohort
- Medicare beneficiary type
- Marital status
- Living arrangement
- Highest level of education completed
- Current income
- Income at age 40
- Primary (or preferred) language
- Level of cognitive impairment
- Presentation of cognitive impairment
- MMSE score, or equivalent MMSE score per Roalf et al. (2013) (continuous, take the largest of the two)[1]
- Past or current medical history:
  - Congestive heart failure (with or without atrial fibrillation)
  - Atrial fibrillation
  - History of acute myocardial infarction
  - Ischemic heart disease (including angina pectoris and/or prior coronary artery angioplasty, stent or bypass grafting)
  - Hypertension
  - Dyslipidemia
  - Chronic kidney disease
  - Chronic obstructive pulmonary disease
  - Diabetes
  - Active depression
  - Bipolar affective disorder
  - Schizophrenia
  - Prior history of stroke and/or transient ischemic attack (within past 24 months or more than 24 months ago)
  - Cerebrovascular disease without stroke
  - Previous delirium
  - Epilepsy/seizure disorder
  - Parkinson’s disease
  - Multiple sclerosis
  - Traumatic brain injury (within past 24 months or more than 24 months ago)
  - Tobacco use (past or current)
- Family member diagnosed with Alzheimer's Disease
- Family member diagnosed with other or unknown type of dementia
- Pre-PET differential diagnosis (most likely etiologic cause of cognitive impairment)
- Estimated likelihood that AD pathology is present and causing or contributing to cognitive symptoms
- Pre-PET recommendation for counseling about safety precautions (home safety, medication monitoring, driving)
- Pre-PET recommendation for counseling about financial/medical decision making, advanced directives,
- Pre-PET recommendation for referral to community patient/caregiver support resources (e.g. social work, Alzheimer's Association, Family Caregiver Alliance, etc.)
- Pre-PET recommendation for neuropsychological testing referral
- Pre-PET recommendation for CT/CTA with/without contrast
- Pre-PET recommendation for MRI/MRA with/without contrast
- Pre-PET recommendation for brain FDG-PET
- Pre-PET recommendation for DaTscan (Parkinson's disease)
- Pre-PET recommendation for SPECT for regional cerebral perfusion
- Pre-PET recommendation for tau PET
- Pre-PET recommendation for ApoE genotyping
- Pre-PET recommendation for genetic testing for autosomal dominant mutations for AD
- Pre-PET recommendation for genetic testing for autosomal dominant mutations for other conditions
- Pre-PET recommendation for laboratory testing for AD CSF biomarkers (CSF Aβ42, total tau, phosphorylated tau)
- Pre-PET recommendation for other CSF studies
- Pre-PET recommendation for serologic testing (RPR, HIV, auto-antibodies)
- Pre-PET recommendation for EEG
- Pre-PET recommendation for polysomnography
- Pre-PET recommendation for referral to other specialist (e.g. psychiatrist, sleep medicine)
- Pre-PET recommendation for surgical intervention (e.g. shunting for hydrocephalus)
- Pre-PET recommendation for substance abuse treatment/support programs
- Pre-PET recommendation for physical, occupational or speech therapy rehabilitation
- Pre-PET recommendation for cognitive rehabilitation
- Pre-PET recommendation for drug therapy or other therapeutic trial for AD (includes amyloid (+) MCI)
- Pre-PET recommendation for drug therapy or other therapeutic trial for non-AD disorder
- Pre-PET recommendation for referral to observational (non-interventional) research study
- Pre-PET recommendation for cholinesterase inhibitors (donepezil, rivastigmine, galantamine)
- Pre-PET recommendation for memantine
- Pre-PET recommendation for anti-amyloid therapeutics
- Pre-PET recommendation for anti-depressants, mood stabilizers
- Pre-PET recommendation for anti-psychotics
- Pre-PET recommendation for sedatives/sleep aids
- Pre-PET recommendation for anti-cholinergic drugs, opiates, muscle relaxants, etc.
- Pre-PET recommendation for treatment for medical/vascular risk factors (e.g.; anti-platelets, anti-hypertensives, diabetes medications, lipid lowering drugs, etc.)
- Pre-PET recommendation for treatment for Parkinson's disease (e.g. carbidopa/levodopa, dopamine agonists, MAO-B inhibitors, others
- Pre-PET recommendation for treatment for epilepsy (i.e. anti-epileptics)
- Pre-PET recommendation for immunosuppressant (auto-immune/ inflammatory encephalopathy)
- Pre-PET recommendation for vitamin repletion (nutritional deficiency)
- Pre-PET recommendation for antimicrobials (infectious encephalopathy)

**References**

[1] Roalf DR, Moberg PJ, Xie SX, Wolk DA, Moelter ST, Arnold SE. Comparative accuracies of two common screening instruments for classification of Alzheimer's disease, mild cognitive impairment, and healthy aging. Alzheimers Dement. 2013;9:529-37.
