# Supplementary material for: Amyloid PET and clinical management in a diverse, cognitively impaired population: The New IDEAS Study
Source: Alzheimers Dement. 2025 Jul 29;21(7):e70504. doi: 10.1002/alz.70504 (PMC12305457; doi:10.1002/alz.70504)
Supplement: Supplementary file 4 — Supporting Information [file ALZ-21-e70504-s001.docx]

**Supplementary Table 1. Participant characteristics by ethnoracial group with complete data.**

| **Variable** | **Ethnoracial subgroup** | | | |
| --- | --- | --- | --- | --- |
|  | **Black**  **(N=938)** | **Latinx**  **(N=707)** | **AORE**  **(N=2,718)** | **Total**  **(N=4,363)** |
| Median age (IQR, range), years | 73 (68-79, 35-97) | 75 (70-80, 41-98) | 75 (71-80, 41-95) | 75 (70-80, 35-98) |
| Gender, N (%) |  | | | |
| Female | 605 (64.5) | 438 (62.0) | 1,376 (50.6) | 2,419 (55.4) |
| Male | 333 (35.5) | 269 (38.0) | 1,341 (49.3) | 1,943 (44.5) |
| Transgender male | 0 (0.0) | 0 (0.0) | 1 (0.0) | 1 (0.0) |
| Highest level of education completed, N (%) |  | | | |
| High school graduate/equivalence or below | 318 (33.9) | 392 (55.4) | 578 (21.3) | 1,288 (29.5) |
| Some college or associate degree | 301 (32.1) | 148 (20.9) | 646 (23.8) | 1,095 (25.1) |
| Bachelor's degree | 160 (17.1) | 104 (14.7) | 752 (27.7) | 1,016 (23.3) |
| Postgraduate degree | 159 (17.0) | 63 (8.9) | 742 (27.3) | 964 (22.1) |
| Median MMSE score (IQR) | 21 (19-25) | 21 (19-26) | 25 (22-28) | 24 (20-27) |
| Median MoCA score (IQR) | 18 (13-22) | 18 (13-21) | 21 (18-24) | 20 (15-23) |
| Level of cognitive impairment, N (%) |  | | | |
| MCI | 492 (52.5) | 406 (57.4) | 1,887 (69.4) | 2,785 (63.8) |
| Dementia | 446 (47.5) | 301 (42.6) | 831 (30.6) | 1,578 (36.2) |
| Presentation of cognitive impairment, N (%) |  | | | |
| Atypical | 343 (36.6) | 260 (36.8) | 727 (26.7) | 1,330 (30.5) |
| Typical | 595 (63.4) | 447 (63.2) | 1,991 (73.3) | 3,033 (69.5) |
| Pre‑PET primary differential diagnosis for cause of cognitive impairment |  | | | |
| AD | 793 (84.5) | 634 (89.7) | 2,328 (85.7) | 3,755 (86.1) |
| Non-AD | 145 (15.5) | 73 (10.3) | 390 (14.3) | 608 (13.9) |
| Pre‑PET taking AD drugs^*^, N (%) |  | | | |
| Yes | 419 (44.7) | 275 (38.9) | 1,297 (47.7) | 1,991 (45.6) |
| No | 519 (55.3) | 432 (61.1) | 1,421 (52.3) | 2,372 (54.4) |
| Amyloid PET scan result, N (%) (95% CI) |  | | | |
| Positive | 570 (60.8)  (57.6 to 63.8) | 427 (60.4)  (56.7 to 63.9) | 1858 (68.4)  (66.6 to 70.1) | 2855 (65.4)  (64.0 to 66.8) |
| Negative | 368 (39.2) | 280 (39.6) | 860 (31.6) | 1,508 (34.6) |

Abbreviations: AORE, all other races/ethnicities; AD, Alzheimer’s disease; IQR, interquartile range; MCI, mild cognitive impairment; MMSE, mini-mental state examination; MoCA, Montreal Cognitive Assessment; PET, positron emission tomography.

* The AD drugs that participants could have been taking at the time of the pre-PET visit include cholinesterase inhibitors and memantine. No participants were taking anti amyloid therapeutics at the time of the pre-PET visit (though such treatment might have been recommended to some subjects).

Note: Missing data not imputed.
