# Supplementary material for: Amyloid PET and clinical management in a diverse, cognitively impaired population: The New IDEAS Study
Source: Alzheimers Dement. 2025 Jul 29;21(7):e70504. doi: 10.1002/alz.70504 (PMC12305457; doi:10.1002/alz.70504)
Supplement: Supplementary file 5 — Supporting Information [file ALZ-21-e70504-s004.docx]

**Supplementary Table 2. Change in management by ethnoracial subgroup, level of cognitive impairment, and presentation of cognitive impairment with complete data.**

|  | **Changes in management for each component of the composite endpoint** | | | | | | | | |
| --- | --- | --- | --- | --- | --- | --- | --- | --- | --- |
|  | **Overall change in management** | | | **Change in AD drugs** | | **Change in non-AD drugs** | | **Change in counseling** | |
|  | **N** | **% (95% CI)** | **P value^1^** | **N** | **% (95% CI)** | **N** | **% (95% CI)** | **N** | **% (95% CI)** |
| Ethnoracial subgroup |  | | | | | | | | |
| Black (N=938) |  |  |  |  |  |  |  |  |  |
| MCI (N=492) | 269 | 54.7  (50.3, 59.0) | <0.001 | 195 | 39.6  (35.4, 44.0) | 91 | 18.5  (15.3, 22.2) | 129 | 26.2  (22.5, 30.3) |
| Dementia (N=446) | 244 | 54.7  (50.1, 59.3) | <0.001 | 165 | 37.0  (32.6, 41.6) | 93 | 20.9  (17.3, 24.9) | 74 | 16.6  (13.4, 20.3) |
| Latinx (N=707) |  | | | | | | | | |
| MCI (N=406) | 197 | 48.5  (43.7, 53.4) | <0.001 | 146 | 36.0  (31.4, 40.7) | 66 | 16.3  (13.0, 20.2) | 92 | 22.7  (18.9, 27.0) |
| Dementia (N=301) | 183 | 60.8  (55.2, 66.1) | <0.001 | 127 | 42.2  (36.7, 47.8) | 73 | 24.3  (19.8, 29.4) | 68 | 22.6  (18.2, 27.6) |
| AORE (N=2,718) |  | | | | | | | | |
| MCI (N=1,887) | 1,158 | 61.4  (59.1, 63.5) | <0.001 | 895 | 47.4  (45.2, 49.7) | 364 | 19.3  (17.6, 21.1) | 455 | 24.1  (22.2, 26.1) |
| Dementia (N=831) | 478 | 57.5  (54.1, 60.8) | <0.001 | 362 | 43.6  (40.2, 47.0) | 148 | 17.8  (15.4, 20.6) | 148 | 17.8  (15.4, 20.6) |
| Level and presentation of cognitive impairment |  | | | | | | | | |
| Atypical, MCI (N=786) | 347 | 44.1  (40.7, 47.6) | <0.001 | 240 | 30.5  (27.4, 33.8) | 118 | 15.0  (12.7, 17.7) | 154 | 19.6  (17.0, 22.5) |
| Typical, MCI (N=1,999) | 1,277 | 63.9  (61.8, 66.0) | <0.001 | 996 | 49.8  (47.6, 52.0) | 403 | 20.2  (18.5, 22.0) | 522 | 26.1  (24.2, 28.1) |
| Atypical, Dementia (N=544) | 286 | 52.6  (48.4, 56.7) | <0.001 | 184 | 33.8  (30.0, 37.9) | 114 | 21.0  (17.7, 24.6) | 105 | 19.3  (16.2, 22.8) |
| Typical, Dementia (N=1,034) | 619 | 59.9  (56.8, 62.8) | <0.001 | 470 | 45.5  (42.4, 48.5) | 200 | 19.3  (17.1, 21.9) | 185 | 17.9  (15.7, 20.3) |
| Total (N=4,363) | 2,529 | 58.0  (56.5, 59.4) |  | 1,890 | 43.3  (41.9, 44.8) | 835 | 19.1  (18.0, 20.3) | 966 | 22.1  (20.9, 23.4) |

Abbreviations: AORE, all other races/ethnicities; AD, Alzheimer’s disease; MCI, mild cognitive impairment.

^1^ P values were calculated using a one-sample z-test to test the null hypothesis that the true overall change in management is 30% (for each combination of ethnoracial group and level of cognitive impairment, and for each combination of presentation of cognitive impairment and level of cognitive impairment).

Note: Missing data not imputed.
