# Supplementary material for: Amyloid PET and clinical management in a diverse, cognitively impaired population: The New IDEAS Study
Source: Alzheimers Dement. 2025 Jul 29;21(7):e70504. doi: 10.1002/alz.70504 (PMC12305457; doi:10.1002/alz.70504)
Supplement: Supplementary file 6 — Supporting Information [file ALZ-21-e70504-s011.docx]

**Supplementary Table 3. Ethnoracial identities of individuals from AORE cohort only.**

| **Ethnoracial identity** | **N (%)** |
| --- | --- |
| American Indian or Alaska Native | 28 (0.8) |
| Asian or Asian American | 192 (5.7) |
| Middle Eastern or North African | 50 (1.5) |
| Native Hawaiian or Pacific Islander | 7 (0.2) |
| White or European | 3023 (90.4) |
| Other Race/Ethnicity | 7 (0.2) |
| Preferred not to answer | 49 (1.5) |
| Total | 3343 (100) |

Note: Categories were not mutually exclusive. Participants could select more than one category.
