# Supplementary material for: Amyloid PET and clinical management in a diverse, cognitively impaired population: The New IDEAS Study
Source: Alzheimers Dement. 2025 Jul 29;21(7):e70504. doi: 10.1002/alz.70504 (PMC12305457; doi:10.1002/alz.70504)
Supplement: Supplementary file 7 — Supporting Information [file ALZ-21-e70504-s007.docx]

**Supplementary Table 4. Participant characteristics by level of cognitive impairment.**

| **Variable** | **MCI**  **(N=3,606)** | **Dementia**  **(N=2,151)** | **Total**  **(N=5,757)** |
| --- | --- | --- | --- |
| Median age (IQR, range), years | 74 (70-79, 35-95) | 76 (71-81, 41-98) | 75 (70-80, 35-98) |
| Gender, N (%) |  | | |
| Female | 1,953 (54.2) | 1,266 (58.9) | 3,219 (55.9) |
| Male | 1,650 (45.8) | 885 (41.1) | 2,535 (44.0) |
| Transgender male | 2 (0.1) | 0 (0.0) | 2 (0.0) |
| Prefer not to answer | 1 (0.0) | 0 (0.0) | 1 (0.0) |
| Highest level of education completed, N (%) |  | | |
| High school graduate/equivalence or below | 973 (27.0) | 848 (39.4) | 1,821 (31.6) |
| Some college or associate degree | 887 (24.6) | 519 (24.1) | 1,406 (24.4) |
| Bachelor's degree | 886 (24.6) | 410 (19.1) | 1,296 (22.5) |
| Postgraduate degree | 860 (23.8) | 374 (17.4) | 1,234 (21.4) |
| Median MMSE score (IQR) | 26 (23-28) | 20 (17-23) | 24 (20-27) |
| Median MoCA score (IQR) | 22 (19-25) | 15 (11-19) | 20 (15-23) |
| Presentation of cognitive impairment, N (%) |  | | |
| Atypical | 1,028 (28.5) | 719 (33.4) | 1,747 (30.3) |
| Typical | 2,578 (71.5) | 1,432 (66.6) | 4,010 (69.7) |
| Pre‑PET primary differential diagnosis for cause of cognitive impairment |  | | |
| AD | 3,024 (83.9) | 1,952 (90.7) | 4,976 (86.4) |
| Non-AD | 582 (16.1) | 199 (9.3) | 781 (13.6) |
| Pre‑PET taking AD drugs^*^, N (%) |  |  |  |
| Yes | 1,284 (35.6) | 1,362 (63.3) | 2,646 (46.0) |
| No | 2,322 (64.4) | 789 (36.7) | 3,111 (54.0) |
| Amyloid PET scan result, N (%) |  |  |  |
| Positive | 1,842 (51.1) | 1,321 (61.4) | 3,163 (54.9) |
| Negative | 1,238 (34.3) | 446 (20.7) | 1,684 (29.3) |
| Missing | 526 (14.6) | 384 (17.9) | 910 (15.8) |

Abbreviations: AD, Alzheimer’s disease; IQR, interquartile range; MCI, mild cognitive impairment; MMSE, mini-mental state examination; MoCA, Montreal Cognitive Assessment; PET, positron emission tomography.

* The AD drugs that participants could have been taking at the time of the pre-PET visit include cholinesterase inhibitors and memantine. No participants were taking anti amyloid therapeutics at the time of the pre-PET visit (though such treatment might have been recommended to some subjects).
