# Supplementary material for: Amyloid PET and clinical management in a diverse, cognitively impaired population: The New IDEAS Study
Source: Alzheimers Dement. 2025 Jul 29;21(7):e70504. doi: 10.1002/alz.70504 (PMC12305457; doi:10.1002/alz.70504)
Supplement: Supplementary file 8 — Supporting Information [file ALZ-21-e70504-s013.docx]

**Supplementary Table 5. Participant characteristics by presentation of cognitive impairment.**

| **Variable** | **Atypical**  **(N=1,747)** | **Typical**  **(N=4,010)** | **Total**  **(N=5,757)** |
| --- | --- | --- | --- |
| Median age (IQR, range), years | 73 (69-79, 35-95) | 76 (71-80, 42-98) | 75 (70-80, 35-98) |
| Gender, N (%) |  | | |
| Female | 939 (53.7) | 2,280 (56.9) | 3,219 (55.9) |
| Male | 808 (46.3) | 1,727 (43.1) | 2,535 (44.0) |
| Transgender male | 0 (0.0) | 2 (0.0) | 2 (0.0) |
| Prefer not to answer | 0 (0.0) | 1 (0.0) | 1 (0.0) |
| Highest level of education completed, N (%) |  | | |
| High school graduate/equivalence or below | 629 (36.0) | 1,192 (29.7) | 1,821 (31.6) |
| Some college or associate degree | 422 (24.2) | 984 (24.5) | 1,406 (24.4) |
| Bachelor's degree | 365 (20.9) | 931 (23.2) | 1,296 (22.5) |
| Postgraduate degree | 331 (18.9) | 903 (22.5) | 1,234 (21.4) |
| Median MMSE score (IQR) | 21 (20-26) | 25 (21-27) | 24 (20-27) |
| Median MoCA score (IQR) | 19 (14-23) | 20 (16-23) | 20 (15-23) |
| Level of cognitive impairment, N (%) |  | | |
| MCI | 1,028 (58.8) | 2,578 (64.3) | 3,606 (62.6) |
| Dementia | 719 (41.2) | 1,432 (35.7) | 2,151 (37.4) |
| Pre‑PET primary differential diagnosis for cause of cognitive impairment |  | | |
| AD | 1,341 (76.8) | 3,635 (90.6) | 4,976 (86.4) |
| Non-AD | 406 (23.2) | 375 (9.4) | 781 (13.6) |
| Pre‑PET taking AD drugs^*^, N (%) |  |  |  |
| Yes | 622 (35.6) | 2,024 (50.5) | 2,646 (46.0) |
| No | 1,125 (64.4) | 1,986 (49.5) | 3,111 (54.0) |
| Amyloid PET scan result, N (%) |  |  |  |
| Positive | 818 (46.8) | 2,345 (58.5) | 3,163 (54.9) |
| Negative | 645 (36.9) | 1,039 (25.9) | 1,684 (29.3) |
| Missing | 284 (16.3) | 626 (15.6) | 910 (15.8) |

Abbreviations: AD, Alzheimer’s disease; IQR, interquartile range; MCI, mild cognitive impairment; MMSE, mini-mental state examination; MoCA, Montreal Cognitive Assessment; PET, positron emission tomography.

* The AD drugs that participants could have been taking at the time of the pre-PET visit include cholinesterase inhibitors and memantine. No participants were taking anti amyloid therapeutics at the time of the pre-PET visit (though such treatment might have been recommended to some subjects).
