# Supplementary material for: Amyloid PET and clinical management in a diverse, cognitively impaired population: The New IDEAS Study
Source: Alzheimers Dement. 2025 Jul 29;21(7):e70504. doi: 10.1002/alz.70504 (PMC12305457; doi:10.1002/alz.70504)
Supplement: Supplementary file 11 — Supporting Information [file ALZ-21-e70504-s003.docx]

**Supplementary Table 8. Detailed account of changes in management by presentation of cognitive impairment.**

| **Changes in management for each type of management plan** | **Presentation of cognitive impairment** | | | | |
| --- | --- | --- | --- | --- | --- |
|  | **Atypical**  **(N=1,330)** | | **Typical**  **(N=3,033)** | |  |
|  | **MCI**  **(N=786)** | **Dementia**  **(N=544)** | **MCI**  **(N=1,999)** | **Dementia**  **(N=1,034)** | **Total**  **(N=4,363)** |
| Non-pharmaceutical interventions, % (95% CI) | 50.3  (46.8, 53.7) | 59.7  (55.6, 63.8) | 69.2  (67.2, 71.2) | 62.2  (59.2, 65.1) | 63.0  (61.5, 64.4) |
| Counseling for safety, planning, and social support |  | | | | |
| Counseling about safety precautions | 16.3 | 15.8 | 22.4 | 14.0 | 18.5 |
| Counseling about financial/medical decision making, advanced directives | 15.6 | 15.6 | 23.4 | 14.6 | 18.9 |
| Referral to community patient/caregiver support resources | 17.7 | 19.3 | 24.9 | 19.1 | 21.5 |
| Additional diagnostic procedures |  | | | | |
| Neuropsychological testing referral | 12.5 | 10.1 | 23.9 | 13.8 | 17.7 |
| Imaging (brain/head) |  | | | | |
| CT/CTA with/without contrast | 0.4 | 0.9 | 2.3 | 1.5 | 1.6 |
| MRI/MRA with/without contrast | 2.9 | 3.1 | 7.9 | 4.3 | 5.5 |
| Brain FDG-PET | 8.0 | 15.1 | 10.4 | 10.3 | 10.5 |
| DaTscan | 1.5 | 3.3 | 0.7 | 0.8 | 1.2 |
| SPECT for regional cerebral perfusion | 0.1 | 0.4 | 0.4 | 0.3 | 0.3 |
| Tau PET | 2.0 | 1.3 | 2.9 | 2.3 | 2.4 |
| Genetic tests |  | | | | |
| ApoE genotyping | 7.1 | 5.5 | 11.6 | 10.5 | 9.8 |
| Testing for autosomal dominant mutations for AD | 1.3 | 1.7 | 1.2 | 1.6 | 1.4 |
| Testing for autosomal dominant mutations for other conditions | 1.4 | 2.9 | 0.7 | 1.1 | 1.2 |
| Other laboratory testing or procedures (non-imaging) |  | | | | |
| Lumbar puncture: AD CSF biomarkers | 15.5 | 21.5 | 18.3 | 18.2 | 18.2 |
| Lumbar puncture: other CSF studies | 2.4 | 3.7 | 2.2 | 1.2 | 2.2 |
| Serologic | 0.9 | 2.4 | 1.8 | 0.9 | 1.5 |
| Other tests |  | | | | |
| EEG | 3.8 | 3.5 | 6.3 | 4.2 | 5.0 |
| Polysomnography | 4.3 | 3.7 | 3.9 | 2.5 | 3.6 |
| Referral to other specialists for non-pharmacological interventions |  | | | | |
| Referral for other specialist | 10.3 | 13.1 | 7.4 | 6.5 | 8.4 |
| Referral for surgical intervention | 0.6 | 0.7 | 0.3 | 0.5 | 0.5 |
| Referral for substance abuse treatment/support programs | 0.8 | 0.9 | 0.5 | 0.5 | 0.6 |
| Referral for physical, occupational, or speech therapy rehabilitation | 5.1 | 12.7 | 3.3 | 5.3 | 5.2 |
| Referral for cognitive rehabilitation | 6.4 | 8.8 | 8.2 | 6.4 | 7.5 |
| Clinical trial referral |  | | | | |
| Referral for drug therapy or other therapeutic trial for AD | 12.7 | 11.2 | 23.2 | 15.9 | 18.1 |
| Referral for drug therapy or other therapeutic trial for non-AD disorder | 4.5 | 3.3 | 4.2 | 3.0 | 3.9 |
| Referral to observational research study | 6.5 | 7.5 | 6.0 | 5.0 | 6.0 |
| Pharmaceutical intervention, % (95% CI) | 37.4  (34.1, 40.8) | 45.6  (41.4, 49.8) | 58.0  (55.9, 60.2) | 55.2  (52.2, 58.2) | 52.1  (50.6, 53.6) |
| AD drugs |  | | | | |
| Cholinesterase inhibitors | 18.7 | 21.9 | 25.5 | 20.1 | 22.5 |
| Memantine | 10.6 | 14.9 | 20.3 | 19.6 | 17.7 |
| Anti-amyloid therapeutic | 8.9 | 5.0 | 17.6 | 15.5 | 13.9 |
| Neuropsychiatric drugs impacting cognition |  | | | | |
| Anti-depressants, mood stabilizers | 7.5 | 10.5 | 9.5 | 8.4 | 9.0 |
| Anti-psychotics | 1.3 | 3.3 | 1.3 | 4.4 | 2.2 |
| Sedatives/sleep aids | 2.3 | 3.5 | 2.4 | 3.0 | 2.6 |
| Non-neuropsychiatric drugs impacting cognition |  | | | | |
| Anti-cholinergic drugs, opiates, muscle relaxants, etc. | 0.6 | 0.6 | 1.6 | 0.9 | 1.1 |
| Non-neurology/psychiatric pharmacologic therapies |  | | | | |
| Treatment for medical/vascular risk factors | 4.1 | 3.5 | 7.0 | 3.7 | 5.2 |
| Other neurologic condition |  | | | | |
| Treatment for Parkinson’s disease | 1.3 | 2.0 | 1.3 | 0.3 | 1.1 |
| Treatment for epilepsy | 0.5 | 0.6 | 0.7 | 0.3 | 0.6 |
| Targeted therapies |  | | | | |
| Immunosuppressant | 0.3 | 0.0 | 0.2 | 0.3 | 0.2 |
| Vitamin repletion | 1.7 | 1.7 | 4.2 | 2.1 | 2.9 |
| Antimicrobials | 0.0 | 0.0 | 0.2 | 0.1 | 0.1 |

Abbreviations: AD, Alzheimer’s disease; ApoE, apolipoprotein E; CI, confidence interval; CSF, cerebrospinal fluid; CT/CTA, computed tomography/computed tomography angiography; DaTscan, dopamine transporter scan; EEG, electroencephalogram; FDG-PET, fluorodeoxyglucose positron emission tomography; MCI, mild cognitive impairment; MRI/MRA, magnetic resonance imaging/magnetic resonance angiography; PET, positron emission tomography; SPECT, single-photon emission computed tomography.

Note: All percentages in this table represent the percentage of participants who had a change in management. Missing data not imputed.
