# Supplementary material for: Amyloid PET and clinical management in a diverse, cognitively impaired population: The New IDEAS Study
Source: Alzheimers Dement. 2025 Jul 29;21(7):e70504. doi: 10.1002/alz.70504 (PMC12305457; doi:10.1002/alz.70504)
Supplement: Supplementary file 12 — Supporting Information [file ALZ-21-e70504-s012.docx]

**Supplementary Table 9. Change between pre-PET and post-PET primary differential diagnosis for cause of cognitive impairment by ethnoracial subgroup after multiple imputation.**

|  | **Post‑PET primary differential diagnosis** | | | | | |
| --- | --- | --- | --- | --- | --- | --- |
|  | **Black**  **(N=1,248)** | | **Latinx**  **(N=1,166)** | | **AORE**  **(N=3,343)** | |
| **Pre‑PET primary differential diagnosis** | **AD** | **Non‑AD** | **AD** | **Non‑AD** | **AD** | **Non‑AD** |
| **AD, % (95% CI)** | 59.4% | 25.5%  (22.8%, 28.2%) | 65.4% | 25.6%  (22.6%, 28.7%) | 63.2% | 22.2%  (20.7%, 23.7%) |
| **Non‑AD, % (95% CI)** | 6.8%  (5.3%, 8.2%) | 8.3% | 4.6%  (3.3%, 5.8%) | 4.5% | 7.3%  (6.4%, 8.2%) | 7.3% |

Abbreviations: AORE, all other races/ethnicities; AD, Alzheimer’s disease; CI, confidence interval; PET, positron emission tomography.
