# Supplementary material for: Amyloid PET and clinical management in a diverse, cognitively impaired population: The New IDEAS Study
Source: Alzheimers Dement. 2025 Jul 29;21(7):e70504. doi: 10.1002/alz.70504 (PMC12305457; doi:10.1002/alz.70504)
Supplement: Supplementary file 13 — Supporting Information [file ALZ-21-e70504-s009.docx]

**Supplementary Table 10. Change between pre-PET and post-PET lecanemab use by amyloid scan result for participants enrolled after July 6, 2023.**

|  | **Post‑PET lecanemab use** | | | | | | | | |
| --- | --- | --- | --- | --- | --- | --- | --- | --- | --- |
|  | **Positive amyloid scan result** | | | **Negative amyloid scan result** | | | **Total** | | |
| **Pre‑PET lecanemab use** | **Taking lecanemab** | **Recommend taking lecanemab** | **Neither taking nor recommend taking lecanemab** | **Taking lecanemab** | **Recommend taking lecanemab** | **Neither taking nor recommend taking lecanemab** | **Taking lecanemab** | **Recommend taking lecanemab** | **Neither taking nor recommend taking lecanemab** |
| **Taking lecanemab, N (%)** | 0 | 0 | 0 | 0 | 0 | 0 | 0 | 0 | 0 |
| **Recommend taking lecanemab, N (%)** | 14 (43.8) | 0 | 18 (56.3) | 0 | 0 | 8 (100) | 14 (35) | 0 | 26 (65) |
| **Neither taking nor recommend taking lecanemab, N (%)** | 109 (14.2) | 124 (16.2) | 532 (69.5) | 0 | 1 (0.2) | 418 (99.8) | 109 (9.2) | 125 (10.6) | 950 (80.2) |

Abbreviations: PET, positron emission tomography.

Note: Missing data not imputed.
